# Assessing the global burden of Type 2 diabetes in women of reproductive age

**DOI:** 10.1371/journal.pone.0322787

**Published:** 2025-07-14

**Authors:** Juan Luo, Yun Zhang, Zuojie Luo

**Affiliations:** Department of Endocrinology, The First Affiliated Hospital of Guangxi Medical University, Nanning, China; Facultad Latinoamericana de Ciencias Sociales Mexico, MEXICO

## Abstract

This research critically assesses the global prevalence and trends of Type 2 diabetes (T2D) among women of reproductive age (15–39 years) spanning the period from 1990 to 2021. We conducted an analysis of the age-standardized incidence rates (ASIR), Disability-Adjusted Life Years (DALYs), and Estimated Annual Percentage Change (EAPC) using data from the Global Burden of Diseases (GBD) Study 2021. The global ASIR and DALYs per 100,000 among reproductive-aged women increased from 101.01 to 205.17 and from 113.25 to 198.41, respectively. The EAPC for ASIR was 2.32 [95% Confidence Interval (CI): 2.25 to 2.39], and that for DALYs was 1.76 (95% CI: 1.69 to 1.83), both indicating an upward trend. The increase in T2D prevalence was more prominent in the 25–29 age group and younger women. According to Socio-demographic Index (SDI) category, the highest ASIR and age-standardized DALY rate were observed in low-middle SDI regions (ASIR 104.44; age-standardized DALY rate 136.78). The most significant increases in ASIR were recorded in High-income North America (EAPC = 3.64, 95% CI 3.46 to 3.82) and Cameroon (EAPC = 4.30, 95% CI 4.14 to 4.46). In terms of age-standardized DALY rates, the steepest rises were seen in East Asia (EAPC = 2.71, 95% CI 2.34 to 3.08) and Turkmenistan (EAPC = 4.21, 95% CI 3.89 to 4.52). This study shows a remarkable increase in global T2D burden in women of reproductive age between 1990 and 2021. Interventions should be targeted towards women aged 25–29 years and lifestyle risk factors in low-middle SDI, specifically in countries in North Africa and the Middle East, East Asia, Oceania.

## Introduction

Diabetes mellitus is a chronic metabolic disease characterized by persistent hyperglycemia. It is one of the largest global health problems. The number of adults (20–79 years) with diabetes is expected to increase from 537 million currently to 783 million by the year 2045 [1]. Type 2 diabetes (T2D) is the most common form of diabetes, which is mainly caused by insulin deficiency and insulin resistance [2]. Despite the significant global burden of diabetes, limited attention has been given to women of reproductive age (15–49 years), whose health not only affects their own survival but also family and social development [3]. More than 199 million living women have diabetes all over the world [4], and a large proportion of them are of reproductive age [4,5].

Moreover, there has been a growing trend in the prevalence of T2D among younger individuals [6], and women of reproductive age are particularly affected. Because the onset of T2D occurs mostly during the reproductive years, it is thought to have adverse effects on fertility and pregnancy [7]. It is becoming increasingly apparent that women with T2D are at high risk for reproductive disorders, an area that has not been well studied [8]. The pathophysiology behind reproductive dysfunction in women with diabetes is complex and related to other conditions such as obesity, polycystic ovary syndrome (PCOS), and endogenous or exogenous hyperinsulinemia [9]. Moreover, T2D in women has implications for intergenerational disease transmission [10] and children born to diabetic women have a greater risk of developing T2D themselves [11–13]. Therefore, a comprehensive understanding of the health status of women of reproductive age will provide important clues to help prevent and manage T2D in women of reproductive age.

Comprehensive studies have shed light on the global prevalence and trends of diabetes between 1990 and 2017. Additionally, investigations into diabetes-related mortality among individuals under the age of 25 spanning from 1990 to 2019 have been conducted [14]. Nevertheless, a significant portion of the existing research on T2D forecasting has been limited to individual countries, such as China and Syria’s economic burden research [15,16] or has predominantly addressed the global economic burden of diabetes in the adult population and lack cross regional and cross Socio-demographic Index (SDI) stratified analysis for women of childbearing age [17], without a detailed cross-country or continental analysis. It is worth noting that the disparities in disease burden among countries with varying socioeconomic statuses remain largely unexplored.

We hypothesize that T2D exhibits different epidemiological characteristics among women of reproductive age in various regions globally. The Global Burden of Disease (GBD) 2021 data was used to compare the status of T2D among women of reproductive age between 1990 and 2021 in terms of age-standardized incidence rates (ASIR), Disability-Adjusted Life Years (DALYs), and Estimated Annual Percentage Change (EAPC) and identified the accelerated growth of disease burden in low and middle SDI regions (such as North Africa, the Middle East) and young women (25–29 years old). This analysis is essential for guiding public health policy and formulating effective strategies to alleviate the T2D burden in this key demographic.

## Materials and methods

### Data source

The GBD 2021 project generated estimates for 369 diseases and injuries, including T2D, in 204 countries and territories between 1990 and 2021 [18]. The data were obtained through systematic reviews of multiple data sources including censuses, household surveys, civil registration and vital statistics, and disease registries. The methods and results of GBD 2021 have been previously published and documented in detail. The University of Washington Institutional Review Board has approved a waiver of informed consent for use of identified data in GBD research. No individual participants were involved in this study. ethics approval at https://www.healthdata.org/.

For T2D, we included females aged 15–39 years in our analysis since GBD 2021 provides data for this age group and above. Incidence and DALY for T2D by age, regions, continental, national and socioeconomic status were downloaded from Global Health Data Exchange (https://ghdx.healthdata.org/gbd-results-tool).

SDI is a composite index of the three indicators, including per capita income, educational attainment and total fertility rate in each country. We used the SDI to evaluate the T2D burden in 204 countries worldwide. The SDI was divided into 5 levels according to the socioeconomic development level: high SDI (0.80–1.0), high-middle SDI (0.70–0.80), middle SDI (0.61–0.69), low-middle SDI (0.46–0.60), and low SDI (0–0.45) [19].

### Estimation of T2D burden in women of reproductive age

Diabetes was defined in GBD 2021 as a fasting plasma glucose level of ≥ 126 mg/dL (7.0 mmol/L) or self-reported treatment for diabetes. The nonfatal burden of diabetes was estimated by integrating systematic reviews, survey data and longitudinal studies using DisMod MR-2.1, a Bayesian meta-regression framework. Finer data on T2D were rare, with only 20% of sources providing information on the specific type of diabetes, and thus the nonfatal burden of T2D was calculated by subtracting the burden of type 1 diabetes (defined based on clinical diagnosis, registry or hospital records) from total diabetes estimates.

To estimate the current burden and predict the future burden of T2D among women of reproductive age, we calculated the crude DALYs as the sum of Years Lived with Disability (YLD) and Years of Life Lost (YLL). YLD was calculated using prevalence estimates and disability weights for T2D, which were then corrected for the effects of comorbidity through micro-simulation. YLLs were computed from mortality data as the number of deaths due to T2D multiplied by the standard life expectancy at the age of death. The detailed methods and estimation procedures can be found in the literature [18].

### Statistical analysis

In this study, we report both confidence intervals (CI) and uncertainty intervals (UI) as measures of variability around our estimates. CI are statistical ranges derived from sample data, representing the likelihood that the true population parameter lies within the specified bounds (e.g., 95% CI). In contrast, UI, as used in the GBD framework, account for multiple sources of uncertainty, including sampling error, model uncertainty, and data quality limitations. UI provide a more comprehensive assessment of the potential range of true values for each estimate. Age-standardized rates (ASRs) and 95% CI were computed using the world standard population of GBD 2021 and expressed per 100,000 population. We used the age-standardization method suggested by WHO:


$$Age-standardized\;rate=\frac{\sum_{i}^{A}a_{i}w_{i}}{\sum_{i}^{A}w_{i}}\times 100000$$

Where α_*i*_ is the age-specific rate, and *w*_*i*_ is the weight of the corresponding age group in the chosen standard population (where *i* denotes the *i*-th age group).

The EAPC is an established indicator of the trends in ASRs in a regression model [20,21]. It measures the average percentage change in the ASR across all time intervals. Linear regression was used to determine the EAPC based on the equation y= α + βx+ ε, wherey= ln (ASR) and x= the calendar year. The EAPC was calculated using the formula 100 × [exp(β– 1], and the 95% CI was calculated using the linear regression model. UI for each incidence and DALYs number were calculated based on 1000 iterations, with the 2.5th and 97.5th percentile values defining the 95% UI limits.

We performed correlation analysis between ASRs of T2D and the SDI using data of 2021. Spearman’s rank correlation was used to find out the association between these two variables, which would help to understand the relationship between the burden of T2D with the socio-economic status across all districts.

All statistical analyses were conducted using the R program version 4.0.4, with p values < 0.05 was considered statistically significant.

### Ethics statement

This study did not involve individual participants. The ethics approval can be found at https://www.healthdata.org/.

## Result

### Trends in the burden of Type 2 diabetes mellitus (T2DM) among women of reproductive age, 1990–2021

**Fig 1:**
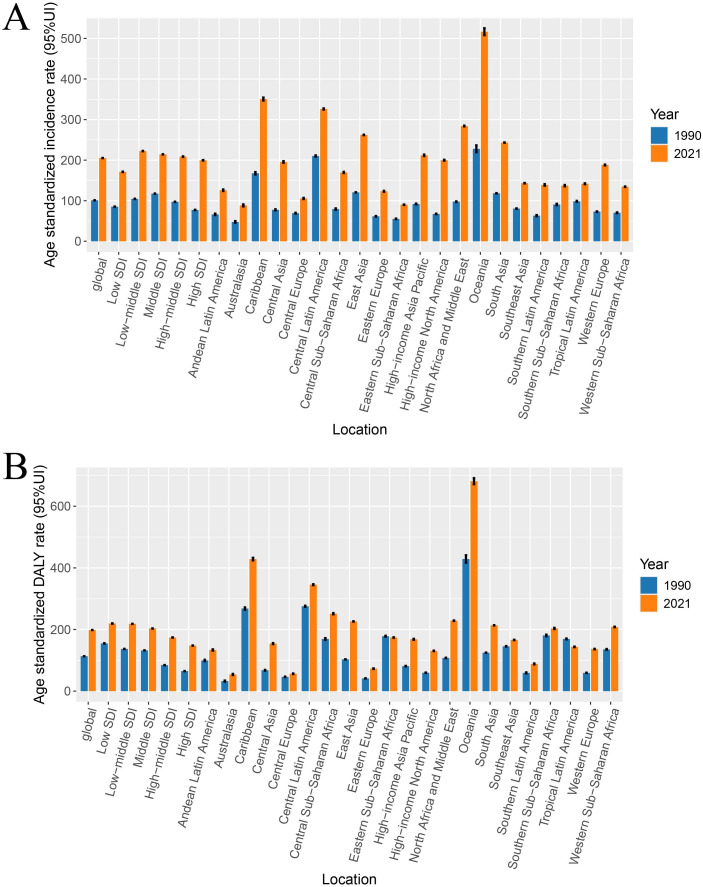
The ASRs of global burden of Type 2 diabetes mellitus among women of childbearing age by region in 1990 and 2021.

The global trend of T2D among women of reproductive age was substantially increased from 1990 to 2021. The number of new cases of T2D worldwide nearly doubled from 1.07 million in 1990 to 3.03 million in 2019 (S3 Table), and the global DALYs for T2D dramatically rose from 1.17 million to 2.98 million (S3 Table). The ASIR of T2D in women of reproductive age was 101.01/100,000 (95% UI: 100.82, 101.20) in 1990, and it increased to 205.17/100,000 (95% UI: 204.93, 205.40; Table 1) in 2021. Additionally, the age-standardized DALY rate significantly rose from 113.25/100,000 (95% UI: 113.05, 113.46) in 1990 to 198.41/100,000 (95% UI: 198.19, 198.64; Table 1 and Fig 1) in 2021, indicating that the overall disease burden of T2D has substantially increased over the past 30 years.

**Table 1:** The ASIR and age-standardized DALY rate of Type 2 diabetes burden in women of reproductive age from 1990 to 2021 in different regions.

	ASIR			Age-standardized DALY rate (per 100000) No.95%UI		
Location	1990 (95%UI)	2021 (95%UI)	1990-2021 EAPC No. (95%CI)	1990 (95%UI)	2021 (95%UI)	1990-2021 EAPC No. (95%CI)
**Global**						
Male						
Female						
Female	101.01 (100.82,101.20)	205.17 (204.93,205.40)	2.32 (2.25,2.39)	113.25 (113.05,113.46)	198.41 (198.19,198.64)	1.76 (1.69,1.83)
**SDI**						
High-middle SDI	97.26 (96.85,97.67)	208.68 (208.05,209.32)	2.63 (2.47,2.80)	84.37 (83.99,84.76)	174.05 (173.51,174.59)	2.42 (2.21,2.64)
High SDI	77.22 (76.80,77.63)	199.60 (198.93,200.26)	3.17 (3.09,3.24)	65.03 (64.66,65.41)	147.82 (147.26,148.37)	2.69 (2.57,2.81)
Low-middle SDI	104.44 (104.00,104.88)	222.23 (221.77,222.70)	2.40 (2.37,2.43)	136.78 (136.27,137.29)	218.27 (217.80,218.73)	1.40 (1.35,1.45)
Low SDI	85.11 (84.49,85.73)	171.05 (170.50,171.61)	2.19 (2.17,2.22)	154.81 (153.96,155.66)	219.33 (218.68,219.97)	0.95 (0.90,1.01)
Middle SDI	117.29 (116.94,117.65)	214.14 (213.71,214.56)	1.90 (1.80,2.00)	132.20 (131.81,132.58)	203.26 (202.86,203.66)	1.31 (1.21,1.41)
**Super_regions**						
Central Europe, eastern Europe, and central Asia	66.41 (65.86,66.97)	138.65 (137.80,139.50)	2.34 (2.28,2.39)	47.27 (46.81,47.74)	90.30 (89.63,90.97)	1.91 (1.80,2.01)
High-income	74.16 (73.76,74.56)	187.34 (186.69,187.99)	3.06 (3.01,3.12)	62.99 (62.63,63.36)	132.63 (132.10,133.17)	2.45 (2.35,2.56)
Latin America and Caribbean	148.38 (147.52,149.24)	235.72 (234.85,236.58)	1.49 (1.39,1.59)	215.35 (214.30,216.39)	249.92 (249.04,250.81)	0.39 (0.24,0.54)
North Africa and Middle East	97.59 (96.79,98.39)	283.98 (283.04,284.92)	3.44 (3.40,3.48)	107.75 (106.90,108.61)	228.64 (227.80,229.48)	2.57 (2.48,2.65)
South Asia	118.21 (117.73,118.69)	243.23 (242.74,243.72)	2.30 (2.26,2.34)	124.76 (124.26,125.26)	213.44 (212.98,213.91)	1.65 (1.60,1.70)
Southeast Asia, east Asia, and Oceania	110.14 (109.80,110.48)	209.64 (209.16,210.12)	2.02 (1.81,2.22)	115.05 (114.70,115.41)	205.88 (205.43,206.32)	1.78 (1.54,2.03)
Sub-Saharan Africa	67.84 (67.30,68.39)	121.87 (121.42,122.33)	1.84 (1.80,1.87)	161.15 (160.28,162.02)	200.25 (199.64,200.85)	0.58 (0.49,0.67)
**Regions**						
Andean Latin America	66.24 (64.37,68.16)	125.79 (123.91,127.70)	2.25 (2.10,2.40)	99.51 (97.18,101.88)	133.47 (131.53,135.43)	0.86 (0.76,0.95)
Australasia	47.64 (45.58,49.78)	88.37 (85.91,90.89)	2.20 (2.13,2.28)	32.61 (30.91,34.37)	54.15 (52.28,56.08)	1.75 (1.64,1.86)
Caribbean	167.56 (164.57,170.61)	350.50 (346.69,354.35)	2.44 (2.40,2.48)	267.98 (264.15,271.86)	428.48 (424.27,432.72)	1.58 (1.49,1.67)
Central Asia	77.42 (75.96,78.91)	195.50 (193.52,197.49)	3.12 (3.02,3.21)	67.70 (66.32,69.11)	154.25 (152.51,156.00)	2.55 (2.36,2.75)
Central Europe	69.24 (68.20,70.29)	105.44 (104.01,106.88)	1.26 (1.22,1.29)	46.25 (45.41,47.11)	56.47 (55.44,57.52)	0.69 (0.55,0.82)
Central Latin America	210.09 (208.50,211.68)	326.13 (324.58,327.69)	1.42 (1.26,1.57)	275.36 (273.51,277.22)	345.43 (343.84,347.03)	0.67 (0.52,0.82)
Central Sub-Saharan Africa	79.27 (77.50,81.07)	169.61 (168.02,171.22)	2.40 (2.36,2.43)	169.23 (166.57,171.93)	251.23 (249.24,253.25)	1.24 (1.20,1.28)
East Asia	120.54 (120.13,120.96)	262.11 (261.39,262.84)	2.63 (2.33,2.93)	103.15 (102.76,103.55)	226.21 (225.61,226.81)	2.71 (2.34,3.08)
Eastern Europe	61.52 (60.79,62.25)	123.26 (122.11,124.42)	2.05 (1.93,2.16)	41.33 (40.74,41.91)	72.94 (72.10,73.79)	1.41 (1.24,1.58)
Eastern Sub-Saharan Africa	55.09 (54.29,55.89)	90.03 (89.38,90.67)	1.42 (1.34,1.49)	178.22 (176.74,179.70)	173.95 (173.03,174.87)	−0.43 (−0.56,−0.29)
High-income Asia Pacific	91.95 (90.92,92.99)	211.78 (209.96,213.61)	2.79 (2.62,2.96)	81.08 (80.12,82.06)	168.18 (166.62,169.75)	2.60 (2.37,2.83)
High-income North America	67.37 (66.71,68.03)	199.74 (198.65,200.84)	3.64 (3.46,3.82)	59.72 (59.11,60.34)	130.63 (129.75,131.51)	2.51 (2.40,2.62)
North Africa and Middle East	97.59 (96.79,98.39)	283.98 (283.04,284.92)	3.44 (3.40,3.48)	107.75 (106.90,108.61)	228.64 (227.80,229.48)	2.57 (2.48,2.65)
Oceania	227.78 (219.35,236.48)	516.42 (507.91,525.04)	2.60 (2.57,2.64)	428.76 (416.74,441.04)	681.20 (671.32,691.19)	1.38 (1.32,1.43)
South Asia	118.21 (117.73,118.69)	243.23 (242.74,243.72)	2.30 (2.26,2.34)	124.76 (124.26,125.26)	213.44 (212.98,213.91)	1.65 (1.60,1.70)
Southeast Asia	80.53 (79.96,81.11)	143.17 (142.54,143.80)	0.95 (0.61,1.28)	145.44 (144.66,146.23)	166.00 (165.32,166.67)	−0.22 (−0.42,−0.02)
Southern Latin America	63.19 (61.60,64.82)	138.83 (136.84,140.86)	2.66 (2.61,2.72)	59.26 (57.72,60.84)	88.15 (86.57,89.75)	1.40 (1.24,1.56)
Southern Sub-Saharan Africa	90.58 (88.76,92.44)	137.07 (135.33,138.84)	1.19 (1.10,1.28)	180.75 (178.09,183.44)	203.43 (201.32,205.56)	0.56 (−0.11,1.23)
Tropical Latin America	98.57 (97.46,99.69)	141.71 (140.63,142.80)	1.16 (1.07,1.24)	169.32 (167.87,170.78)	143.60 (142.52,144.68)	−0.72 (−0.95,−0.48)
Western Europe	73.07 (72.45,73.70)	187.89 (186.81,188.98)	3.09 (3.00,3.17)	59.25 (58.70,59.81)	136.53 (135.66,137.41)	2.71 (2.61,2.80)
Western Sub-Saharan Africa	70.26 (69.37,71.16)	134.40 (133.67,135.14)	2.18 (2.12,2.24)	135.41 (134.14,136.69)	208.24 (207.29,209.18)	1.36 (1.23,1.49)

When categorized by SDI, we observed the highest ASIR and age-standardized DALY rate in regions with a low-middle SDI (ASIR 104.44, 95% UI 104.00 to 104.88; age-standardized DALY rate 136.78, 136.27 to 137.29), while regions with a low SDI demonstrated the lowest ASIR (85.11, 84.49 to 85.73; Table 1). The relationship between ASIR, age-standardized DALY rate, and SDI exhibited an inverse U-shaped curve, where countries with a low-middle and middle SDI experienced higher ASIR and age-standardized DALY rates compared to those with a low SDI (all models, *P* < 0.01; S2 Fig).

Furthermore, the EAPC (Fig 1) showed that both ASIR and age-standardized DALY rates for T2D were increasing, and the increase rate of incidence was slightly higher than the age-standardized DALY rate, which is indicative of the changing burden of T2D among women of reproductive age (S1, S2 Tables and S1 Fig).

### Distribution of the global burden of T2DM among women of reproductive age across different regions and countries, 1990–2021

Our comprehensive assessment from 1990 to 2021 reveals marked regional and national disparities in the burden of T2D among women of reproductive age. Over this time span, incident cases of T2D increased more than twofold in 15 of the 21 regions, with the most substantial rise in North Africa and the Middle East (596%) and the lowest in Central Europe (124%; S3 Table). In 2021, the greatest DALY burdens were in Southeast Asia, east Asia and Oceania (82.81/10,000), South Asia (81.46/10,000), with comparatively lower burdens in Australasia (0.32/10,000) and Central Europe (1.18/10,000 Table 1, S6 Table). ASIR trajectories strongly mirrored these differences.

The EAPC for age-standardized DALY rate and ASIR showed notable increases in several regions. The most prominent increases in ASIR were observed in High-income North America (EAPC = 3.64, 95% CI 3.46 to 3.82), North Africa and the Middle East (EAPC = 3.44, 95% CI 3.40 to 3.48). With regard to DALY rates, the most prominent increases were observed in East Asia (EAPC = 2.71, 95% CI 2.34 to 3.08), Western Europe (EAPC = 2.71, 95% CI 2.61 to 2.80), while small increases or decreases were found in Eastern Sub-Saharan Africa (EAPC = -0.43, 95% CI -0.56 to -0.29), Tropical Latin America (EAPC = -0.72, 95% CI -0.95 to -0.48) (Table 1, S6 Table, S1 Fig).

Our study showed that there were significant differences in T2D among women of reproductive age in countries globally, from 1990 to 2021. The top two countries with the highest age-standardized DALY rates in 2021 were the Solomon Islands (2435.96/100,000, 95% UI 2356.28 to 2517.70) and Kiribati (2275.09/100,000, 95% UI 2099.01 to 2462.45), suggesting a serious burden of T2D. On the other hand, France (66.00/100,000, 95% UI 64.76 to 67.25) and Canada (82.08/100,000, 95% UI 80.28 to 83.91; S2, S4 and S7 Tables) had the lowest. The trend was similar for the ASIR. The Marshall Islands (1195.29/100,000, 95% UI 1023.04 to 1389.15) and American Samoa (1172.83/100,000, 95% UI 993.20 to 1377.57) had the highest rates, while France (95.45/100,000, 95% UI 93.90 to 97.02) and Nigeria (98.07/100,000, 95% UI 97.14 to 99.02; S1 and S7 Tables) had the lowest.

Large increases in the EAPC for age-standardized DALY rates were found in Turkmenistan (EAPC = 4.21, 95% CI 3.89 to 4.52), Mauritius (EAPC = 4.24, 95% CI 3.83 to 4.66). Large decreases in the EAPC for age-standardized DALY rates were found in Rwanda (EAPC = -2.67, 95% CI -3.08 to -2.26), Ethiopia (EAPC = -2.01, 95% CI -2.27 to -1.75). The largest increases in the EAPC for ASIR were observed in Cameroon (EAPC = 4.30, 95% CI 4.14 to 4.46), Egypt (EAPC = 4.41, 95% CI 4.32 to 4.50) (S1, S2, S5 Tables).

### Age distribution of the global burden of T2DM among women of reproductive age in 2019

**Fig 2:**
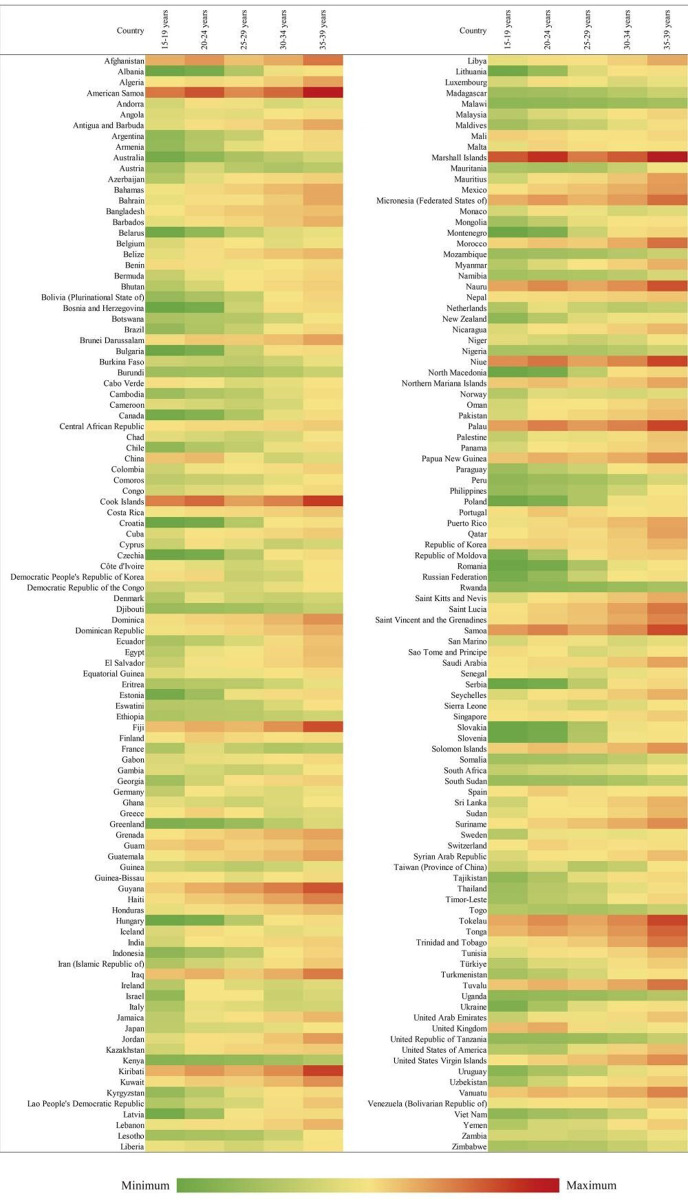
Global Heatmap of incidence of Type 2 diabetes mellitus rates among women of childbearing age in 2021 by country and age group.

The incidence and DALY rates of T2D among women of reproductive age in 2021 increased with age. The increase was more rapid, starting from age 25–29 years, especially for the global burden (Fig 2).

The age group-wise analysis showed that the incidence rate of T2D among women of reproductive age had a slow increase in the youngest group (15–19 years) and then increased rapidly, reaching a peak in the 25–29 years’ age group (S3 Fig). Thereafter, it declined slightly in older groups of 35–39 years (Fig 2).

### The relationship between the burden of T2DM in women of reproductive age and socioeconomic development levels, 1990–2021

The analysis of age-standardized incidence and DALY rates for T2D shows notable socioeconomic patterning. When viewed by SDI quintiles, very different trends are apparent in the burden of T2D. In high SDI countries (e.g., Western Europe and North America), there is an upward trend in the rates of T2D over the period 1990–2021 (S5 Table).

When disaggregated by age group, countries with SDI > 0.80 have a higher incidence of T2D in 15–24 year age groups as well as an increase over time, indicating an early onset of disease related to lifestyle practices more common in high income countries. On the other hand, in countries with SDI < 0.55, the burden of T2D increases with age, and we see large increases in DALY rates in females aged 35–39 years and older, indicating both a later onset of disease as well as potentially lack of early life interventions.

## Discussion

While this study provides valuable insights into the global burden and trends of T2DM among women of reproductive age, it is important to note that the findings primarily identify associations and trends rather than establishing direct causal relationships. The observed increases in ASIR and DALYs across different regions and socioeconomic groups highlight potential correlations with factors such as urbanization, lifestyle changes, and healthcare access. However, these associations do not imply causation. This trend is closely associated with adverse lifestyle choices, Similarly, the pronounced increase in T2DM incidence among younger women aged 25–29 years underscores the potential role of adverse lifestyle factors, such as poor dietary habits, physical inactivity, and obesity [22], leading to interrelated health issues such as insulin resistance, PCOS, and obesity [22].

The risk factors for T2D in women of reproductive age are diverse, encompassing traditional factors such as age, and emerging ones like lifestyle-related obesity and sedentary behavior [23]. Additionally, gestational diabetes mellitus (GDM), PCOS, hypertension, dyslipidemia, and racial/ethnic predispositions also play significant roles [24]. Insulin resistance and beta-cell dysfunction are central to the pathogenesis of T2DM, particularly in women of childbearing age, where female-specific hormonal changes further complicate their roles. Insulin resistance, often driven by obesity and sedentary lifestyles, is exacerbated during specific phases of the menstrual cycle, such as the luteal phase, when progesterone levels are elevated, potentially impairing insulin sensitivity [7]. Additionally, conditions like PCOS, which is closely linked to insulin resistance, disproportionately affect this demographic, creating a vicious cycle of metabolic dysregulation and reproductive dysfunction [9]. Pregnancy introduces another layer of complexity, as gestational hormones, including human placental lactogen and progesterone, induce physiological insulin resistance to ensure fetal nutrient supply, but this can precipitate GDM in predisposed individuals [24]. Beta-cell dysfunction, on the other hand, reflects an inability to compensate for increased insulin demands, particularly during pregnancy, leading to hyperglycemia [10]. The interplay between these mechanisms underscores the heightened vulnerability of women of childbearing age to T2DM, emphasizing the need for targeted interventions that consider hormonal fluctuations and reproductive health [8].

Lifestyle interventions play a pivotal role in the management and treatment of T2DM among women of childbearing age, addressing both prevention and disease control. Dietary control, regular physical activity, and weight management are foundational strategies that can significantly improve glycemic control, reduce insulin resistance, and mitigate the risk of diabetes-related complications in this demographic [23]. For instance, adopting a balanced diet rich in whole grains, lean proteins, healthy fats, and fiber while limiting processed foods and added sugars has been shown to lower blood glucose levels and improve metabolic outcomes [24]. Similarly, regular exercise, including both aerobic and resistance training, enhances insulin sensitivity and aids in weight management, which is particularly critical given the rising prevalence of obesity—a major risk factor for T2DM—among younger populations [25]. Weight management, especially in women with PCOS, not only improves reproductive health but also reduces the overall burden of metabolic disorders [26]. Furthermore, lifestyle modifications are cost-effective and empower women to take an active role in managing their health, which is crucial during reproductive years when the implications extend to future generations [27]. Despite these benefits, adherence to lifestyle interventions remains a challenge, necessitating culturally tailored public health strategies and increased awareness campaigns to address barriers such as socioeconomic constraints, lack of education, and limited access to resources [28]. By prioritizing lifestyle interventions, healthcare systems can foster long-term improvements in the health outcomes of women with T2DM, ultimately reducing the global burden of the disease.

The WHO Global Action Plan on Diabetes and the WHO Global Diabetes Compact formulated in 2021, are a part of a global response to address this growing concern [25]. Our findings suggest more than a two-fold increase in incident cases of T2D in 17 out of 21 regions between 1990 and 2021 signifying its increasing burden. At the same time, the rise in incidence and DALYs of T2D globally, emphasizes the growing challenge of this disease [26]. The striking rise in the T2D ASIR suggests an increase in new cases due to better diagnosis and/or a real increase in disease burden. The GBD 2021 Diabetes Collaborators projected that by 2050, 89 (43.6%) of 204 countries and territories will have an age-standardized prevalence > 10% [27]. In contrast to the ASIR, the relatively slower increase in DALYs suggests improvements in the management and care of T2D. As demonstrated in a previous publication [28], huge strides have been made in the treatment of hyperglycemia in recent years with the availability of many new agents which have widened the therapeutic options for T2D. These developments are likely to have led to better outcomes for the disease as is suggested by the slower increase in DALYs.

The incorporation of innovative glucose-lowering medications, such as GLP-1 receptor agonists and SGLT2 inhibitors, represents a promising advancement in the management of T2DM among women of childbearing age. These therapeutic agents not only effectively lower blood glucose levels but also offer additional benefits that are particularly relevant to this demographic. GLP-1 receptor agonists have been shown to promote weight loss and reduce cardiovascular risk, addressing two critical concerns in women with T2DM who often face obesity and heightened cardiovascular vulnerability [28]. Similarly, SGLT2 inhibitors have demonstrated significant cardioprotective and renoprotective effects, which are crucial given the increased risk of cardiovascular and renal complications in women with T2DM [29]. Furthermore, these medications may mitigate some of the reproductive health challenges associated with T2DM, such as PCOS, by improving insulin sensitivity and reducing hyperandrogenism [30]. However, careful consideration is required when prescribing these agents to women of childbearing age, particularly during pregnancy, as their safety profiles in gestational contexts remain under investigation [31]. Despite these challenges, the potential of GLP-1 receptor agonists and SGLT2 inhibitors to improve long-term health outcomes underscores the importance of personalized treatment strategies tailored to the unique physiological and reproductive needs of this population.

The inverse U-shaped relationship between SDI and T2DM burden suggests that socioeconomic development may initially exacerbate the disease burden before improvements in healthcare and preventive measures lead to a decline in high-income settings. While this pattern aligns with existing literature on the epidemiological transition [29], further research is needed to explore the underlying mechanisms driving these trends. Health outcomes and disease burden were strongly linked to socioeconomic development as measured by the SDI [29]. Our analysis further suggests strong geographical heterogeneity of the T2D trends for women of reproductive age across various SDI categories, showing an inverted-U-shaped relationship between SDI and ASIR, age-standardized DALY rate. This suggests that age standardized rates of T2D initially deteriorate with countries’ development but fall in the later stages of development suggesting possibly improved access to care. High-income North America, North Africa and Middle East, East Asia, Western Europe had the largest annual increases in ASIR and age-standardized DALY rates. In 2021, four Oceanian countries (Solomon Islands, Kiribati, Marshall Islands, American Samoa) had the highest age-standardized DALY rates and ASIR. The findings are consistent with the Lancet report that some regions have experienced high diabetes prevalence rates in 2021 [27]. These high prevalence rates of type 2 diabetes may be due to the genetic susceptibility of certain populations such as South Asians [30]. Meanwhile, the diabetes burden in low- middle-income countries (LMICs) is exacerbated by economic and sociopolitical factors [31], such as limited health budgets for diabetes control and lack of subsidization for pharmacological treatments. In LMICs, less than 10% of people with diabetes are estimated to be receiving full treatment coverage. For example, Oceania has the lowest medication coverage globally despite experiencing extremely high prevalence rates [32].

But, we observed fast increase in T2D burden among reproductive aged women in Turkmenistan, Mauritius. These countries have witnessed a remarkable increase in T2D prevalence and therefore request immediate public health approaches to reduce the ambient pollution [33]. Our results suggest region-specific approaches with local socioeconomic conditions to combat the rising T2D burden.

Our study observed a pronounced upsurge in both incidence and DALYs among women of reproductive age, predominantly in the 35–39 age bracket globally. This concurs with existing literature that underscores age as a notable risk factor for T2D [34,35]. The heightened peaks were recorded among those aged between 25 and 29 years. Suboptimal glycemic control during the typical reproductive period for women (i.e., aged 25–29 years) may precipitate hyperglycemia during pregnancy and detrimental outcomes for offspring. Consequently, there is an imperative need to bolster the prevention and management of type 2 diabetes among women within this age range [36].

The strength of this study is that it used data from GBD 2021 to analyze the trends of T2D from 1990 to 2021, which allowed us to globally examine the trends of T2D among women of reproductive age. In addition, ASIR, DALY and EAPC indicators make our results more representative of T2D in different regions and age groups. However, there are still some limitations in our research. First, because this analysis is based on the GBD 2021, our results are affected by the methodological flaws of this study. For example, when there is a lack of data, the results depend on the predictive validity of modeling. When there was data, differences in definitions and measurement methods (for example, different case definitions from different sources) may have led to bias. Although the researchers of GBD 2021 study have used many methods to reduce bias and error, residual bias is still possible. In addition, we estimated CI instead of UI for rates after standardization by age. Second, gestational diabetes is not included in our study because it is classified into other maternal disorders in GBD. Therefore, our conclusions need to be carefully interpreted and further studies are needed to confirm our results.

## Conclusion

This study highlights a significant increase in the global burden of T2DM among women of reproductive age from 1990 to 2021, with marked rises in ASIR and DALYs. Our findings reveal complex associations between T2DM burden and socioeconomic development, emphasizing the need for age-specific and region-specific public health strategies. By focusing on these priorities, policymakers and healthcare providers can develop comprehensive, context-sensitive strategies to effectively address the growing challenge of T2DM among women of reproductive age, particularly in regions like North Africa, the Middle East, East Asia, and Oceania

The escalating prevalence of T2DM, particularly among younger women aged 25–29 years, underscores the urgent need for targeted interventions addressing lifestyle factors such as dietary control, physical activity, and weight management. While our analysis points to potential drivers of the increasing burden, including socioeconomic transitions and adverse lifestyle changes, further research is required to confirm causal relationships and refine intervention strategies. Future studies should employ robust methodologies, including longitudinal designs and real-world data, to provide deeper insights into the causal pathways underlying the observed trends.

## Supporting information

S1 Fig Trends in EAPCs of Type 2 diabetes mellitus among women of childbearing age by region from 1990 to 2021. Panel A: DALY (disability-adjusted life-year); Panel B: ASIR (age-standardized incidence rate).
(DOCX)

S2 Fig The correlation of SDI and age-standardized rates of type 2 diabetes mellitus rates among women of childbearing age in 2021. A. ASIR (age-standardized incidence rate); B. DALY (disability-adjusted life-year).
(DOCX)

S3 Fig Global Heatmap of DALY (disability-adjusted life-year) of Type 2 diabetes mellitus rates among women of childbearing age in 2021 by country and age group. (DOCX)

S1 Table The incidence and incidence rate of type 2 diabetes mellitus burden in women of childbearing age in 1990 and 2021, and its temporal trends from 1990 to 2021. (DOCX)

S2 Table The DALY and age-standardized DALY rate of type 2 diabetes mellitus burden in women of childbearing age in 1990 and 2021, and its temporal trends from 1990 to 2021. (DOCX)

S3 Table The number and its trend of incidence and DALY for type 2 diabetes burden mellitus in women of childbearing age in 1990 and 2021 in different regions. (DOCX)

S4 Table The number and its trend of incidence and DALY type 2 diabetes mellitus burden in women of childbearing age in 1990 and 2021, by countries and regions. (DOCX)

S5 Table The comparative analysis of age-standardized type 2 diabetes mellitus incidence and DALY rates by country and region, 1990 and 2021. (DOCX)

S6 Table The top three and the bottom three regions of type 2 diabetes mellitus burden in women of childbearing age. (DOCX)

S7 Table The top three and the bottom three countries of type 2 diabetes mellitus burden in women of childbearing age. (DOCX)

## References

[1] Xie J, Wang M, Long Z, Ning H, Li J, Cao Y, et al. Global burden of type 2 diabetes in adolescents and young adults, 1990-2019: systematic analysis of the Global Burden of Disease Study 2019. BMJ. 2022;379:e072385. doi: 10.1136/bmj-2022-072385 36740855 PMC9727920

[2] Control CfD, Prevention. National diabetes statistics report: estimates of diabetes and its burden in the United States, 2014. Atlanta, GA: US Department of Health and Human Services; 2014.

[3] Rodin J, Ickovics JR. Women’s health. Review and research agenda as we approach the 21st century. Am Psychol. 1990;45(9):1018–34. doi: 10.1037//0003-066x.45.9.1018 2221569

[4] Sadikot S, Purandare CN, Cho NH, Hod M. FIGO-IDF joint statement and declaration on hyperglycemia in pregnancy. Diabetes Res Clin Pract. 2018;145:1–4. doi: 10.1016/j.diabres.2018.02.031 30001825

[5] Bijelic R, Balaban J, Milicevic S, Sipka SU. The association of obesity and microvascular complications with glycemic control in patients with type 2 diabetes mellitus. Med Arch. 2020;74(1):14–8. doi: 10.5455/medarh.2020.74.14-18 32317828 PMC7164726

[6] Collaborators G. Diabetes mortality and trends before 25 years of age: an analysis of the Global Burden of Disease Study 2019. 2022. 10.1016/S2213-8587(21)00349-1 PMC8860753 35143780

[7] Thong EP, Codner E, Laven JSE, Teede H. Diabetes: a metabolic and reproductive disorder in women. Lancet Diabetes Endocrinol. 2020;8(2):134–49. doi: 10.1016/S2213-8587(19)30345-6 31635966

[8] Jenum AK, Sommer C, Sletner L, Mørkrid K, Bærug A, Mosdøl A. Adiposity and hyperglycaemia in pregnancy and related health outcomes in European ethnic minorities of Asian and African origin: a review. Food Nutr Res. 2013;57:10.3402/fnr.v57i0.18889. doi: 10.3402/fnr.v57i0.18889 23467680 PMC3585772

[9] Livadas S, Anagnostis P, Bosdou JK, Bantouna D, Paparodis R. Polycystic ovary syndrome and type 2 diabetes mellitus: a state-of-the-art review. World J Diabetes. 2022;13(1):5–26. doi: 10.4239/wjd.v13.i1.5 35070056 PMC8771268

[10] Clausen TD, Mathiesen ER, Hansen T, Pedersen O, Jensen DM, Lauenborg J, et al. High prevalence of type 2 diabetes and pre-diabetes in adult offspring of women with gestational diabetes mellitus or type 1 diabetes: the role of intrauterine hyperglycemia. Diabetes Care. 2008;31(2):340–6. doi: 10.2337/dc07-1596 18000174

[11] Nomaguchi K, Milkie MA. Parenthood and well-being: a decade in review. J Marriage Fam. 2020;82(1):198–223. doi: 10.1111/jomf.12646 32606480 PMC7326370

[12] Prochaska JJ, Spring B, Nigg CR. Multiple health behavior change research: an introduction and overview. Prev Med. 2008;46(3):181–8. doi: 10.1016/j.ypmed.2008.02.001 18319098 PMC2288583

[13] Langer A, Meleis A, Knaul FM, Atun R, Aran M, Arreola-Ornelas H, et al. Women and health: the key for sustainable development. Lancet. 2015;386(9999):1165–210. doi: 10.1016/S0140-6736(15)60497-4 26051370

[14] Liu J, Ren Z-H, Qiang H, Wu J, Shen M, Zhang L, et al. Trends in the incidence of diabetes mellitus: results from the Global Burden of Disease Study 2017 and implications for diabetes mellitus prevention. BMC Public Health. 2020;20(1):1415. doi: 10.1186/s12889-020-09502-x 32943028 PMC7500018

[15] Liu J, Liu M, Chai Z, Li C, Wang Y, Shen M, et al. Projected rapid growth in diabetes disease burden and economic burden in China: a spatio-temporal study from 2020 to 2030. Lancet Regional Health–Western Pacific. 2023;33. 10.1016/j.lanwpc.2023.100700 PMC9932123 36817869

[16] Al Ali R, Mzayek F, Rastam S, M Fouad F, O’Flaherty M, Capewell S, et al. Forecasting future prevalence of type 2 diabetes mellitus in Syria. BMC Public Health. 2013;13:507. doi: 10.1186/1471-2458-13-507 23705638 PMC3673829

[17] Bommer C, Sagalova V, Heesemann E, Manne-Goehler J, Atun R, Bärnighausen T, et al. Global economic burden of diabetes in adults: projections from 2015 to 2030. Diabetes Care. 2018;41(5):963–70. doi: 10.2337/dc17-1962 29475843

[18] GBD 2019 Diseases and Injuries Collaborators. Global burden of 369 diseases and injuries in 204 countries and territories, 1990-2019: a systematic analysis for the Global Burden of Disease Study 2019. Lancet. 2020;396(10258):1204–22. doi: 10.1016/S0140-6736(20)30925-9 33069326 PMC7567026

[19] GBD 2019 Diabetes and Air Pollution Collaborators. Estimates, trends, and drivers of the global burden of type 2 diabetes attributable to PM2·5 air pollution, 1990-2019: an analysis of data from the Global Burden of Disease Study 2019. Lancet Planet Health. 2022;6(7):e586–600. doi: 10.1016/S2542-5196(22)00122-X 35809588 PMC9278144

[20] Hankey BF, Ries LA, Kosary CL, Feuer EJ, Merrill RM, Clegg LX, et al. Partitioning linear trends in age-adjusted rates. Cancer Causes Control. 2000;11(1):31–5. doi: 10.1023/a:1008953201688 10680727

[21] Liu Z, Jiang Y, Yuan H, Fang Q, Cai N, Suo C, et al. The trends in incidence of primary liver cancer caused by specific etiologies: results from the Global Burden of Disease Study 2016 and implications for liver cancer prevention. J Hepatol. 2019;70(4):674–83. doi: 10.1016/j.jhep.2018.12.001 30543829

[22] Barber TM, Franks S. Obesity and polycystic ovary syndrome. Clin Endocrinol (Oxf). 2021;95(4):531–41. doi: 10.1111/cen.14421 33460482

[23] American Diabetes Association Professional Practice Committee. 2. Classification and diagnosis of diabetes: standards of medical care in diabetes-2022. Diabetes Care. 2022;45(Suppl 1):S17–38. doi: 10.2337/dc22-S002 34964875

[24] Nandhini M. A study of serum adiponectin as a bio-marker for predicting gestational diabetes mellitus: Stanley Medical College, Chennai; 2018.

[25] Organization WH. Global action plan for the prevention and control of noncommunicable diseases 2013-2020: World Health Organization; 2013.

[26] Mukherjee SM, Dawson A, Carey KM. Preconception care for individuals with diabetes. Diabetes. 2023.

[27] GBD 2021 Diabetes Collaborators. Global, regional, and national burden of diabetes from 1990 to 2021, with projections of prevalence to 2050: a systematic analysis for the Global Burden of Disease Study 2021. Lancet. 2023;402(10397):203–34. doi: 10.1016/S0140-6736(23)01301-6 37356446 PMC10364581

[28] Tsoutsouki J, Wunna W, Chowdhury A, Chowdhury TA. Advances in the management of diabetes: therapies for type 2 diabetes. Postgrad Med J. 2020;96(1140):610–8. doi: 10.1136/postgradmedj-2019-137404 32467106

[29] GBD 2019 Demographics Collaborators. Global age-sex-specific fertility, mortality, healthy life expectancy (HALE), and population estimates in 204 countries and territories, 1950-2019: a comprehensive demographic analysis for the Global Burden of Disease Study 2019. Lancet. 2020;396(10258):1160–203. doi: 10.1016/S0140-6736(20)30977-6 33069325 PMC7566045

[30] Misra A, Ramchandran A, Jayawardena R, Shrivastava U, Snehalatha C. Diabetes in South Asians. Diabet Med. 2014;31(10):1153–62. doi: 10.1111/dme.12540 24975549

[31] Sun H, Saeedi P, Karuranga S, Pinkepank M, Ogurtsova K, Duncan BB, et al. IDF Diabetes Atlas: global, regional and country-level diabetes prevalence estimates for 2021 and projections for 2045. Diabetes Res Clin Pract. 2022;183:109119. doi: 10.1016/j.diabres.2021.109119 34879977 PMC11057359

[32] Flood D, Seiglie JA, Dunn M, Tschida S, Theilmann M, Marcus ME, et al. The state of diabetes treatment coverage in 55 low-income and middle-income countries: a cross-sectional study of nationally representative, individual-level data in 680 102 adults. Lancet Healthy Longev. 2021;2(6):e340–51. doi: 10.1016/s2666-7568(21)00089-1 35211689 PMC8865379

[33] GBD 2019 Risk Factors Collaborators. Global burden of 87 risk factors in 204 countries and territories, 1990-2019: a systematic analysis for the Global Burden of Disease Study 2019. Lancet. 2020;396(10258):1223–49. doi: 10.1016/S0140-6736(20)30752-2 33069327 PMC7566194

[34] Fazeli PK, Lee H, Steinhauser ML. Aging is a powerful risk factor for type 2 diabetes mellitus independent of body mass index. Gerontology. 2020;66(2):209–10. doi: 10.1159/000501745 31505500 PMC7056531

[35] Suastika K, Dwipayana P, Semadi MS, Kuswardhani RT. Age is an important risk factor for type 2 diabetes mellitus and cardiovascular diseases. Glucose Tolerance. 2012;5:67-80.

[36] Kampmann U, Knorr S, Fuglsang J, Ovesen P. Determinants of maternal insulin resistance during pregnancy: an updated overview. J Diabetes Res. 2019;2019:5320156. doi: 10.1155/2019/5320156 31828161 PMC6885766

