# Supplementary material for: Assessing the global burden of Type 2 diabetes in women of reproductive age
Source: PLoS One. 2025 Jul 14;20(7):e0322787. doi: 10.1371/journal.pone.0322787 (PMC12258576; doi:10.1371/journal.pone.0322787)
Supplement: S1 Fig — Panel A: DALY (disability-adjusted life-year); Panel B: ASIR (age-standardized incidence rate). (DOCX) [file pone.0322787.s001.docx]

**S1 Fig. Trends in EAPCs of Type 2 Diabetes Mellitus Among Women of Childbearing Age by Region from 1990 to 2021.** Panel A: DALY (disability-adjusted life-year); Panel B: ASIR (age-standardized incidence rate).

**
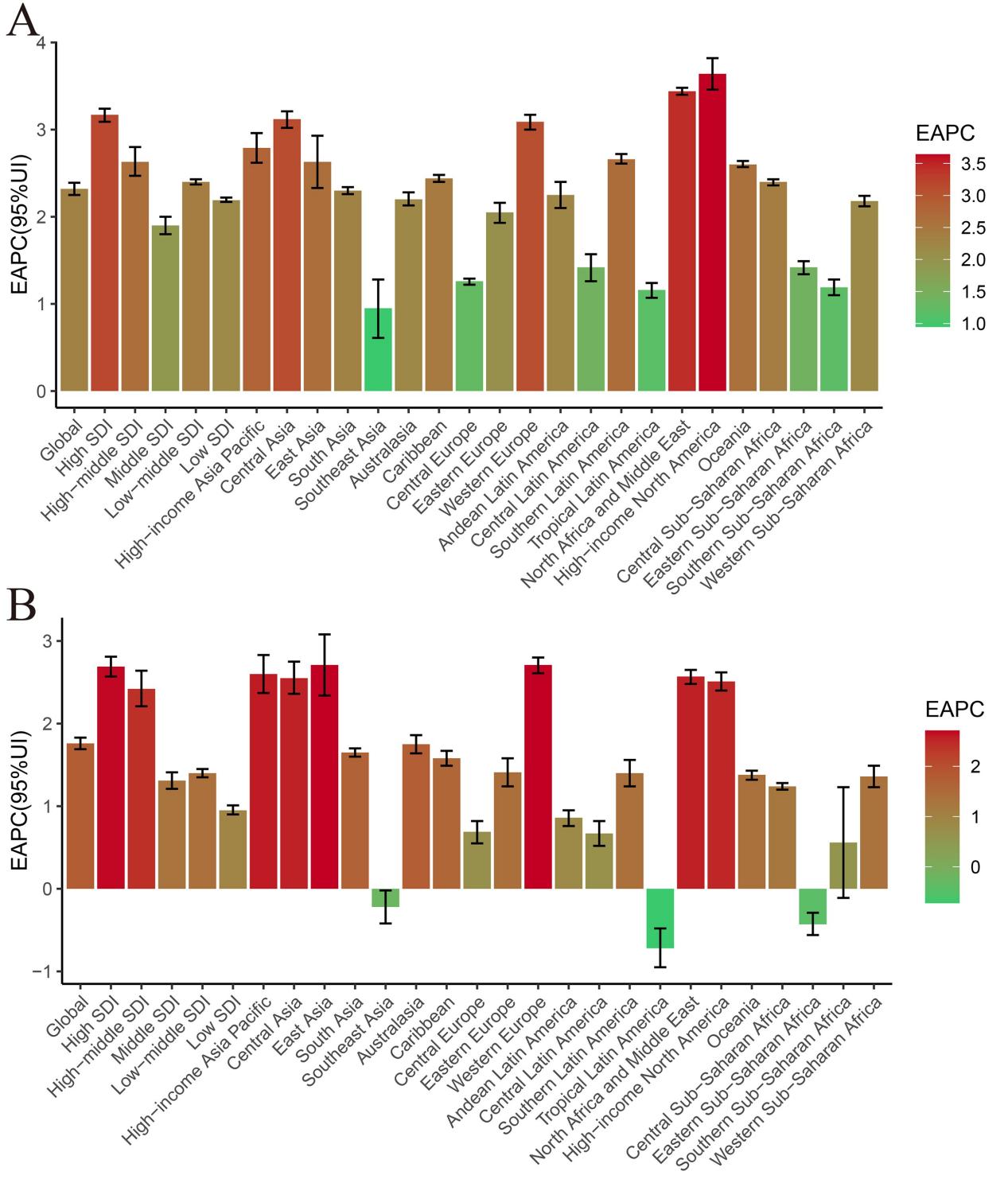
**
