# Supplementary material for: Assessing the global burden of Type 2 diabetes in women of reproductive age
Source: PLoS One. 2025 Jul 14;20(7):e0322787. doi: 10.1371/journal.pone.0322787 (PMC12258576; doi:10.1371/journal.pone.0322787)

**S2 Fig. The correlation of SDI and Age-standardized rates of Type 2 Diabetes Mellitus Rates Among Women of Childbearing Age in 2021. A.** ASIR (age-standardized incidence rate); **B.** DALY (disability-adjusted life-year).


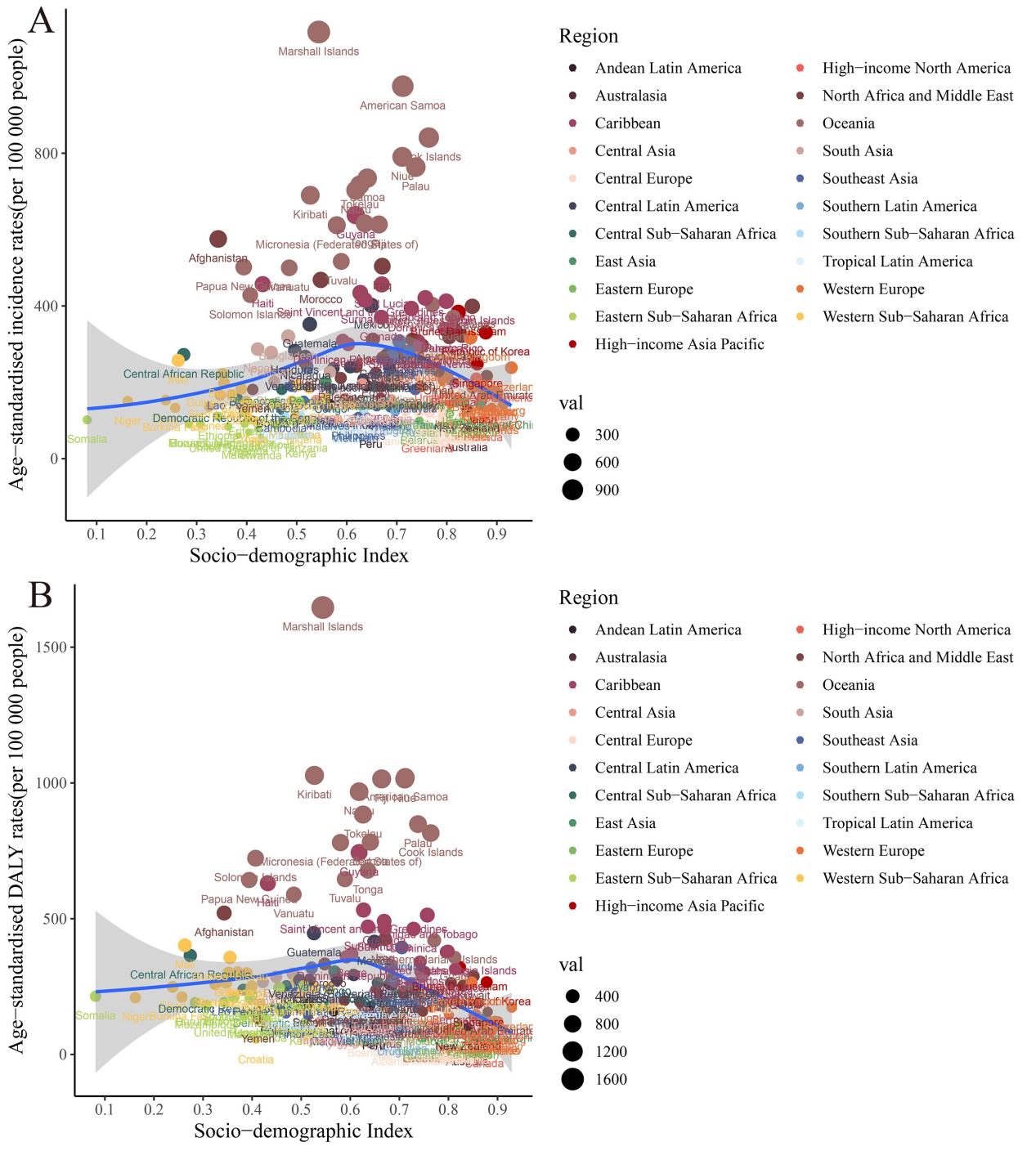

Supplement: S2 Fig — A. ASIR (age-standardized incidence rate); B. DALY (disability-adjusted life-year). (DOCX) [file pone.0322787.s002.docx]
