# Supplementary material for: Assessing the global burden of Type 2 diabetes in women of reproductive age
Source: PLoS One. 2025 Jul 14;20(7):e0322787. doi: 10.1371/journal.pone.0322787 (PMC12258576; doi:10.1371/journal.pone.0322787)
Supplement: S3 Fig — (DOCX) [file pone.0322787.s003.docx]

**S3 Fig. Global Heatmap of DALY (disability-adjusted life-year) of Type 2 Diabetes Mellitus Rates Among Women of Childbearing Age in 2021 by Country and Age Group.**

**
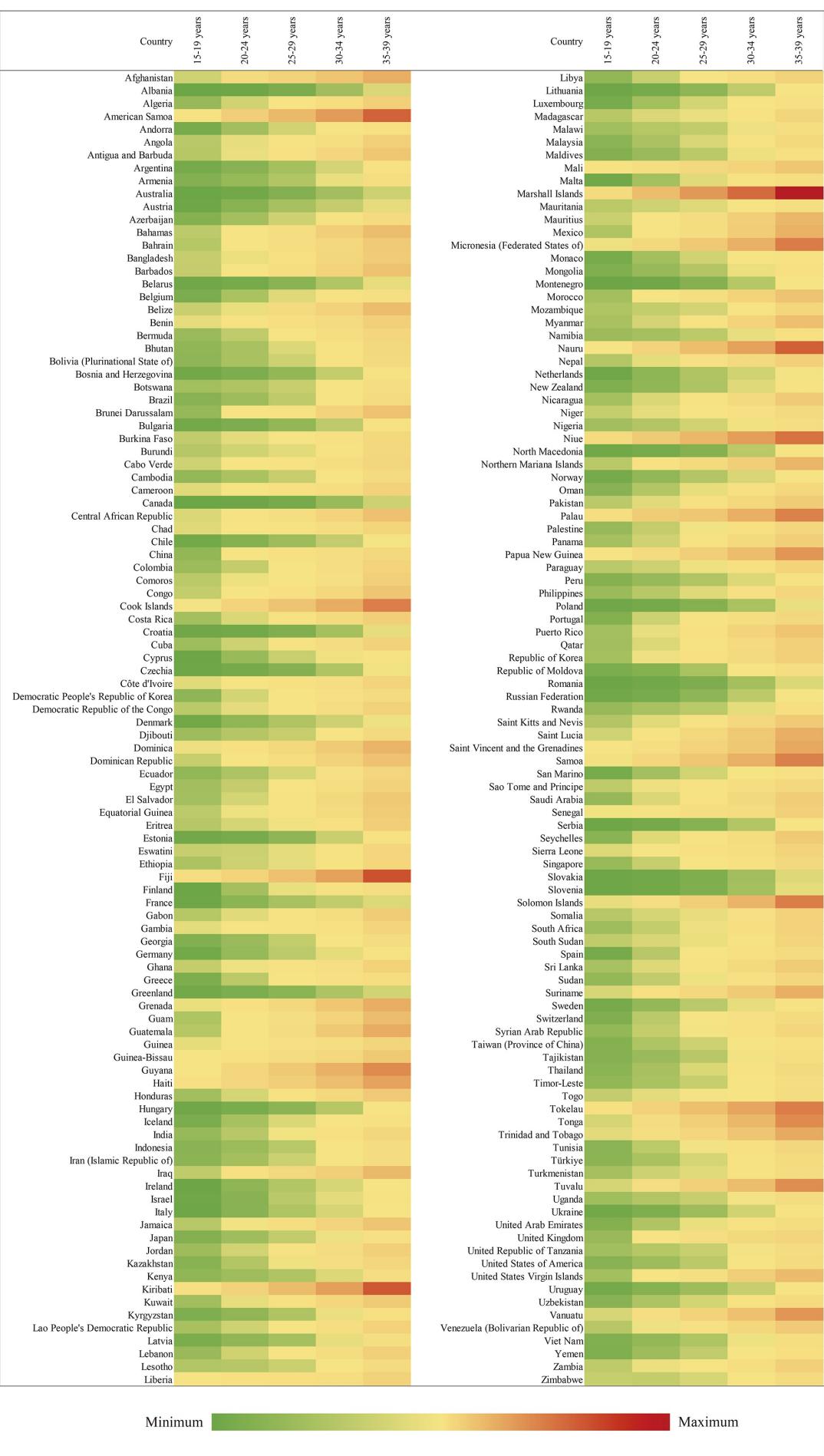
**
