# Supplementary material for: Assessing the global burden of Type 2 diabetes in women of reproductive age
Source: PLoS One. 2025 Jul 14;20(7):e0322787. doi: 10.1371/journal.pone.0322787 (PMC12258576; doi:10.1371/journal.pone.0322787)
Supplement: S1 Table — (DOCX) [file pone.0322787.s004.docx]

**Table S1. The incidence and incidence rate of type 2 diabetes mellitus burden in women of childbearing age in 1990 and 2021, and its temporal trends from 1990 to 2021.**

|  | Incidence No.(95%UI) |  |  | ASIR (per 100000) No.95%UI |  |  |
| --- | --- | --- | --- | --- | --- | --- |
| nation | 1990 | 2021 | 1990-2021 EAPC No.(95%CI) | 1990 | 2021 | 1990-2021 EAPC No.(95%CI) |
| Afghanistan | 2987.53(2499.79,3547.00) | 33048.66(28053.63,38753.48) | 8.16(7.87,8.44) | 186.52(179.27,194.04) | 575.54(569.02,582.13) | 3.85(3.72,3.97) |
| Albania | 356.34(280.37,430.94) | 447.37(355.35,549.14) | 0.25(0.08,0.43) | 54.42(48.86,60.47) | 95.69(87.03,105.05) | 1.83(1.76,1.90) |
| Algeria | 5103.18(4264.76,6027.82) | 27607.44(22917.96,32848.89) | 5.42(5.10,5.74) | 114.05(110.86,117.32) | 312.00(308.29,315.75) | 3.13(3.04,3.22) |
| American Samoa | 36.20(30.84,42.85) | 83.31(70.90,98.74) | 2.30(1.84,2.76) | 371.77(259.25,519.87) | 975.64(776.00,1212.67) | 3.01(2.80,3.22) |
| Andorra | 8.06(6.39,9.73) | 21.13(17.46,25.21) | 2.71(2.38,3.03) | 70.54(30.11,144.78) | 172.80(105.82,269.95) | 2.97(2.94,3.01) |
| Angola | 1632.26(1349.94,1967.18) | 11241.50(9498.21,13322.05) | 6.57(6.45,6.69) | 87.78(83.51,92.21) | 181.62(178.23,185.07) | 2.49(2.39,2.59) |
| Antigua and Barbuda | 23.43(19.09,27.73) | 55.55(44.89,67.95) | 2.54(2.25,2.84) | 179.26(113.99,269.50) | 307.62(231.75,402.16) | 1.66(1.61,1.72) |
| Argentina | 3903.14(3265.29,4696.04) | 12852.45(10259.27,15278.12) | 4.18(4.10,4.26) | 64.33(62.32,66.38) | 141.94(139.49,144.42) | 2.70(2.66,2.75) |
| Armenia | 640.78(508.95,781.32) | 939.65(768.09,1165.44) | 1.08(0.96,1.20) | 86.49(79.89,93.53) | 155.07(145.01,165.76) | 1.78(1.57,1.99) |
| Australia | 1514.95(1177.43,1851.97) | 3788.70(2939.57,4746.92) | 2.93(2.90,2.96) | 43.30(41.14,45.54) | 79.82(77.27,82.43) | 2.07(1.95,2.20) |
| Austria | 603.56(492.41,734.31) | 1459.72(1180.89,1746.08) | 2.80(2.70,2.90) | 40.72(37.52,44.15) | 106.55(101.06,112.28) | 3.12(3.03,3.21) |
| Azerbaijan | 1236.03(1021.93,1488.87) | 4540.40(3726.20,5535.98) | 4.18(4.02,4.35) | 78.93(74.54,83.54) | 204.97(198.94,211.17) | 3.17(3.05,3.30) |
| Bahamas | 100.19(81.36,119.95) | 270.89(219.44,324.80) | 3.02(2.85,3.20) | 172.64(140.29,210.58) | 339.44(300.20,382.46) | 2.14(2.07,2.22) |
| Bahrain | 119.90(92.13,146.45) | 735.76(592.79,900.16) | 6.55(6.11,6.99) | 123.62(102.18,148.46) | 296.53(275.44,318.89) | 2.81(2.72,2.89) |
| Bangladesh | 26253.30(22115.63,30427.85) | 114405.77(96514.92,134087.74) | 4.86(4.74,4.97) | 132.09(130.45,133.74) | 320.13(318.28,321.99) | 2.93(2.80,3.06) |
| Barbados | 87.81(73.62,103.94) | 154.20(126.40,189.37) | 1.55(1.39,1.72) | 157.45(126.22,194.28) | 297.38(252.07,348.93) | 1.84(1.74,1.94) |
| Belarus | 1126.37(915.12,1360.26) | 1673.86(1372.34,1997.02) | 0.99(0.86,1.13) | 54.18(51.04,57.47) | 98.63(93.80,103.69) | 1.61(1.47,1.75) |
| Belgium | 1405.62(1130.70,1690.46) | 3200.05(2652.44,3776.76) | 2.67(2.58,2.75) | 77.20(73.19,81.38) | 184.85(178.44,191.44) | 2.78(2.73,2.82) |
| Belize | 49.49(41.01,58.98) | 279.75(232.20,333.93) | 5.76(5.57,5.96) | 149.21(109.54,199.85) | 299.68(265.49,337.16) | 2.22(2.09,2.36) |
| Benin | 751.04(624.30,922.00) | 6022.77(5091.52,7207.97) | 6.89(6.71,7.07) | 82.79(76.89,89.06) | 221.11(215.45,226.90) | 3.14(2.97,3.32) |
| Bermuda | 13.28(10.73,16.01) | 18.61(15.10,22.30) | 0.87(0.75,0.99) | 97.02(51.38,172.81) | 198.01(116.84,319.25) | 2.20(2.11,2.28) |
| Bhutan | 124.75(102.68,147.16) | 334.89(276.18,391.05) | 3.57(3.41,3.74) | 111.70(92.45,134.08) | 201.95(180.88,224.85) | 1.92(1.81,2.03) |
| Bolivia (Plurinational State of) | 900.99(730.41,1081.00) | 3448.95(2859.06,4149.91) | 4.43(4.32,4.55) | 76.97(71.99,82.22) | 143.04(138.30,147.90) | 1.95(1.90,2.01) |
| Bosnia and Herzegovina | 573.97(454.50,719.02) | 683.86(541.13,836.44) | 0.80(0.62,0.98) | 61.01(56.12,66.22) | 123.76(114.61,133.53) | 2.35(2.21,2.48) |
| Botswana | 146.02(118.93,177.29) | 650.21(535.05,781.29) | 4.93(4.78,5.07) | 56.64(47.63,66.99) | 119.72(110.67,129.32) | 2.45(2.41,2.49) |
| Brazil | 29754.92(24004.36,35718.39) | 64076.45(50405.98,76836.66) | 2.50(2.39,2.61) | 98.73(97.61,99.86) | 141.20(140.11,142.31) | 1.14(1.05,1.22) |
| Brunei Darussalam | 74.82(63.56,87.00) | 369.76(305.30,453.55) | 5.39(5.32,5.46) | 130.84(102.78,164.50) | 383.61(345.19,425.49) | 3.66(3.47,3.85) |
| Bulgaria | 1135.91(903.14,1383.44) | 1324.93(1055.90,1626.48) | 0.72(0.56,0.88) | 72.79(68.60,77.17) | 119.49(113.02,126.28) | 1.61(1.54,1.68) |
| Burkina Faso | 901.69(740.72,1052.00) | 6110.74(5142.02,7228.05) | 6.43(6.33,6.53) | 53.91(50.39,57.63) | 133.14(129.77,136.58) | 2.97(2.87,3.07) |
| Burundi | 572.22(470.13,683.44) | 2348.66(1916.70,2800.89) | 4.77(4.55,4.98) | 55.82(51.28,60.68) | 90.73(87.05,94.53) | 1.42(1.34,1.50) |
| Cabo Verde | 58.03(47.58,70.06) | 222.24(185.09,265.99) | 4.55(4.15,4.96) | 85.60(64.41,112.45) | 185.47(161.86,211.62) | 2.60(2.39,2.81) |
| Cambodia | 1192.62(990.52,1409.46) | 4717.84(3992.82,5514.62) | 4.33(4.24,4.42) | 60.70(57.27,64.29) | 130.06(126.37,133.83) | 2.31(2.24,2.39) |
| Cameroon | 1240.32(1028.77,1478.49) | 9888.34(8261.60,11894.06) | 7.04(6.84,7.25) | 64.33(60.72,68.12) | 151.81(148.80,154.87) | 2.89(2.75,3.04) |
| Canada | 1540.70(1065.92,2024.13) | 7035.19(5231.36,8770.68) | 4.28(3.98,4.59) | 25.41(24.15,26.73) | 106.78(104.28,109.33) | 4.30(4.14,4.46) |
| Central African Republic | 606.01(501.15,730.96) | 3037.30(2550.00,3587.04) | 5.44(5.19,5.68) | 118.52(109.08,128.63) | 272.53(262.79,282.56) | 2.81(2.68,2.95) |
| Chad | 742.62(599.26,887.01) | 5011.86(4157.82,6080.19) | 6.34(6.23,6.44) | 68.17(63.28,73.36) | 153.28(148.93,157.74) | 2.64(2.50,2.78) |
| Chile | 1804.72(1450.96,2208.29) | 4866.57(3886.47,6020.43) | 3.10(2.91,3.29) | 64.01(61.08,67.04) | 133.76(130.01,137.60) | 2.43(2.28,2.59) |
| China | 325574.03(258977.72,400411.38) | 517925.05(431841.94,610747.91) | 1.79(1.34,2.25) | 121.27(120.85,121.69) | 266.01(265.27,266.76) | 2.66(2.36,2.97) |
| Colombia | 9591.87(8215.94,11404.05) | 20872.10(17619.50,24992.18) | 2.03(1.83,2.23) | 138.29(135.52,141.12) | 207.67(204.86,210.52) | 1.00(0.79,1.22) |
| Comoros | 56.14(46.31,67.66) | 220.67(185.92,263.21) | 4.63(4.45,4.80) | 67.00(50.20,88.07) | 145.04(126.48,165.66) | 2.66(2.53,2.79) |
| Congo | 386.84(319.88,466.47) | 2025.07(1664.94,2403.74) | 5.46(5.24,5.68) | 85.83(77.28,95.14) | 180.68(172.87,188.75) | 2.36(2.25,2.47) |
| Cook Islands | 15.56(13.26,18.07) | 26.48(22.81,31.40) | 1.43(1.14,1.71) | 433.27(243.75,718.15) | 841.13(551.48,1232.23) | 2.05(1.88,2.22) |
| Costa Rica | 856.49(713.97,1006.72) | 2726.91(2235.84,3235.13) | 3.61(3.39,3.83) | 136.05(127.04,145.57) | 272.12(261.95,282.62) | 2.15(2.07,2.22) |
| Croatia | 578.21(448.32,716.31) | 713.08(548.91,871.59) | 0.70(0.64,0.77) | 59.27(54.52,64.35) | 101.66(94.28,109.55) | 1.78(1.72,1.83) |
| Cuba | 3167.25(2643.49,3753.07) | 4274.26(3559.25,5015.24) | 0.32(-0.03,0.67) | 135.81(131.06,140.68) | 237.53(230.40,244.84) | 1.62(1.50,1.74) |
| Cyprus | 122.88(99.23,147.79) | 382.89(294.79,467.67) | 3.99(3.86,4.11) | 82.37(68.43,98.41) | 158.88(142.59,176.78) | 2.06(2.03,2.09) |
| Czechia | 1205.31(942.94,1512.39) | 1886.64(1513.67,2320.77) | 1.98(1.69,2.28) | 62.09(58.61,65.75) | 109.24(104.29,114.39) | 1.83(1.76,1.89) |
| Cote d'Ivoire | 1695.62(1404.21,2066.90) | 9236.04(7515.41,10976.79) | 5.45(5.18,5.72) | 75.24(71.60,79.03) | 167.19(163.78,170.65) | 2.61(2.52,2.70) |
| Democratic People's Republic of Korea | 4366.08(3610.70,5113.10) | 9453.81(7817.15,11020.36) | 2.44(2.36,2.53) | 98.20(95.28,101.19) | 201.47(197.41,205.59) | 2.26(2.23,2.28) |
| Democratic Republic of the Congo | 5054.41(4144.87,6044.29) | 27178.94(22458.22,32029.45) | 5.36(5.28,5.45) | 73.28(71.24,75.37) | 156.97(155.08,158.88) | 2.30(2.25,2.35) |
| Denmark | 519.04(415.76,632.39) | 1196.40(988.09,1441.05) | 2.61(2.44,2.77) | 55.55(50.86,60.57) | 133.33(125.86,141.16) | 2.82(2.76,2.89) |
| Djibouti | 33.33(27.37,40.06) | 220.12(182.30,259.10) | 6.32(6.09,6.54) | 43.11(29.34,61.75) | 86.51(75.44,98.80) | 2.24(2.19,2.29) |
| Dominica | 27.25(22.78,32.13) | 50.00(41.36,60.59) | 1.79(1.65,1.93) | 205.75(134.73,303.40) | 393.68(292.15,519.56) | 2.08(1.97,2.18) |
| Dominican Republic | 2017.74(1701.67,2390.99) | 6929.25(5693.20,8307.55) | 4.01(3.89,4.12) | 134.89(128.96,141.04) | 309.19(301.94,316.56) | 2.77(2.69,2.85) |
| Ecuador | 1648.71(1354.39,1986.76) | 6923.05(5833.53,8180.94) | 4.94(4.81,5.08) | 85.25(81.13,89.52) | 191.42(186.93,195.99) | 2.90(2.75,3.06) |
| Egypt | 6070.95(4848.70,7199.70) | 45668.54(37209.30,54386.07) | 6.88(6.78,6.98) | 60.55(59.03,62.11) | 223.93(221.88,226.00) | 4.41(4.32,4.50) |
| El Salvador | 1148.96(965.06,1338.00) | 3178.70(2692.69,3817.26) | 3.08(2.96,3.20) | 112.56(106.02,119.43) | 236.45(228.27,244.85) | 2.19(2.07,2.31) |
| Equatorial Guinea | 68.41(56.28,80.64) | 574.76(471.13,686.31) | 7.24(7.13,7.36) | 86.57(67.03,110.26) | 193.36(177.76,210.02) | 2.64(2.63,2.66) |
| Eritrea | 355.41(288.68,424.00) | 1602.25(1327.11,1898.72) | 5.37(5.22,5.52) | 57.23(51.33,63.66) | 121.56(115.64,127.71) | 2.41(2.38,2.44) |
| Estonia | 192.41(154.08,229.33) | 327.75(261.86,400.13) | 1.76(1.67,1.84) | 64.07(55.29,73.93) | 149.01(133.00,166.67) | 2.63(2.50,2.77) |
| Eswatini | 109.13(89.40,129.68) | 343.22(282.18,406.08) | 3.53(3.15,3.91) | 71.05(57.98,86.46) | 133.98(120.14,149.03) | 2.04(1.81,2.27) |
| Ethiopia | 7677.48(6415.45,8985.71) | 25249.07(20691.50,29745.96) | 3.57(3.45,3.70) | 82.73(80.86,84.63) | 110.67(109.28,112.06) | 0.67(0.56,0.79) |
| Fiji | 438.37(364.14,519.22) | 1077.60(931.46,1253.46) | 3.02(2.95,3.09) | 283.11(257.09,311.16) | 613.42(577.32,651.19) | 2.60(2.48,2.71) |
| Finland | 927.02(746.19,1110.75) | 2028.97(1651.56,2407.76) | 2.54(2.32,2.75) | 103.09(96.48,110.07) | 251.56(240.63,262.88) | 2.79(2.64,2.94) |
| France | 5137.87(4176.99,6102.56) | 11386.49(9315.81,13555.73) | 3.09(2.92,3.27) | 46.89(45.61,48.19) | 115.32(113.20,117.46) | 3.45(3.28,3.62) |
| Gabon | 160.09(129.01,192.62) | 736.33(610.70,885.17) | 4.94(4.83,5.05) | 89.12(75.54,104.61) | 189.46(175.90,203.83) | 2.38(2.29,2.47) |
| Gambia | 123.46(100.27,147.54) | 775.61(639.68,928.41) | 6.02(5.91,6.13) | 65.32(54.01,78.56) | 150.43(139.85,161.65) | 2.78(2.70,2.86) |
| Georgia | 790.87(640.23,960.16) | 1199.84(983.99,1486.98) | 1.19(1.01,1.36) | 71.37(66.47,76.56) | 198.46(187.18,210.33) | 3.37(3.19,3.56) |
| Germany | 8028.69(6576.70,9422.28) | 19530.03(16194.28,22975.37) | 2.70(2.49,2.91) | 54.75(53.53,55.98) | 161.16(158.87,163.48) | 3.52(3.42,3.62) |
| Ghana | 2357.48(1919.70,2775.28) | 11601.99(9372.79,13828.35) | 5.52(5.36,5.67) | 81.91(78.59,85.35) | 157.23(154.38,160.13) | 2.24(2.10,2.37) |
| Greece | 1725.33(1409.87,2072.79) | 2745.20(2280.24,3256.14) | 1.31(1.01,1.62) | 92.36(88.05,96.83) | 204.53(196.84,212.45) | 2.59(2.52,2.65) |
| Greenland | 1.82(1.24,2.48) | 8.04(6.34,9.71) | 4.16(3.74,4.58) | 15.49(1.53,70.56) | 76.85(33.11,156.10) | 5.37(5.27,5.47) |
| Grenada | 33.71(28.17,40.48) | 70.74(57.36,85.55) | 2.22(1.84,2.60) | 212.04(146.05,299.38) | 370.48(288.91,468.61) | 1.74(1.68,1.79) |
| Guam | 53.65(44.35,63.23) | 98.02(84.44,113.70) | 1.71(1.54,1.87) | 188.48(141.37,246.58) | 370.62(300.88,451.86) | 2.09(2.03,2.16) |
| Guatemala | 1976.19(1678.37,2346.23) | 11962.80(10079.37,14132.83) | 6.36(6.18,6.54) | 137.64(131.54,143.96) | 352.01(345.69,358.42) | 3.35(3.25,3.44) |
| Guinea | 703.68(583.84,837.32) | 3764.66(3146.23,4470.30) | 5.56(5.22,5.90) | 64.35(59.65,69.34) | 135.96(131.60,140.44) | 2.52(2.20,2.84) |
| Guinea-Bissau | 183.19(148.90,224.62) | 865.83(715.22,1038.44) | 5.18(5.08,5.28) | 95.47(81.92,110.78) | 197.82(184.77,211.59) | 2.32(2.24,2.41) |
| Guyana | 476.20(382.85,582.70) | 978.69(807.60,1172.34) | 2.29(2.23,2.36) | 296.39(269.99,324.81) | 637.95(598.32,679.62) | 2.67(2.53,2.80) |
| Haiti | 3063.94(2473.86,3648.78) | 12857.88(10514.46,15370.29) | 4.81(4.69,4.93) | 249.93(241.11,259.01) | 457.13(449.26,465.10) | 1.89(1.82,1.95) |
| Honduras | 1209.97(977.35,1446.12) | 6307.85(5200.59,7463.25) | 5.58(5.48,5.68) | 145.28(137.06,153.88) | 283.21(276.22,290.33) | 2.18(2.13,2.24) |
| Hungary | 1382.56(1113.17,1712.51) | 1699.35(1337.91,2096.58) | 1.20(0.89,1.52) | 68.24(64.64,72.00) | 110.15(104.94,115.58) | 1.59(1.47,1.71) |
| Iceland | 36.88(29.52,44.75) | 106.44(88.58,124.31) | 3.57(3.53,3.61) | 72.53(51.01,100.28) | 184.56(151.01,223.72) | 3.07(3.02,3.12) |
| India | 183955.46(149915.68,216957.06) | 669068.88(549072.83,783988.45) | 4.19(4.10,4.28) | 115.46(114.93,115.99) | 227.31(226.76,227.85) | 2.10(2.04,2.15) |
| Indonesia | 28923.33(23386.76,34281.81) | 78693.50(63987.93,93276.15) | 0.79(-0.13,1.72) | 78.01(77.10,78.92) | 137.33(136.38,138.30) | -0.53(-1.48,0.43) |
| Iran (Islamic Republic of) | 7190.30(5793.40,8670.82) | 36281.83(28263.30,44217.42) | 4.98(4.70,5.25) | 74.36(72.61,76.13) | 189.48(187.47,191.51) | 2.55(2.37,2.73) |
| Iraq | 7201.51(6085.11,8396.36) | 41161.57(34002.73,48944.30) | 5.99(5.74,6.24) | 219.01(213.84,224.29) | 504.23(499.34,509.15) | 2.93(2.76,3.09) |
| Ireland | 512.48(420.57,610.70) | 1165.10(958.25,1396.21) | 3.00(2.82,3.17) | 75.62(69.20,82.48) | 147.62(139.17,156.47) | 2.24(2.16,2.32) |
| Israel | 763.33(613.23,941.94) | 2301.01(1863.74,2789.53) | 3.61(3.46,3.76) | 79.71(74.14,85.59) | 141.00(135.29,146.88) | 1.85(1.80,1.90) |
| Italy | 9148.09(6773.26,11591.62) | 10754.78(7962.66,13734.92) | 0.36(0.06,0.66) | 86.00(84.25,87.79) | 138.65(136.02,141.33) | 1.46(1.24,1.68) |
| Jamaica | 531.75(445.74,612.68) | 1504.44(1275.26,1771.11) | 3.27(3.08,3.47) | 114.21(104.55,124.59) | 247.80(235.41,260.70) | 2.56(2.45,2.68) |
| Japan | 18353.21(14824.18,22068.61) | 24906.95(20021.70,29966.29) | 0.82(0.67,0.97) | 82.38(81.19,83.59) | 152.93(151.01,154.87) | 1.89(1.84,1.94) |
| Jordan | 934.91(763.06,1088.60) | 7317.26(6026.44,8622.46) | 7.09(7.01,7.17) | 152.73(142.66,163.37) | 311.93(304.79,319.19) | 2.49(2.35,2.64) |
| Kazakhstan | 3387.81(2790.70,4130.18) | 9377.58(7889.68,10845.77) | 3.71(3.56,3.86) | 99.58(96.25,103.00) | 257.55(252.29,262.91) | 3.27(3.15,3.39) |
| Kenya | 1843.13(1504.36,2195.62) | 6745.98(5534.40,7930.78) | 4.07(3.94,4.21) | 43.55(41.50,45.68) | 63.83(62.30,65.40) | 1.09(1.03,1.16) |
| Kiribati | 55.17(47.46,64.79) | 173.40(143.89,204.51) | 3.92(3.80,4.04) | 368.43(276.86,482.15) | 689.96(591.00,801.00) | 2.14(2.02,2.26) |
| Kuwait | 588.96(461.86,730.43) | 4740.57(3834.51,5834.58) | 7.64(7.13,8.14) | 169.40(155.92,183.76) | 399.04(386.81,411.64) | 2.73(2.62,2.83) |
| Kyrgyzstan | 566.90(452.56,662.80) | 2189.61(1832.32,2640.10) | 4.41(4.30,4.51) | 64.94(59.67,70.57) | 158.39(151.80,165.19) | 2.96(2.82,3.11) |
| Lao People's Democratic Republic | 647.10(541.15,760.08) | 2968.47(2451.30,3494.19) | 4.82(4.71,4.94) | 86.84(80.18,93.94) | 189.90(183.12,196.87) | 2.35(2.27,2.43) |
| Latvia | 319.70(253.07,390.09) | 449.50(367.92,540.58) | 0.91(0.77,1.04) | 64.01(57.16,71.50) | 148.57(134.84,163.51) | 2.58(2.48,2.69) |
| Lebanon | 747.44(602.06,913.34) | 3235.52(2636.57,3907.55) | 5.22(4.97,5.47) | 129.01(119.88,138.69) | 275.89(266.23,285.84) | 2.57(2.51,2.63) |
| Lesotho | 132.61(106.07,160.27) | 454.80(375.55,536.92) | 4.06(3.96,4.15) | 44.96(37.57,53.43) | 112.95(102.72,123.97) | 3.07(3.02,3.13) |
| Liberia | 340.36(277.83,407.20) | 1892.78(1602.98,2264.41) | 6.79(6.43,7.16) | 72.86(65.23,81.18) | 167.16(159.65,174.96) | 2.79(2.68,2.91) |
| Libya | 701.80(569.89,838.40) | 4312.06(3474.58,5205.80) | 6.48(5.89,7.07) | 103.61(95.77,111.97) | 285.52(277.03,294.22) | 3.61(3.38,3.84) |
| Lithuania | 403.72(320.00,496.95) | 561.17(457.17,663.66) | 0.53(0.30,0.76) | 56.12(50.77,61.90) | 128.31(117.86,139.57) | 2.52(2.42,2.63) |
| Luxembourg | 55.49(44.05,67.57) | 190.31(155.07,228.31) | 4.04(3.86,4.22) | 76.53(57.38,100.83) | 176.78(151.98,204.92) | 2.74(2.71,2.76) |
| Madagascar | 1129.26(953.51,1343.50) | 5319.78(4458.14,6254.28) | 4.98(4.93,5.04) | 51.53(48.50,54.71) | 92.05(89.55,94.60) | 1.77(1.72,1.81) |
| Malawi | 718.36(595.52,854.29) | 2477.23(2071.98,2891.39) | 3.85(3.57,4.14) | 39.99(37.04,43.14) | 60.33(57.92,62.82) | 1.11(0.98,1.24) |
| Malaysia | 3789.72(3052.30,4599.35) | 12101.32(9917.34,14375.80) | 3.74(3.33,4.15) | 106.40(103.02,109.85) | 181.37(178.14,184.63) | 1.75(1.47,2.03) |
| Maldives | 28.48(23.36,33.85) | 128.86(102.70,154.73) | 4.85(4.77,4.93) | 80.08(52.32,118.99) | 136.05(113.07,162.88) | 1.51(1.33,1.69) |
| Mali | 1971.27(1599.25,2402.90) | 12189.78(10118.17,14808.61) | 6.45(6.24,6.67) | 125.36(119.82,131.12) | 257.30(252.62,262.05) | 2.55(2.29,2.80) |
| Malta | 47.69(38.17,57.70) | 141.87(115.63,169.48) | 3.89(3.77,4.00) | 70.30(51.62,93.87) | 219.38(183.92,260.44) | 3.86(3.82,3.90) |
| Marshall Islands | 35.27(29.65,41.71) | 128.97(109.94,152.78) | 4.39(4.09,4.68) | 437.78(303.42,614.93) | 1117.56(932.70,1329.12) | 3.32(3.13,3.50) |
| Mauritania | 230.84(184.88,273.88) | 957.09(831.21,1104.01) | 4.31(4.19,4.42) | 60.27(52.64,68.76) | 112.68(105.54,120.22) | 1.71(1.60,1.82) |
| Mauritius | 302.50(248.35,358.02) | 705.63(589.97,839.59) | 2.94(2.69,3.19) | 124.71(111.03,139.64) | 307.10(284.83,330.71) | 3.19(3.05,3.33) |
| Mexico | 47598.82(39169.26,55783.19) | 106075.73(87870.69,123354.93) | 2.54(2.17,2.91) | 281.02(278.46,283.60) | 401.41(399.00,403.83) | 1.20(0.95,1.44) |
| Micronesia (Federated States of) | 53.26(46.01,61.39) | 123.68(108.14,142.07) | 2.82(2.45,3.19) | 281.53(210.05,370.93) | 611.46(507.66,731.13) | 2.79(2.48,3.11) |
| Monaco | 3.07(2.42,3.67) | 7.90(6.57,9.41) | 3.16(3.08,3.24) | 67.12(13.39,225.73) | 171.08(72.76,346.07) | 3.09(3.06,3.13) |
| Mongolia | 247.32(202.51,288.75) | 999.60(833.32,1201.45) | 4.56(4.45,4.67) | 60.53(53.02,68.92) | 151.50(142.16,161.34) | 3.05(2.98,3.12) |
| Montenegro | 94.99(75.17,115.10) | 144.25(115.81,174.39) | 1.42(1.31,1.52) | 76.92(62.23,94.08) | 130.91(110.31,154.54) | 1.69(1.59,1.80) |
| Morocco | 6801.64(5512.04,8137.77) | 34619.30(27950.41,41887.59) | 5.51(5.27,5.75) | 140.23(136.88,143.65) | 468.21(463.29,473.18) | 4.10(3.97,4.23) |
| Mozambique | 995.14(816.21,1198.30) | 5525.05(4603.43,6648.46) | 5.64(5.54,5.75) | 39.79(37.33,42.38) | 90.82(88.37,93.32) | 2.70(2.65,2.76) |
| Myanmar | 10947.22(9010.79,12974.97) | 27181.58(23590.38,30703.65) | 2.32(2.05,2.60) | 134.36(131.83,136.93) | 239.96(237.11,242.83) | 1.36(1.19,1.53) |
| Namibia | 163.31(133.31,194.86) | 540.61(441.95,639.63) | 3.71(3.51,3.90) | 60.49(51.34,70.94) | 102.76(94.26,111.86) | 1.60(1.52,1.68) |
| Nauru | 6.55(5.47,7.79) | 15.94(13.58,18.43) | 2.49(2.31,2.67) | 334.83(129.03,718.94) | 703.09(400.56,1151.73) | 2.16(2.02,2.29) |
| Nepal | 4640.38(3840.73,5536.79) | 20607.77(17522.49,23836.03) | 4.93(4.60,5.26) | 128.31(124.60,132.11) | 287.01(283.09,290.97) | 2.54(2.20,2.88) |
| Netherlands | 1502.06(1150.89,1865.66) | 3258.66(2606.39,3950.36) | 2.33(2.19,2.47) | 50.44(47.91,53.07) | 124.85(120.59,129.23) | 2.91(2.83,2.98) |
| New Zealand | 483.91(365.04,607.70) | 1190.35(1038.89,1358.42) | 3.32(2.67,3.98) | 69.09(63.07,75.54) | 130.16(122.83,137.84) | 2.75(2.11,3.39) |
| Nicaragua | 1078.61(897.95,1257.47) | 3803.16(3173.77,4558.44) | 4.04(3.94,4.13) | 151.83(142.69,161.43) | 268.06(259.61,276.73) | 1.77(1.70,1.83) |
| Niger | 1041.70(869.34,1239.40) | 6809.00(5594.94,8111.46) | 6.03(5.95,6.12) | 73.99(69.44,78.79) | 150.42(146.69,154.24) | 2.28(2.23,2.34) |
| Nigeria | 10295.88(8471.70,12269.36) | 46784.22(38325.83,55399.64) | 4.92(4.78,5.06) | 62.53(61.29,63.80) | 100.53(99.60,101.47) | 1.56(1.48,1.64) |
| Niue | 1.24(1.05,1.46) | 2.19(1.88,2.60) | 1.58(1.34,1.83) | 330.80(15.18,1640.19) | 790.55(109.90,2750.72) | 2.91(2.82,3.01) |
| North Macedonia | 262.58(197.34,341.75) | 529.79(408.94,671.86) | 2.38(2.30,2.47) | 66.77(58.94,75.37) | 121.93(111.71,133.00) | 1.93(1.83,2.03) |
| Northern Mariana Islands | 23.60(19.92,27.81) | 32.26(27.11,38.21) | -0.33(-1.37,0.73) | 202.41(128.80,304.80) | 403.68(276.08,573.14) | 2.09(2.02,2.16) |
| Norway | 764.48(602.87,926.65) | 1235.46(955.76,1513.23) | 1.44(1.27,1.61) | 96.96(90.20,104.11) | 141.62(133.78,149.83) | 1.11(1.01,1.21) |
| Oman | 309.71(248.73,382.05) | 1914.61(1505.88,2337.82) | 5.27(4.84,5.71) | 115.12(102.56,128.85) | 229.02(218.66,239.79) | 1.36(0.93,1.79) |
| Pakistan | 22238.81(18107.17,26301.80) | 132767.55(109434.29,158280.70) | 6.28(6.05,6.52) | 125.83(124.15,127.53) | 278.26(276.76,279.77) | 2.78(2.56,2.99) |
| Palau | 11.52(9.76,13.59) | 18.64(16.03,21.43) | 1.12(0.71,1.53) | 351.69(178.63,625.58) | 763.80(455.49,1212.50) | 2.56(2.50,2.62) |
| Palestine | 324.81(267.72,383.57) | 2137.40(1809.65,2521.63) | 6.29(6.16,6.43) | 98.68(87.84,110.59) | 211.39(202.42,220.67) | 2.42(2.35,2.50) |
| Panama | 597.69(496.46,713.14) | 1904.14(1574.15,2297.27) | 3.63(3.57,3.70) | 123.93(114.10,134.43) | 235.13(224.68,245.95) | 2.05(1.99,2.10) |
| Papua New Guinea | 1604.00(1339.45,1871.41) | 10377.28(8829.60,12128.03) | 6.14(6.10,6.19) | 207.92(197.71,218.54) | 501.82(492.19,511.59) | 2.77(2.73,2.81) |
| Paraguay | 667.44(542.36,802.62) | 2339.17(1927.07,2824.58) | 4.20(4.11,4.30) | 91.79(84.91,99.11) | 156.73(150.44,163.22) | 1.80(1.71,1.89) |
| Peru | 2280.63(1822.11,2720.44) | 6618.94(5492.37,7825.85) | 3.54(3.28,3.80) | 54.25(52.02,56.56) | 88.88(86.75,91.06) | 1.74(1.47,2.01) |
| Philippines | 8704.13(6919.68,10432.96) | 24812.10(20264.20,29169.22) | 3.39(3.10,3.68) | 71.53(70.01,73.07) | 110.37(109.00,111.76) | 1.41(1.10,1.73) |
| Poland | 6476.28(5047.36,7898.99) | 7656.59(5942.35,9504.63) | 0.84(0.57,1.12) | 79.32(77.38,81.30) | 104.27(101.90,106.70) | 0.70(0.61,0.79) |
| Portugal | 1991.99(1626.90,2425.38) | 3862.91(3116.90,4666.09) | 2.14(1.95,2.33) | 104.87(100.31,109.58) | 263.53(255.21,272.07) | 3.10(3.04,3.16) |
| Puerto Rico | 1221.08(1001.08,1495.43) | 1829.22(1506.12,2264.07) | 1.49(1.32,1.67) | 168.45(159.13,178.18) | 339.27(323.86,355.25) | 2.49(2.38,2.61) |
| Qatar | 99.37(80.77,123.01) | 1537.32(1188.53,1954.44) | 9.80(9.34,10.26) | 141.34(114.64,172.97) | 321.72(304.39,339.99) | 2.70(2.64,2.75) |
| Republic of Korea | 11111.62(9449.63,13014.36) | 25612.97(21510.50,29259.43) | 3.15(2.79,3.51) | 109.52(107.49,111.59) | 330.80(326.66,334.98) | 4.09(3.65,4.53) |
| Republic of Moldova | 773.87(615.66,972.21) | 1390.28(1143.75,1687.39) | 1.93(1.84,2.02) | 81.72(76.02,87.76) | 190.14(179.98,200.86) | 2.66(2.47,2.85) |
| Romania | 2499.66(1970.08,3116.15) | 2701.74(2102.07,3356.03) | 0.38(0.09,0.68) | 57.21(54.97,59.52) | 91.01(87.57,94.55) | 1.36(1.30,1.42) |
| Russian Federation | 18207.03(14184.21,22084.46) | 32300.27(25264.14,39221.68) | 1.83(1.74,1.92) | 58.71(57.85,59.58) | 117.94(116.60,119.29) | 2.06(1.97,2.16) |
| Rwanda | 644.86(533.17,779.73) | 1670.21(1376.93,1959.92) | 3.00(2.69,3.31) | 48.38(44.66,52.34) | 59.01(56.19,61.93) | 0.15(-0.06,0.38) |
| Saint Kitts and Nevis | 16.98(14.15,20.22) | 35.17(28.32,42.62) | 2.17(2.06,2.27) | 203.53(118.02,330.32) | 298.08(207.45,417.42) | 0.99(0.89,1.09) |
| Saint Lucia | 69.57(57.50,84.60) | 154.72(131.93,181.23) | 2.67(2.40,2.94) | 263.85(204.36,336.49) | 456.41(387.02,535.51) | 1.92(1.81,2.03) |
| Saint Vincent and the Grenadines | 49.53(40.72,59.72) | 89.32(74.58,107.85) | 1.74(1.60,1.88) | 234.73(172.58,314.06) | 434.41(348.93,534.84) | 1.91(1.82,2.00) |
| Samoa | 86.24(73.62,101.83) | 282.68(239.92,335.74) | 3.85(3.66,4.04) | 295.37(234.68,368.16) | 734.87(651.03,827.04) | 3.05(2.84,3.26) |
| San Marino | 3.39(2.79,4.17) | 7.88(6.47,9.51) | 2.50(2.31,2.68) | 71.39(16.65,201.83) | 174.19(74.32,350.01) | 2.94(2.90,2.97) |
| Sao Tome and Principe | 17.25(14.11,20.67) | 88.61(74.91,104.01) | 5.37(5.30,5.44) | 81.05(46.40,134.45) | 191.31(153.22,236.45) | 2.80(2.77,2.83) |
| Saudi Arabia | 3131.64(2606.65,3659.36) | 26120.57(21401.94,31497.53) | 7.46(7.16,7.76) | 126.71(122.23,131.32) | 318.29(314.35,322.27) | 3.07(2.88,3.26) |
| Senegal | 1402.66(1134.31,1696.56) | 5871.79(4894.41,6913.03) | 4.98(4.74,5.21) | 98.90(93.65,104.39) | 182.89(178.19,187.69) | 2.33(2.18,2.48) |
| Serbia | 1517.79(1208.23,1905.11) | 1906.06(1475.72,2386.17) | 0.46(0.34,0.57) | 81.82(77.75,86.06) | 120.60(115.22,126.19) | 1.03(0.95,1.12) |
| Seychelles | 11.55(9.32,13.89) | 49.70(40.85,60.96) | 4.60(4.26,4.94) | 80.86(40.42,148.60) | 280.60(207.89,371.31) | 4.07(3.97,4.17) |
| Sierra Leone | 575.72(472.64,684.87) | 3174.01(2629.07,3845.86) | 6.09(5.90,6.28) | 69.19(63.59,75.20) | 166.48(160.63,172.49) | 2.93(2.83,3.04) |
| Singapore | 1071.34(854.55,1301.29) | 2506.53(2032.37,3069.15) | 2.86(2.56,3.15) | 142.12(133.69,150.98) | 247.91(237.44,258.81) | 1.55(1.29,1.81) |
| Slovakia | 617.58(478.73,774.87) | 958.16(738.13,1196.11) | 1.73(1.59,1.86) | 56.99(52.57,61.71) | 94.35(88.40,100.68) | 1.57(1.50,1.63) |
| Slovenia | 231.33(181.29,283.50) | 324.23(248.31,397.56) | 1.19(1.04,1.34) | 57.04(49.91,64.98) | 98.85(88.22,110.61) | 1.74(1.63,1.84) |
| Solomon Islands | 114.69(99.63,134.75) | 572.35(496.97,653.53) | 5.34(5.17,5.50) | 200.16(164.01,242.58) | 428.43(393.88,465.28) | 2.54(2.47,2.62) |
| Somalia | 744.26(616.27,895.40) | 3868.15(3202.22,4572.17) | 5.79(5.68,5.91) | 54.75(50.86,58.87) | 101.76(98.49,105.11) | 1.93(1.89,1.97) |
| South Africa | 8036.90(6548.56,9493.85) | 17985.24(14268.54,21386.55) | 2.36(2.12,2.59) | 103.41(101.13,105.73) | 148.00(145.84,150.19) | 0.99(0.90,1.08) |
| South Sudan | 451.05(369.50,536.87) | 1527.83(1280.64,1803.06) | 4.14(3.86,4.43) | 43.38(39.34,47.76) | 83.99(79.72,88.43) | 2.10(2.06,2.14) |
| Spain | 6091.56(4817.05,7332.88) | 14340.55(11860.41,16883.67) | 2.99(2.87,3.11) | 83.01(80.94,85.12) | 237.24(233.31,241.21) | 3.48(3.12,3.83) |
| Sri Lanka | 3738.62(3021.98,4597.43) | 11280.11(9285.22,13577.23) | 3.63(3.25,4.00) | 103.67(100.37,107.06) | 268.83(263.89,273.85) | 3.12(2.80,3.44) |
| Sudan | 3501.78(2873.33,4198.74) | 22640.33(19019.00,26097.09) | 6.06(5.94,6.19) | 97.48(94.23,100.83) | 253.49(250.18,256.83) | 3.03(2.92,3.15) |
| Suriname | 132.73(107.84,160.52) | 456.22(386.69,538.41) | 4.28(4.02,4.54) | 179.64(149.93,213.89) | 415.64(378.35,455.69) | 2.91(2.83,2.98) |
| Sweden | 1308.12(1016.00,1618.01) | 2545.78(2022.92,3120.39) | 2.08(1.87,2.28) | 89.98(85.16,95.02) | 160.16(153.92,166.60) | 1.76(1.61,1.91) |
| Switzerland | 1298.89(1025.24,1546.60) | 3212.43(2577.42,3800.75) | 3.16(2.93,3.38) | 101.43(95.92,107.20) | 238.24(229.88,246.86) | 2.92(2.75,3.09) |
| Syrian Arab Republic | 2620.84(2177.72,3099.94) | 6756.99(5614.32,8022.12) | 3.10(2.28,3.93) | 123.33(118.49,128.34) | 241.10(235.01,247.33) | 2.00(1.94,2.06) |
| Taiwan (Province of China) | 4439.09(3720.10,5233.12) | 5193.28(4133.95,6181.37) | 0.33(0.15,0.50) | 98.41(95.53,101.35) | 138.85(134.98,142.82) | 0.84(0.66,1.02) |
| Tajikistan | 647.19(541.43,770.13) | 3176.45(2650.76,3759.43) | 5.17(5.08,5.26) | 66.48(61.34,71.96) | 153.47(148.17,158.92) | 2.69(2.58,2.79) |
| Thailand | 8834.51(7265.84,10658.46) | 15342.51(12489.64,18496.45) | 1.57(1.26,1.89) | 69.67(68.22,71.14) | 134.42(132.28,136.59) | 2.07(1.97,2.18) |
| Timor-Leste | 79.82(65.60,95.45) | 391.75(324.46,462.80) | 5.12(4.87,5.36) | 54.26(42.91,67.86) | 151.14(136.11,167.51) | 3.38(3.34,3.43) |
| Togo | 372.72(306.95,445.52) | 1734.23(1441.92,2044.79) | 4.99(4.92,5.06) | 53.72(48.29,59.63) | 100.03(95.36,104.86) | 1.96(1.83,2.08) |
| Tokelau | 1.05(0.88,1.23) | 1.71(1.43,2.01) | 1.38(0.99,1.76) | 370.96(10.44,2168.20) | 716.76(66.58,2901.31) | 2.10(1.99,2.20) |
| Tonga | 51.64(43.22,60.85) | 120.25(103.15,139.76) | 2.80(2.68,2.93) | 301.04(222.52,400.34) | 616.28(510.47,738.22) | 2.40(2.34,2.46) |
| Trinidad and Tobago | 560.13(459.64,658.49) | 1143.57(944.99,1388.71) | 2.03(1.81,2.25) | 233.20(214.22,253.45) | 421.02(396.52,446.75) | 1.72(1.57,1.88) |
| Tunisia | 1558.79(1261.68,1864.98) | 6171.85(5004.17,7426.50) | 4.43(4.13,4.73) | 98.62(93.71,103.73) | 264.08(257.44,270.86) | 3.15(3.05,3.24) |
| Türkiye | 6905.39(5663.30,8138.77) | 30605.09(25641.25,35767.12) | 4.85(4.56,5.14) | 62.56(61.08,64.07) | 189.83(187.70,191.98) | 3.61(3.28,3.93) |
| Turkmenistan | 394.24(328.43,469.54) | 1421.78(1200.31,1670.29) | 4.06(3.87,4.25) | 53.99(48.73,59.69) | 146.75(139.21,154.58) | 3.28(3.14,3.42) |
| Tuvalu | 4.53(3.83,5.26) | 11.65(9.98,13.39) | 2.97(2.75,3.18) | 236.70(71.39,580.74) | 516.73(263.01,921.19) | 2.40(2.35,2.46) |
| Uganda | 1142.77(936.00,1350.63) | 5879.36(4893.53,6923.12) | 5.28(5.21,5.36) | 37.11(34.91,39.44) | 69.58(67.76,71.44) | 1.89(1.84,1.94) |
| Ukraine | 6924.83(5561.27,8463.21) | 10917.93(8638.32,13507.10) | 1.40(1.24,1.57) | 69.74(68.10,71.42) | 138.09(135.40,140.83) | 2.01(1.83,2.19) |
| United Arab Emirates | 297.01(230.89,365.50) | 2944.42(2259.03,3720.22) | 9.00(8.22,9.80) | 99.46(88.37,111.63) | 216.52(207.55,225.84) | 2.42(2.28,2.57) |
| United Kingdom | 10281.72(7977.05,12514.39) | 32828.81(28109.10,38339.53) | 3.88(3.71,4.05) | 97.16(95.28,99.06) | 317.31(313.85,320.80) | 3.79(3.59,3.98) |
| United Republic of Tanzania | 1744.49(1433.34,2091.27) | 9167.25(7738.22,10904.13) | 5.41(5.32,5.51) | 36.14(34.41,37.94) | 78.27(76.65,79.92) | 2.49(2.39,2.60) |
| United States of America | 38938.49(29981.01,47337.08) | 121339.57(103762.08,140065.32) | 3.74(3.59,3.89) | 72.00(71.28,72.72) | 209.82(208.63,211.00) | 3.60(3.42,3.79) |
| United States Virgin Islands | 45.09(35.74,55.08) | 50.83(41.31,61.33) | 0.65(0.22,1.08) | 213.99(155.99,287.15) | 412.48(306.22,546.01) | 2.33(2.16,2.51) |
| Uruguay | 264.07(206.88,325.47) | 768.80(619.00,939.38) | 3.76(3.56,3.97) | 46.31(40.89,52.25) | 124.05(115.42,133.18) | 3.48(3.30,3.65) |
| Uzbekistan | 2817.17(2352.73,3377.54) | 13956.49(11583.75,16646.50) | 5.35(5.15,5.54) | 69.25(66.69,71.89) | 195.44(192.19,198.73) | 3.46(3.34,3.58) |
| Vanuatu | 53.54(44.69,62.93) | 307.69(266.58,350.18) | 5.80(5.75,5.84) | 188.59(140.98,248.07) | 499.95(445.35,559.67) | 3.15(3.12,3.18) |
| Venezuela (Bolivarian Republic of) | 5072.35(4263.94,5937.41) | 12155.82(10200.71,14444.68) | 3.55(3.30,3.81) | 130.11(126.53,133.76) | 239.99(235.70,244.34) | 2.18(2.02,2.33) |
| Viet Nam | 8627.39(6957.94,10134.72) | 20992.94(17344.22,24228.94) | 2.53(2.38,2.69) | 62.40(61.08,63.75) | 104.84(103.41,106.29) | 1.32(1.06,1.59) |
| Yemen | 1780.33(1444.75,2118.83) | 12029.67(9939.81,14375.65) | 5.36(4.67,6.04) | 82.04(78.23,85.98) | 181.25(178.01,184.53) | 1.41(0.74,2.08) |
| Zambia | 1134.02(944.41,1377.08) | 6118.84(5156.59,7269.93) | 5.46(5.29,5.64) | 76.56(71.94,81.43) | 148.64(144.87,152.49) | 1.94(1.88,1.99) |
| Zimbabwe | 1106.39(892.91,1338.99) | 3579.71(2896.05,4242.09) | 3.66(3.55,3.78) | 56.14(52.78,59.67) | 110.18(106.57,113.88) | 2.16(2.08,2.24) |
