# Supplementary material for: Assessing the global burden of Type 2 diabetes in women of reproductive age
Source: PLoS One. 2025 Jul 14;20(7):e0322787. doi: 10.1371/journal.pone.0322787 (PMC12258576; doi:10.1371/journal.pone.0322787)
Supplement: S2 Table — (DOCX) [file pone.0322787.s005.docx]

**Table S2. The DALY and Age-standardized DALY rate of type 2 diabetes mellitus burden in women of childbearing age in 1990 and 2021, and its temporal trends from 1990 to 2021.**

| DALYs (Disability-Adjusted Life Years) | DALY No.(95%UI) |  |  | Age-standardized DALY rate (per 100000) No.95%UI |  |  |
| --- | --- | --- | --- | --- | --- | --- |
| nation | 1990 | 2021 | 1990-2021 EAPC No.(95%CI) | 1990 | 2021 | 1990-2021 EAPC No.(95%CI) |
| Afghanistan | 952.31(671.55,1348.78) | 6552.07(4618.26,9092.98) | 7.77(7.35,8.20) | 187.85(180.23,195.75) | 520.92(514.33,527.59) | 3.62(3.47,3.78) |
| Albania | 83.06(52.66,122.06) | 116.34(73.77,168.90) | -0.39(-0.62, -0.16) | 31.27(27.02,36.02) | 44.28(38.42,50.83) | 1.15(1.01,1.29) |
| Algeria | 1294.85(901.78,1788.00) | 9375.55(6624.71,12844.40) | 5.55(5.25,5.85) | 94.25(91.29,97.29) | 234.89(231.74,238.07) | 2.89(2.77,3.00) |
| American Samoa | 13.44(9.94,17.49) | 33.85(24.77,46.00) | 2.04(1.44,2.63) | 413.07(290.56,572.20) | 1019.77(811.20,1266.64) | 2.83(2.56,3.11) |
| Andorra | 2.40(1.62,3.34) | 7.54(4.85,10.73) | 2.47(2.07,2.87) | 59.43(24.34,127.61) | 123.00(71.27,203.56) | 2.41(2.38,2.44) |
| Angola | 1004.76(733.74,1342.35) | 4583.03(3253.28,6101.12) | 5.38(5.22,5.55) | 170.14(164.04,176.42) | 251.95(247.82,256.13) | 1.37(1.23,1.52) |
| Antigua and Barbuda | 11.62(9.04,14.72) | 23.87(16.79,33.29) | 1.43(1.15,1.71) | 262.63(181.54,368.54) | 304.98(230.25,398.16) | 0.49(0.38,0.61) |
| Argentina | 1640.20(1308.07,1989.49) | 3802.40(2705.93,5210.13) | 2.83(2.56,3.11) | 64.49(62.49,66.55) | 92.37(90.41,94.36) | 1.31(1.09,1.53) |
| Armenia | 207.20(145.58,283.39) | 351.85(241.34,493.84) | 0.60(0.34,0.86) | 79.91(73.56,86.68) | 116.24(107.79,125.29) | 1.33(1.13,1.52) |
| Australia | 510.12(373.01,697.36) | 1183.69(782.30,1674.55) | 2.30(2.16,2.44) | 29.73(27.96,31.58) | 44.72(42.88,46.62) | 1.41(1.35,1.48) |
| Austria | 193.72(136.62,267.75) | 466.07(303.67,688.64) | 2.03(1.83,2.24) | 36.24(33.26,39.43) | 74.63(70.35,79.13) | 2.32(2.25,2.39) |
| Azerbaijan | 329.63(232.63,462.23) | 1469.98(1004.32,2099.18) | 3.29(3.03,3.55) | 68.12(63.99,72.46) | 148.11(143.14,153.23) | 2.24(2.07,2.42) |
| Bahamas | 49.48(37.85,63.04) | 122.39(88.58,169.58) | 1.77(1.56,1.99) | 260.70(220.07,306.96) | 377.61(336.32,422.67) | 0.84(0.72,0.97) |
| Bahrain | 44.05(32.64,59.44) | 283.11(205.38,377.60) | 5.95(5.40,6.50) | 164.17(139.33,192.39) | 302.46(281.31,324.86) | 2.18(1.90,2.47) |
| Bangladesh | 8638.52(6278.24,11496.94) | 35677.62(25590.52,49941.24) | 3.88(3.78,3.98) | 181.01(179.04,183.00) | 296.71(294.91,298.52) | 1.81(1.62,2.00) |
| Barbados | 50.75(39.87,64.18) | 77.98(55.72,107.01) | 0.24(0.04,0.44) | 254.57(214.42,300.25) | 340.17(292.17,394.29) | 0.48(0.31,0.65) |
| Belarus | 326.83(212.69,473.62) | 606.16(410.84,870.91) | 0.62(0.36,0.89) | 33.62(31.19,36.19) | 57.59(54.12,61.28) | 1.22(1.03,1.41) |
| Belgium | 480.89(318.95,670.67) | 1060.93(691.99,1519.64) | 2.25(2.16,2.34) | 68.55(64.90,72.37) | 143.61(138.23,149.17) | 2.39(2.27,2.51) |
| Belize | 24.05(19.35,30.49) | 127.63(96.87,167.42) | 4.51(4.13,4.88) | 266.60(211.49,332.71) | 370.97(332.36,412.92) | 0.98(0.67,1.29) |
| Benin | 304.14(217.39,393.85) | 1840.85(1311.68,2531.00) | 5.93(5.76,6.10) | 136.69(128.97,144.79) | 300.09(293.23,307.08) | 2.39(2.25,2.54) |
| Bermuda | 6.48(4.65,8.81) | 9.17(6.18,12.97) | 0.26(0.16,0.36) | 120.91(70.11,200.84) | 199.07(120.29,316.58) | 1.51(1.40,1.63) |
| Bhutan | 36.06(25.98,50.83) | 106.68(74.38,151.14) | 2.68(2.53,2.83) | 116.31(96.21,139.63) | 166.70(147.63,187.61) | 0.89(0.75,1.03) |
| Bolivia (Plurinational State of) | 578.81(428.12,743.87) | 1493.06(1090.54,2005.14) | 2.36(2.28,2.44) | 149.24(142.24,156.52) | 158.32(153.33,163.44) | -0.09(-0.20,0.02) |
| Bosnia and Herzegovina | 184.87(127.35,250.34) | 235.66(158.64,334.86) | 0.20(-0.01,0.40) | 45.06(40.86,49.58) | 76.57(69.46,84.30) | 1.69(1.52,1.86) |
| Botswana | 64.63(44.85,87.80) | 308.35(220.13,419.25) | 5.02(4.62,5.42) | 84.85(73.49,97.57) | 144.98(135.09,155.43) | 2.32(1.93,2.71) |
| Brazil | 17082.61(14447.33,20092.76) | 30578.18(23326.38,38833.79) | 0.61(0.41,0.81) | 169.30(167.83,170.78) | 141.14(140.06,142.24) | -0.79(-1.03, -0.54) |
| Brunei Darussalam | 49.68(36.99,66.07) | 144.42(104.14,196.81) | 3.11(2.94,3.28) | 236.56(197.76,280.98) | 320.92(286.90,358.30) | 1.31(0.97,1.64) |
| Bulgaria | 413.30(293.29,581.97) | 481.18(331.92,668.93) | 0.07(-0.06,0.21) | 55.58(51.95,59.41) | 75.80(70.70,81.21) | 0.97(0.85,1.09) |
| Burkina Faso | 612.02(439.90,835.96) | 2455.45(1794.04,3378.02) | 4.86(4.74,4.98) | 131.88(126.23,137.72) | 211.44(207.06,215.88) | 1.51(1.40,1.62) |
| Burundi | 576.35(370.47,807.49) | 1482.54(1056.12,1995.84) | 2.88(2.59,3.17) | 185.05(176.53,193.90) | 191.25(185.77,196.85) | -0.30(-0.45, -0.15) |
| Cabo Verde | 16.59(11.71,22.35) | 92.52(66.45,125.30) | 4.06(3.41,4.71) | 123.37(96.82,155.67) | 246.27(219.18,275.86) | 1.95(1.41,2.48) |
| Cambodia | 735.82(539.26,1032.57) | 2171.15(1585.87,2940.46) | 2.38(2.17,2.59) | 121.46(116.55,126.54) | 150.79(146.83,154.83) | 0.39(0.24,0.54) |
| Cameroon | 723.29(510.05,970.29) | 4471.32(3198.04,6017.32) | 5.79(5.43,6.14) | 154.16(148.43,160.06) | 270.23(266.11,274.39) | 1.69(1.39,2.00) |
| Canada | 359.37(295.18,465.39) | 1623.98(1061.06,2411.43) | 2.61(2.01,3.22) | 13.62(12.71,14.59) | 37.62(36.18,39.11) | 2.53(2.07,3.00) |
| Central African Republic | 321.99(227.15,429.80) | 1245.64(890.28,1715.17) | 4.34(4.15,4.54) | 214.31(201.11,228.19) | 364.16(352.50,376.12) | 1.78(1.67,1.90) |
| Chad | 347.81(251.46,472.29) | 1757.24(1300.71,2330.82) | 5.97(5.73,6.21) | 119.08(112.47,126.00) | 248.77(243.02,254.64) | 2.38(2.11,2.65) |
| Chile | 552.32(416.51,725.99) | 1291.07(892.54,1814.62) | 2.19(2.09,2.30) | 50.69(48.07,53.43) | 78.84(76.01,81.76) | 1.49(1.40,1.58) |
| China | 85915.78(60579.33,119843.68) | 192978.88(126845.92,276878.39) | 2.61(2.08,3.14) | 103.13(102.74,103.53) | 228.74(228.13,229.36) | 2.77(2.39,3.15) |
| Colombia | 3812.45(2870.54,5021.34) | 8168.30(5797.54,11257.20) | 1.19(0.90,1.49) | 181.21(177.99,184.48) | 208.85(206.04,211.68) | 0.12(-0.20,0.44) |
| Comoros | 46.48(28.16,66.13) | 126.54(93.97,169.42) | 2.61(2.29,2.93) | 186.06(156.39,220.04) | 245.57(220.89,272.32) | 0.58(0.25,0.91) |
| Congo | 293.77(204.92,409.16) | 1299.99(922.58,1787.98) | 4.64(4.28,5.00) | 195.64(182.10,209.96) | 305.03(294.76,315.57) | 1.27(1.00,1.53) |
| Cook Islands | 8.40(6.26,11.29) | 9.98(7.32,13.37) | 0.66(0.43,0.89) | 584.12(353.62,911.68) | 815.41(527.50,1206.29) | 1.12(1.06,1.18) |
| Costa Rica | 313.10(229.73,431.45) | 1083.86(751.94,1514.85) | 3.36(3.22,3.50) | 147.07(137.54,157.10) | 259.18(249.46,269.22) | 1.79(1.73,1.86) |
| Croatia | 193.14(133.97,273.78) | 226.86(143.65,333.43) | 0.08(-0.04,0.20) | 38.63(34.82,42.77) | 52.35(47.16,58.03) | 1.12(1.04,1.21) |
| Cuba | 1238.89(921.84,1671.21) | 1729.67(1158.67,2460.08) | -0.38(-0.75, -0.01) | 167.71(162.34,173.21) | 236.37(229.42,243.51) | 0.81(0.62,1.01) |
| Cyprus | 45.76(32.94,61.22) | 144.61(95.86,209.90) | 3.27(3.15,3.39) | 75.89(62.72,91.10) | 108.92(97.21,122.05) | 0.78(0.59,0.97) |
| Czechia | 424.72(287.79,608.02) | 605.91(414.22,878.44) | 1.64(1.37,1.91) | 38.99(36.27,41.88) | 58.16(54.62,61.91) | 1.41(1.30,1.52) |
| Cote d'Ivoire | 638.43(477.11,849.40) | 3873.80(2741.07,5256.70) | 5.58(5.18,5.98) | 128.05(123.19,133.07) | 264.97(260.61,269.38) | 2.68(2.41,2.94) |
| Democratic People's Republic of Korea | 1218.91(812.46,1659.88) | 3170.21(2180.86,4380.57) | 2.12(1.92,2.32) | 102.61(99.53,105.76) | 186.21(182.43,190.05) | 1.84(1.82,1.87) |
| Democratic Republic of the Congo | 3476.61(2403.57,4765.60) | 12106.13(8704.87,16652.03) | 4.12(3.99,4.24) | 163.73(160.57,166.93) | 238.98(236.56,241.41) | 1.13(1.06,1.21) |
| Denmark | 146.00(99.36,201.12) | 269.21(166.61,387.34) | 1.91(1.74,2.08) | 39.84(35.96,44.06) | 82.97(77.20,89.09) | 2.28(2.13,2.43) |
| Djibouti | 24.04(16.34,33.85) | 171.63(118.55,246.54) | 5.74(5.38,6.10) | 102.14(79.37,129.83) | 156.70(141.82,172.77) | 1.33(1.10,1.57) |
| Dominica | 10.87(7.88,14.26) | 22.46(16.42,30.12) | 1.30(1.17,1.43) | 281.32(195.12,394.50) | 462.19(351.73,596.86) | 1.49(1.44,1.54) |
| Dominican Republic | 802.37(597.31,1082.40) | 2863.00(2079.14,3900.20) | 3.51(3.39,3.62) | 194.75(187.51,202.21) | 359.47(351.64,367.44) | 2.20(2.10,2.30) |
| Ecuador | 748.55(605.97,945.56) | 2652.15(1960.64,3486.21) | 3.29(2.99,3.59) | 114.29(109.48,119.27) | 178.16(173.83,182.58) | 1.23(0.98,1.48) |
| Egypt | 4052.94(3056.66,5246.52) | 17137.56(12785.09,22744.63) | 5.07(4.85,5.28) | 113.47(111.36,115.61) | 218.96(216.93,221.01) | 2.59(2.35,2.83) |
| El Salvador | 504.22(383.09,639.86) | 1400.30(1031.01,1865.67) | 2.60(2.42,2.78) | 156.89(148.93,165.18) | 274.47(265.59,283.59) | 1.57(1.43,1.71) |
| Equatorial Guinea | 48.97(35.11,69.95) | 268.79(190.13,375.27) | 5.89(5.74,6.04) | 180.28(150.88,213.90) | 278.45(259.21,298.78) | 1.31(1.21,1.41) |
| Eritrea | 357.28(252.50,505.43) | 1085.46(774.45,1524.59) | 4.48(4.24,4.73) | 156.26(146.13,166.94) | 230.88(222.53,239.48) | 1.52(1.40,1.64) |
| Estonia | 54.81(33.67,82.00) | 102.36(68.27,151.45) | 1.77(1.65,1.89) | 32.99(26.92,40.12) | 78.08(67.04,90.73) | 2.68(2.61,2.75) |
| Eswatini | 46.65(33.79,62.96) | 173.40(119.48,240.56) | 3.88(3.11,4.65) | 111.22(94.36,130.44) | 204.16(186.85,222.70) | 2.32(1.62,3.01) |
| Ethiopia | 8537.92(6523.41,10939.50) | 12698.97(9952.56,15612.45) | 0.75(0.46,1.05) | 288.98(285.38,292.61) | 191.85(189.97,193.76) | -2.01(-2.27,-1.75) |
| Fiji | 364.85(271.71,476.67) | 843.04(628.01,1127.48) | 2.19(1.97,2.40) | 589.54(550.89,630.25) | 1014.85(968.42,1062.95) | 1.61(1.42,1.79) |
| Finland | 265.56(166.88,386.23) | 551.35(350.52,798.12) | 2.46(2.15,2.78) | 61.87(57.05,67.03) | 149.33(141.38,157.65) | 2.82(2.66,2.98) |
| France | 1463.72(1007.57,2059.85) | 3044.91(1913.78,4486.61) | 2.71(2.52,2.91) | 33.11(32.06,34.18) | 72.78(71.15,74.44) | 3.06(2.85,3.27) |
| Gabon | 99.54(69.18,131.56) | 340.71(244.78,458.44) | 3.79(3.49,4.10) | 174.96(155.03,196.87) | 263.68(247.23,280.98) | 1.19(0.93,1.45) |
| Gambia | 50.48(34.62,70.55) | 307.76(224.05,421.17) | 5.38(5.13,5.63) | 121.51(105.58,139.39) | 250.99(236.93,265.72) | 2.20(2.00,2.40) |
| Georgia | 245.75(162.21,346.73) | 421.68(288.41,607.21) | 0.87(0.66,1.09) | 57.11(52.75,61.75) | 138.77(129.64,148.46) | 3.00(2.81,3.19) |
| Germany | 2521.83(1825.85,3482.20) | 5654.08(3720.97,7974.61) | 1.96(1.59,2.33) | 43.71(42.67,44.77) | 102.65(100.94,104.40) | 2.87(2.64,3.10) |
| Ghana | 1525.20(1110.90,2015.92) | 5490.46(3938.07,7210.96) | 4.02(3.90,4.13) | 191.73(186.51,197.05) | 244.15(240.54,247.81) | 0.77(0.62,0.91) |
| Greece | 530.98(351.15,743.79) | 1090.13(714.43,1552.06) | 1.62(1.26,1.98) | 81.32(77.32,85.48) | 168.04(161.52,174.78) | 2.33(2.26,2.40) |
| Greenland | 1.32(0.88,1.85) | 2.54(1.80,3.46) | -0.15(-0.88,0.60) | 34.26(9.36,96.95) | 55.34(19.97,126.81) | 1.10(0.70,1.50) |
| Grenada | 21.62(17.69,26.56) | 32.87(24.76,43.59) | 0.10(-0.15,0.35) | 515.48(407.34,644.80) | 490.89(395.43,603.00) | -0.46(-0.69,-0.24) |
| Guam | 22.74(17.05,29.65) | 39.25(28.36,52.64) | 0.99(0.68,1.30) | 217.93(166.92,279.90) | 357.12(288.60,437.17) | 1.51(1.35,1.67) |
| Guatemala | 868.58(683.51,1098.59) | 5882.96(4659.52,7291.77) | 5.33(5.07,5.60) | 197.40(189.96,205.08) | 446.47(439.18,453.86) | 2.32(2.08,2.56) |
| Guinea | 409.88(294.35,559.79) | 1699.07(1207.12,2238.07) | 4.97(4.93,5.01) | 139.42(132.40,146.73) | 258.78(252.64,265.05) | 2.03(1.95,2.10) |
| Guinea-Bissau | 108.32(78.17,145.45) | 419.29(309.98,560.37) | 4.25(4.04,4.46) | 224.02(202.62,247.19) | 357.36(339.42,376.05) | 1.39(1.14,1.64) |
| Guyana | 227.26(176.77,288.92) | 393.78(284.33,531.68) | 0.98(0.72,1.23) | 468.87(435.14,504.64) | 743.70(700.30,789.16) | 1.37(1.15,1.59) |
| Haiti | 1962.43(1393.04,2638.02) | 6207.50(4492.19,8500.40) | 3.55(3.46,3.64) | 532.23(519.26,545.45) | 630.54(621.29,639.90) | 0.62(0.54,0.71) |
| Honduras | 491.96(370.71,669.46) | 2156.90(1547.46,3030.66) | 4.49(4.37,4.60) | 186.81(177.23,196.80) | 274.56(267.56,281.70) | 1.02(0.89,1.16) |
| Hungary | 584.50(421.70,796.16) | 553.43(375.07,778.23) | 0.24(-0.06,0.54) | 54.49(51.30,57.84) | 64.70(60.75,68.88) | 0.59(0.42,0.75) |
| Iceland | 9.67(6.15,14.06) | 29.12(18.96,41.15) | 3.26(3.20,3.31) | 57.89(39.01,82.98) | 132.46(105.11,165.28) | 2.73(2.69,2.77) |
| India | 59053.48(44559.98,76432.51) | 205460.07(145987.70,278601.19) | 3.56(3.49,3.63) | 116.67(116.13,117.21) | 190.58(190.08,191.08) | 1.43(1.37,1.49) |
| Indonesia | 14475.15(11650.91,17800.05) | 35251.11(27749.96,44995.80) | 0.54(0.16,0.91) | 121.47(120.33,122.62) | 136.47(135.51,137.42) | -0.85(-1.23,-0.46) |
| Iran (Islamic Republic of) | 2049.01(1506.53,2700.97) | 14284.88(10099.03,19616.75) | 5.22(4.89,5.54) | 64.78(63.13,66.46) | 145.42(143.71,147.15) | 2.47(2.26,2.69) |
| Iraq | 2143.93(1608.14,2818.53) | 11183.80(7811.32,15529.52) | 5.14(4.81,5.47) | 240.08(234.47,245.80) | 424.71(420.15,429.30) | 1.90(1.72,2.09) |
| Ireland | 134.49(88.33,192.13) | 359.06(225.48,526.76) | 2.85(2.59,3.12) | 58.17(52.52,64.28) | 88.99(82.71,95.64) | 1.51(1.44,1.58) |
| Israel | 286.65(209.21,382.11) | 652.86(434.54,953.27) | 2.29(2.22,2.35) | 86.94(81.09,93.10) | 96.46(91.77,101.32) | 0.30(0.21,0.39) |
| Italy | 2734.80(1861.00,3791.95) | 3386.00(2111.87,5032.83) | 0.50(0.02,0.99) | 65.74(64.21,67.30) | 87.01(85.04,89.03) | 1.24(0.98,1.50) |
| Jamaica | 316.11(254.31,382.51) | 749.95(570.95,993.30) | 1.75(1.30,2.21) | 217.73(204.11,232.07) | 328.34(314.09,343.10) | 1.00(0.65,1.36) |
| Japan | 6186.53(4419.53,8448.62) | 8874.89(5921.46,12412.92) | 1.47(1.20,1.74) | 62.79(61.76,63.84) | 118.57(116.94,120.22) | 2.20(2.13,2.26) |
| Jordan | 317.47(242.35,410.46) | 2047.09(1424.53,2798.25) | 5.10(4.92,5.29) | 184.91(173.70,196.71) | 239.91(233.61,246.35) | 0.56(0.31,0.81) |
| Kazakhstan | 998.79(664.02,1435.40) | 3126.13(2119.38,4459.17) | 3.48(3.32,3.64) | 77.27(74.34,80.29) | 191.31(186.94,195.77) | 2.96(2.71,3.20) |
| Kenya | 1081.57(847.05,1339.70) | 4465.50(3454.53,5573.24) | 3.90(3.73,4.07) | 100.12(96.85,103.49) | 120.50(118.34,122.69) | 0.70(0.59,0.81) |
| Kiribati | 36.40(27.49,46.50) | 105.63(77.79,141.47) | 3.27(3.22,3.32) | 661.07(533.52,811.00) | 1027.83(905.42,1162.38) | 1.50(1.43,1.58) |
| Kuwait | 168.81(116.60,236.03) | 1801.74(1184.62,2496.48) | 7.37(6.88,7.87) | 138.86(126.70,151.91) | 288.24(278.59,298.23) | 2.17(2.00,2.34) |
| Kyrgyzstan | 155.37(107.12,222.60) | 631.12(433.25,889.49) | 3.72(3.58,3.87) | 54.72(49.84,59.98) | 113.44(107.87,119.23) | 2.33(2.19,2.47) |
| Lao People's Democratic Republic | 430.18(294.38,595.00) | 1293.41(926.01,1768.43) | 2.94(2.74,3.14) | 178.47(168.73,188.66) | 227.38(219.94,235.01) | 0.44(0.28,0.59) |
| Latvia | 98.39(65.88,141.89) | 175.54(127.85,244.36) | 1.17(0.85,1.49) | 41.65(36.22,47.71) | 109.81(98.27,122.55) | 2.95(2.59,3.32) |
| Lebanon | 359.80(249.41,483.56) | 1200.71(843.00,1655.01) | 4.33(4.00,4.65) | 152.03(142.02,162.59) | 233.52(224.99,242.34) | 1.50(1.37,1.62) |
| Lesotho | 55.15(36.96,76.75) | 211.30(143.38,295.55) | 5.43(5.00,5.86) | 57.08(48.59,66.67) | 163.67(151.15,176.99) | 4.40(3.93,4.86) |
| Liberia | 165.42(121.75,222.41) | 880.37(631.16,1190.45) | 6.43(6.02,6.84) | 144.50(133.57,156.13) | 301.11(290.83,311.67) | 2.44(2.23,2.65) |
| Libya | 179.30(128.51,237.73) | 1492.25(1026.50,2059.99) | 6.70(6.13,7.28) | 86.27(78.97,94.11) | 220.59(213.21,228.17) | 3.44(3.23,3.65) |
| Lithuania | 105.27(64.85,157.08) | 168.57(112.54,237.17) | 0.59(0.34,0.84) | 29.94(26.07,34.25) | 70.40(62.84,78.77) | 2.64(2.53,2.75) |
| Luxembourg | 17.46(11.41,24.64) | 60.78(38.60,89.16) | 3.53(3.32,3.73) | 62.41(46.13,83.63) | 125.13(105.95,147.42) | 2.23(2.18,2.28) |
| Madagascar | 1116.15(803.63,1492.85) | 3509.65(2528.93,4863.88) | 3.63(3.51,3.76) | 177.89(172.06,183.89) | 206.06(202.22,209.96) | 0.42(0.30,0.54) |
| Malawi | 735.37(514.52,982.83) | 1723.75(1231.38,2259.04) | 2.42(2.29,2.55) | 143.93(138.11,149.95) | 144.94(141.06,148.90) | -0.24(-0.40,-0.08) |
| Malaysia | 1554.01(1136.76,2044.58) | 4497.18(3245.37,6149.76) | 3.26(3.10,3.42) | 115.89(112.33,119.53) | 166.68(163.61,169.79) | 1.26(1.15,1.36) |
| Maldives | 14.66(10.67,21.04) | 52.36(37.15,72.94) | 2.70(2.37,3.03) | 148.68(108.62,199.93) | 118.73(98.06,143.14) | -0.97(-1.36, -0.58) |
| Mali | 936.76(700.02,1225.63) | 3911.64(2792.02,5393.20) | 5.45(5.29,5.61) | 237.20(229.38,245.22) | 401.97(395.82,408.20) | 1.82(1.56,2.08) |
| Malta | 17.59(12.60,24.15) | 44.79(29.15,64.33) | 3.42(3.26,3.58) | 59.95(43.45,81.16) | 145.29(119.04,176.73) | 3.12(3.03,3.22) |
| Marshall Islands | 18.45(13.80,24.60) | 78.98(53.57,110.86) | 4.60(4.15,5.06) | 628.07(458.71,841.55) | 1646.05(1416.79,1902.31) | 3.18(2.85,3.52) |
| Mauritania | 109.77(78.06,148.26) | 372.28(262.45,503.00) | 3.37(3.16,3.58) | 119.12(108.25,130.84) | 168.20(159.36,177.42) | 0.85(0.66,1.04) |
| Mauritius | 151.99(118.35,190.06) | 386.63(311.76,475.86) | 4.04(3.64,4.43) | 146.14(131.25,162.29) | 394.03(368.85,420.55) | 4.24(3.83,4.66) |
| Mexico | 20397.31(16531.92,24988.18) | 48950.10(37603.31,62518.65) | 1.90(1.81,2.00) | 365.88(362.90,368.89) | 415.58(413.13,418.03) | 0.39(0.26,0.52) |
| Micronesia (Federated States of) | 28.75(20.82,39.92) | 58.71(42.20,76.71) | 2.33(1.95,2.73) | 410.90(320.41,519.87) | 780.35(658.74,918.37) | 2.30(1.96,2.63) |
| Monaco | 1.01(0.66,1.46) | 2.49(1.58,3.55) | 2.49(2.39,2.60) | 55.03(10.18,203.84) | 123.00(45.60,275.95) | 2.66(2.63,2.68) |
| Mongolia | 58.64(41.11,82.27) | 336.83(230.90,474.17) | 4.32(4.17,4.47) | 52.87(45.65,60.99) | 115.20(107.25,123.63) | 2.60(2.49,2.70) |
| Montenegro | 26.97(17.33,38.87) | 43.61(28.28,63.77) | 1.06(0.82,1.29) | 42.88(32.12,56.14) | 62.86(49.10,79.65) | 1.28(1.01,1.55) |
| Morocco | 1767.31(1224.31,2434.10) | 10157.37(6840.04,14200.00) | 5.45(5.22,5.69) | 106.74(103.79,109.75) | 333.23(329.10,337.40) | 3.93(3.79,4.07) |
| Mozambique | 1135.43(777.79,1595.26) | 3160.22(2188.35,4384.91) | 4.24(4.11,4.37) | 127.45(122.93,132.10) | 180.82(177.22,184.47) | 1.42(1.28,1.57) |
| Myanmar | 11589.13(7913.07,16439.15) | 15926.18(12076.24,20474.41) | -0.23(-0.55,0.10) | 396.10(391.68,400.55) | 319.00(315.71,322.32) | -1.24(-1.46, -1.03) |
| Namibia | 66.30(47.26,89.60) | 224.92(160.43,299.73) | 3.24(2.97,3.51) | 88.86(77.44,101.59) | 129.63(119.94,139.91) | 1.09(0.92,1.26) |
| Nauru | 4.41(3.33,5.85) | 8.59(6.22,11.60) | 2.11(1.86,2.36) | 537.77(258.31,994.33) | 967.73(598.38,1484.58) | 1.81(1.62,2.01) |
| Nepal | 1429.36(1035.68,1928.27) | 6092.23(4276.26,8331.05) | 4.45(4.12,4.79) | 139.90(135.95,143.93) | 266.59(262.76,270.45) | 2.05(1.71,2.39) |
| Netherlands | 434.88(286.54,609.65) | 889.40(558.18,1276.46) | 1.90(1.70,2.10) | 36.93(34.82,39.15) | 84.83(81.42,88.36) | 2.62(2.49,2.76) |
| New Zealand | 152.49(109.31,206.65) | 386.51(278.31,517.86) | 3.38(2.99,3.77) | 47.04(42.11,52.41) | 101.88(95.48,108.63) | 2.83(2.47,3.20) |
| Nicaragua | 454.02(341.81,591.03) | 1530.91(1103.10,2077.18) | 3.27(3.17,3.36) | 205.77(194.82,217.20) | 274.04(265.48,282.82) | 0.86(0.76,0.96) |
| Niger | 366.95(267.30,494.85) | 1843.58(1331.19,2503.95) | 5.17(5.00,5.34) | 118.22(112.33,124.36) | 209.05(204.42,213.76) | 1.65(1.53,1.78) |
| Nigeria | 4759.80(3517.36,6103.12) | 17702.42(12764.67,23641.06) | 3.90(3.70,4.10) | 113.62(111.89,115.37) | 145.03(143.87,146.19) | 0.59(0.46,0.72) |
| Niue | 0.61(0.46,0.82) | 1.04(0.79,1.32) | 1.43(1.09,1.76) | 391.53(25.11,1749.97) | 1014.39(193.41,3097.45) | 2.62(2.44,2.79) |
| North Macedonia | 90.85(63.53,127.76) | 167.54(110.25,241.92) | 1.27(1.03,1.50) | 53.73(46.71,61.51) | 67.39(59.86,75.79) | 0.81(0.56,1.07) |
| Northern Mariana Islands | 13.89(10.22,18.56) | 15.15(11.20,19.92) | -1.33(-2.50, -0.16) | 290.42(200.42,408.87) | 420.26(290.25,591.91) | 1.21(1.09,1.34) |
| Norway | 205.40(136.65,290.81) | 337.66(218.17,491.12) | 1.35(1.24,1.45) | 66.25(60.74,72.15) | 93.10(86.97,99.57) | 0.97(0.89,1.04) |
| Oman | 90.90(62.23,125.87) | 658.06(452.94,911.09) | 6.02(5.55,6.49) | 88.24(77.15,100.52) | 177.41(168.67,186.55) | 1.70(1.37,2.03) |
| Pakistan | 7384.32(5482.37,9633.85) | 45095.87(33116.38,58987.63) | 5.87(5.60,6.13) | 134.37(132.60,136.16) | 285.87(284.33,287.41) | 2.31(2.05,2.57) |
| Palau | 5.31(3.93,6.91) | 9.14(6.79,12.14) | 1.15(0.57,1.73) | 407.37(217.25,698.62) | 848.28(523.13,1312.19) | 2.55(2.42,2.69) |
| Palestine | 114.48(83.39,158.68) | 630.62(462.72,824.92) | 5.20(5.15,5.26) | 130.03(117.44,143.70) | 198.17(189.36,207.31) | 1.42(1.25,1.60) |
| Panama | 259.60(200.68,329.97) | 816.08(601.49,1082.16) | 2.91(2.81,3.01) | 175.24(163.32,187.85) | 257.96(246.98,269.31) | 1.24(1.11,1.38) |
| Papua New Guinea | 1093.33(696.75,1542.26) | 4912.83(3680.76,6561.62) | 4.80(4.73,4.87) | 412.67(397.69,428.09) | 642.52(631.51,653.68) | 1.30(1.22,1.37) |
| Paraguay | 375.34(280.75,484.74) | 1189.91(859.56,1557.76) | 3.13(2.96,3.29) | 170.07(160.68,179.90) | 214.79(207.41,222.37) | 0.80(0.65,0.94) |
| Peru | 1016.87(755.48,1371.93) | 2955.76(2226.91,4015.26) | 2.87(2.59,3.14) | 78.41(75.69,81.21) | 104.28(101.99,106.62) | 0.97(0.71,1.23) |
| Philippines | 5943.45(5040.78,7050.47) | 14898.49(11927.82,18705.45) | 2.24(2.03,2.45) | 164.20(161.86,166.55) | 183.22(181.43,185.01) | 0.24(0.00,0.49) |
| Poland | 2150.16(1511.42,2890.50) | 2533.89(1715.71,3569.37) | 0.63(0.06,1.21) | 47.29(45.80,48.81) | 54.57(52.89,56.30) | 0.53(0.22,0.85) |
| Portugal | 729.59(546.84,966.96) | 1339.72(886.34,1868.52) | 1.44(1.28,1.60) | 113.82(109.07,118.73) | 203.68(196.69,210.87) | 1.90(1.75,2.05) |
| Puerto Rico | 581.91(429.22,769.22) | 733.18(506.01,1026.16) | 0.38(0.29,0.46) | 207.54(197.17,218.32) | 314.45(299.77,329.69) | 1.30(1.15,1.45) |
| Qatar | 40.51(28.55,54.00) | 558.31(375.24,768.79) | 9.06(8.43,9.70) | 141.09(114.50,172.60) | 253.41(239.01,268.69) | 1.75(1.49,2.02) |
| Republic of Korea | 3615.70(2674.25,4703.05) | 9001.91(6079.52,12522.44) | 2.38(1.97,2.78) | 117.95(115.84,120.10) | 266.03(262.51,269.60) | 3.11(2.60,3.63) |
| Republic of Moldova | 244.72(160.26,356.97) | 493.72(326.15,721.27) | 1.64(1.34,1.94) | 53.55(49.02,58.42) | 118.36(110.60,126.64) | 2.41(2.22,2.60) |
| Romania | 885.52(621.04,1245.85) | 853.10(565.91,1207.52) | -0.34(-0.56, -0.13) | 41.92(40.01,43.89) | 46.85(44.44,49.36) | 0.51(0.33,0.70) |
| Russian Federation | 5670.99(3821.17,7939.45) | 11872.35(8388.51,16239.82) | 1.42(1.14,1.70) | 37.58(36.90,38.26) | 68.75(67.78,69.73) | 1.63(1.47,1.80) |
| Rwanda | 970.02(567.03,1455.05) | 1327.70(890.20,1953.00) | 0.17(-0.25,0.59) | 221.88(213.62,230.39) | 131.56(127.27,135.96) | -2.67(-3.08, -2.26) |
| Saint Kitts and Nevis | 9.48(7.68,11.88) | 14.47(10.02,20.68) | 0.12(-0.22,0.46) | 376.54(254.62,539.19) | 285.52(197.86,401.64) | -1.11(-1.52, -0.70) |
| Saint Lucia | 34.09(26.93,42.64) | 69.12(50.50,95.41) | 1.04(0.84,1.24) | 442.01(362.82,534.38) | 466.81(397.30,545.90) | 0.15(-0.03,0.34) |
| Saint Vincent and the Grenadines | 25.00(19.83,30.75) | 45.80(34.45,60.64) | -0.35(-0.65, -0.05) | 474.42(382.32,583.68) | 531.79(437.06,641.35) | -0.33(-0.58, -0.08) |
| Samoa | 32.68(24.79,44.31) | 105.89(77.31,143.14) | 3.72(3.56,3.89) | 341.54(273.20,422.54) | 782.06(692.86,879.88) | 2.89(2.75,3.03) |
| San Marino | 0.88(0.59,1.25) | 2.33(1.49,3.33) | 2.22(1.83,2.62) | 59.04(11.22,183.56) | 123.94(44.84,278.80) | 2.44(2.39,2.48) |
| Sao Tome and Principe | 4.64(3.24,6.52) | 27.52(19.05,39.24) | 5.38(5.20,5.56) | 97.30(57.56,156.22) | 229.14(185.91,279.69) | 2.69(2.52,2.86) |
| Saudi Arabia | 972.87(721.32,1310.13) | 10063.80(7361.64,13708.46) | 7.60(7.32,7.88) | 117.87(113.51,122.37) | 262.74(259.30,266.22) | 2.95(2.76,3.14) |
| Senegal | 600.90(444.58,813.34) | 2367.60(1742.05,3150.97) | 4.30(3.98,4.63) | 190.48(183.03,198.18) | 297.86(291.71,304.11) | 1.68(1.43,1.92) |
| Serbia | 510.04(354.04,708.56) | 598.58(413.96,833.06) | -0.49(-0.67, -0.31) | 58.37(54.94,61.98) | 62.54(58.73,66.56) | 0.04(-0.04,0.13) |
| Seychelles | 4.11(3.04,5.38) | 21.66(16.00,29.62) | 4.17(3.93,4.42) | 97.71(51.60,171.36) | 263.23(193.73,350.53) | 3.48(3.39,3.57) |
| Sierra Leone | 232.56(163.25,310.53) | 1128.42(792.05,1529.18) | 6.09(5.84,6.34) | 109.84(102.65,117.45) | 264.40(256.82,272.17) | 3.07(2.79,3.35) |
| Singapore | 332.36(245.43,448.14) | 950.79(618.72,1389.18) | 3.38(3.09,3.66) | 108.75(101.48,116.45) | 188.70(180.41,197.39) | 1.72(1.49,1.96) |
| Slovakia | 208.92(139.07,298.97) | 304.38(198.43,438.44) | 1.26(1.09,1.42) | 36.05(32.57,39.82) | 47.67(43.54,52.17) | 1.09(1.01,1.17) |
| Slovenia | 75.93(52.42,108.55) | 106.29(69.97,152.27) | 0.57(0.47,0.67) | 35.98(30.39,42.38) | 50.33(43.01,58.77) | 1.02(0.95,1.09) |
| Solomon Islands | 71.39(37.72,107.17) | 398.52(283.71,543.42) | 5.47(5.29,5.65) | 360.42(308.82,418.58) | 722.83(676.63,771.39) | 2.34(2.27,2.41) |
| Somalia | 852.44(562.80,1252.65) | 2561.86(1775.09,3552.42) | 4.57(4.37,4.76) | 165.94(159.04,173.08) | 214.24(209.35,219.22) | 0.78(0.71,0.85) |
| South Africa | 5157.35(4398.11,6164.76) | 11527.02(9440.08,14203.96) | 1.60(0.84,2.36) | 217.74(214.34,221.19) | 214.76(212.21,217.33) | 0.03(-0.70,0.76) |
| South Sudan | 389.64(259.48,554.35) | 1263.16(855.26,1735.89) | 3.65(3.40,3.91) | 130.97(123.59,138.70) | 209.65(202.77,216.72) | 1.47(1.12,1.81) |
| Spain | 1927.34(1355.64,2661.15) | 5300.64(3479.72,7557.98) | 2.68(2.47,2.88) | 79.45(77.40,81.54) | 173.46(170.31,176.65) | 2.45(1.88,3.03) |
| Sri Lanka | 1744.11(1288.44,2268.94) | 4782.10(3414.94,6843.26) | 2.80(2.53,3.07) | 142.22(138.34,146.19) | 266.07(261.17,271.04) | 2.23(1.99,2.46) |
| Sudan | 910.84(651.73,1255.19) | 5749.84(4078.74,7877.87) | 5.97(5.89,6.06) | 78.74(75.78,81.79) | 188.78(185.89,191.70) | 2.92(2.84,3.00) |
| Suriname | 60.40(43.83,77.95) | 213.14(156.93,289.81) | 3.13(2.93,3.32) | 271.28(233.97,313.17) | 470.38(430.82,512.68) | 1.70(1.40,2.01) |
| Sweden | 417.35(294.93,574.64) | 695.95(463.69,991.93) | 1.54(1.33,1.74) | 69.03(64.88,73.39) | 103.45(98.67,108.42) | 1.20(1.07,1.34) |
| Switzerland | 413.74(285.30,575.96) | 1056.09(666.06,1518.86) | 2.51(2.26,2.75) | 88.29(83.34,93.49) | 172.92(166.32,179.76) | 2.24(2.06,2.41) |
| Syrian Arab Republic | 744.65(557.75,982.57) | 2257.17(1554.08,3138.52) | 2.17(1.49,2.85) | 131.82(126.71,137.09) | 193.41(187.79,199.18) | 1.00(0.82,1.18) |
| Taiwan (Province of China) | 1751.07(1261.52,2336.21) | 2333.61(1645.85,3220.12) | -0.04(-0.27,0.19) | 104.73(101.80,107.73) | 131.46(127.94,135.08) | 0.16(-0.08,0.41) |
| Tajikistan | 208.94(146.66,280.84) | 957.15(660.09,1329.08) | 3.33(2.97,3.68) | 77.33(71.73,83.29) | 123.42(118.65,128.35) | 0.95(0.68,1.22) |
| Thailand | 4544.76(3311.52,5899.57) | 8839.36(6477.91,11490.66) | 0.51(-0.41,1.43) | 97.65(95.92,99.41) | 163.56(161.24,165.90) | 0.83(0.11,1.56) |
| Timor-Leste | 36.27(24.96,49.45) | 107.34(74.15,148.41) | 3.52(2.93,4.11) | 79.85(65.83,96.12) | 144.20(129.24,160.53) | 1.94(1.57,2.31) |
| Togo | 195.25(139.87,266.65) | 912.18(649.64,1251.67) | 4.50(4.32,4.68) | 120.85(112.56,129.63) | 188.80(182.32,195.46) | 1.45(1.35,1.54) |
| Tokelau | 0.58(0.41,0.78) | 0.79(0.60,1.04) | 1.08(0.57,1.59) | 460.64(21.93,2326.13) | 883.45(113.42,3174.92) | 1.68(1.51,1.85) |
| Tonga | 25.95(19.75,33.34) | 52.11(38.05,70.55) | 2.14(2.03,2.26) | 428.70(329.08,550.08) | 677.77(563.92,808.20) | 1.43(1.38,1.47) |
| Trinidad and Tobago | 374.41(312.43,455.69) | 671.84(499.36,890.95) | 0.61(0.40,0.82) | 416.80(391.30,443.57) | 513.23(486.45,541.23) | 0.25(0.09,0.41) |
| Tunisia | 387.09(259.34,540.92) | 2121.95(1425.22,3012.92) | 4.82(4.59,5.05) | 73.19(68.90,77.69) | 193.74(188.20,199.40) | 3.25(3.20,3.29) |
| Türkiye | 3758.60(2717.71,4996.04) | 10952.15(7579.77,15014.39) | 2.73(2.40,3.06) | 110.11(108.13,112.11) | 157.56(155.64,159.50) | 1.36(0.95,1.76) |
| Turkmenistan | 115.69(81.84,155.93) | 551.67(418.25,726.42) | 5.02(4.68,5.37) | 51.09(45.92,56.71) | 173.82(165.60,182.34) | 4.21(3.89,4.52) |
| Tuvalu | 3.63(2.69,4.93) | 5.75(4.18,7.50) | 1.72(1.49,1.95) | 409.79(175.35,817.43) | 645.55(351.35,1092.15) | 1.45(1.40,1.51) |
| Uganda | 805.28(534.92,1182.14) | 3489.56(2421.05,4744.46) | 4.12(3.63,4.61) | 93.00(89.33,96.79) | 149.82(147.05,152.64) | 0.74(0.31,1.17) |
| Ukraine | 2404.02(1629.42,3261.40) | 4019.37(2734.26,5612.08) | 0.25(0.02,0.48) | 54.43(53.01,55.88) | 84.75(82.77,86.78) | 0.74(0.44,1.04) |
| United Arab Emirates | 84.47(58.20,115.01) | 1460.43(993.98,2050.44) | 8.89(8.17,9.60) | 81.43(71.46,92.49) | 156.92(149.45,164.73) | 2.09(1.98,2.21) |
| United Kingdom | 2882.90(2033.96,3978.09) | 10238.18(6750.29,14699.31) | 3.87(3.59,4.15) | 77.54(75.88,79.24) | 265.36(262.41,268.34) | 3.71(3.44,3.98) |
| United Republic of Tanzania | 1593.46(1128.84,2183.49) | 5440.22(3940.56,7409.13) | 3.85(3.75,3.96) | 112.77(109.60,116.01) | 150.96(148.65,153.29) | 0.87(0.78,0.96) |
| United States of America | 16139.48(12918.21,20149.22) | 37175.22(27330.85,49837.27) | 2.51(2.29,2.73) | 64.83(64.16,65.50) | 140.88(139.91,141.84) | 2.50(2.39,2.60) |
| United States Virgin Islands | 22.16(16.19,30.54) | 21.90(14.92,30.82) | -0.13(-0.55,0.30) | 245.84(183.58,323.14) | 379.54(279.52,506.40) | 1.50(1.33,1.67) |
| Uruguay | 110.17(87.55,138.35) | 236.26(168.90,322.45) | 2.05(1.90,2.21) | 45.13(39.79,51.00) | 81.36(74.45,88.76) | 1.72(1.58,1.87) |
| Uzbekistan | 822.05(593.33,1105.35) | 4656.25(3250.47,6414.85) | 4.57(4.19,4.95) | 64.46(61.96,67.03) | 158.07(155.18,161.00) | 2.65(2.33,2.96) |
| Vanuatu | 29.04(19.88,42.29) | 138.13(103.63,175.07) | 4.83(4.73,4.94) | 278.09(217.69,350.67) | 589.45(528.56,655.58) | 2.18(2.08,2.27) |
| Venezuela (Bolivarian Republic of) | 2346.01(1852.65,2982.05) | 6470.02(4782.57,8510.13) | 3.14(2.83,3.45) | 180.01(175.75,184.35) | 292.57(287.92,297.27) | 1.62(1.25,1.99) |
| Viet Nam | 5297.63(3869.07,7297.68) | 12339.46(9130.05,16246.27) | 1.86(1.65,2.07) | 100.82(99.11,102.57) | 121.44(119.94,122.96) | 0.45(0.14,0.75) |
| Yemen | 482.74(333.89,675.69) | 3215.88(2196.39,4498.04) | 5.55(4.97,6.14) | 60.57(57.27,64.02) | 128.71(125.95,131.52) | 1.52(0.98,2.06) |
| Zambia | 793.95(570.36,1067.64) | 2859.20(2096.48,3873.60) | 3.64(3.35,3.94) | 214.81(206.63,223.25) | 251.59(246.47,256.80) | 0.15(-0.02,0.32) |
| Zimbabwe | 392.07(290.47,531.60) | 1727.35(1201.06,2342.47) | 5.24(4.72,5.76) | 76.47(72.43,80.70) | 176.11(171.49,180.83) | 3.49(2.94,4.04) |
