# Supplementary material for: Assessing the global burden of Type 2 diabetes in women of reproductive age
Source: PLoS One. 2025 Jul 14;20(7):e0322787. doi: 10.1371/journal.pone.0322787 (PMC12258576; doi:10.1371/journal.pone.0322787)
Supplement: S3 Table — (DOCX) [file pone.0322787.s006.docx]

**Table S3. The number and its trend of Incidence and DALY for type 2 diabetes burden mellitus in women of childbearing age in 1990 and 2021 in different regions.**

| Country | sex | nDALY_90 | nDALY_21 | n incidence_  1990 | n incidence_  2021 | 1990-2021  time rend_  DALY | 1990-2021  time trend_  incidence |
| --- | --- | --- | --- | --- | --- | --- | --- |
| global | female | 1173672.72 | 2980835.34 | 1074988.25 | 3029424.16 | 2.54 | 2.82 |
| High-middle SDI | female | 187163.76 | 415308.05 | 216540.19 | 434258.75 | 2.22 | 2.01 |
| High SDI | female | 116996.43 | 278971.49 | 134820.75 | 354029.58 | 2.38 | 2.63 |
| Low-middle SDI | female | 282020.30 | 847588.41 | 223197.95 | 875646.99 | 3.01 | 3.92 |
| Low SDI | female | 132156.64 | 456179.71 | 75647.56 | 376053.23 | 3.45 | 4.97 |
| Middle SDI | female | 453885.36 | 979653.30 | 423715.76 | 986617.31 | 2.16 | 2.33 |
| Central Europe, eastern Europe, and central Asia | female | 40317.60 | 73013.79 | 55883.95 | 106708.02 | 1.81 | 1.91 |
| High-income | female | 114486.83 | 240557.83 | 131394.31 | 323263.59 | 2.10 | 2.46 |
| Latin America and Caribbean | female | 165497.88 | 306415.34 | 116480.37 | 284690.03 | 1.85 | 2.44 |
| North Africa and Middle East | female | 62938.68 | 286685.15 | 59010.07 | 351914.99 | 4.55 | 5.96 |
| South Asia | female | 242620.57 | 814572.57 | 237212.70 | 937184.86 | 3.36 | 3.95 |
| Southeast Asia, east Asia, and Oceania | female | 409752.95 | 828089.81 | 413142.43 | 746303.47 | 2.02 | 1.81 |
| Sub-Saharan Africa | female | 138058.23 | 431500.85 | 61864.42 | 279359.21 | 3.13 | 4.52 |
| Andean Latin America | female | 7101.73 | 18076.87 | 4830.33 | 16990.94 | 2.55 | 3.52 |
| Australasia | female | 1388.39 | 3193.33 | 1998.86 | 4979.05 | 2.30 | 2.49 |
| Caribbean | female | 18954.90 | 39702.83 | 12096.75 | 32296.26 | 2.09 | 2.67 |
| Central Asia | female | 9226.77 | 30522.00 | 10728.32 | 37801.40 | 3.31 | 3.52 |
| Central Europe | female | 11600.16 | 11765.17 | 17207.70 | 21285.86 | 1.01 | 1.24 |
| Central Latin America | female | 87017.34 | 179955.41 | 69130.94 | 168987.21 | 2.07 | 2.44 |
| Central sub-Saharan Africa | female | 15878.84 | 61948.97 | 7908.03 | 44793.89 | 3.90 | 5.66 |
| East Asia | female | 270971.10 | 576639.01 | 334379.19 | 532572.14 | 2.13 | 1.59 |
| Eastern Europe | female | 19490.67 | 30726.62 | 27947.93 | 47620.76 | 1.58 | 1.70 |
| Eastern Sub-Saharan Africa | female | 58238.08 | 141270.46 | 19255.67 | 78008.38 | 2.43 | 4.05 |
| High-income Asia Pacific | female | 27075.28 | 45448.47 | 30610.98 | 53396.21 | 1.68 | 1.74 |
| High-income North America | female | 36860.59 | 85021.60 | 40481.94 | 128384.81 | 2.31 | 3.17 |
| North Africa and Middle East | female | 62938.68 | 286685.15 | 59010.07 | 351914.99 | 4.55 | 5.96 |
| Oceania | female | 4934.22 | 18173.17 | 2826.40 | 14086.55 | 3.68 | 4.98 |
| South Asia | female | 242620.57 | 814572.57 | 237212.70 | 937184.86 | 3.36 | 3.95 |
| Southeast Asia | female | 133847.63 | 233277.63 | 75936.84 | 199644.77 | 1.74 | 2.63 |
| Southern Latin America | female | 5569.19 | 11922.38 | 5972.21 | 18488.85 | 2.14 | 3.10 |
| Southern sub-Saharan Africa | female | 18111.87 | 35463.25 | 9694.38 | 23553.79 | 1.96 | 2.43 |
| Tropical Latin America | female | 52423.91 | 68680.23 | 30422.36 | 66415.62 | 1.31 | 2.18 |
| Western Europe | female | 43593.37 | 94972.06 | 52330.33 | 118014.68 | 2.18 | 2.26 |
| Western sub-Saharan Africa | female | 45829.44 | 192818.17 | 25006.35 | 133003.15 | 4.21 | 5.32 |
