# Supplementary material for: Assessing the global burden of Type 2 diabetes in women of reproductive age
Source: PLoS One. 2025 Jul 14;20(7):e0322787. doi: 10.1371/journal.pone.0322787 (PMC12258576; doi:10.1371/journal.pone.0322787)
Supplement: S4 Table — (DOCX) [file pone.0322787.s007.docx]

**Table S4. The number and its trend of Incidence and DALY type 2 diabetes mellitus burden in women of childbearing age in 1990 and 2021, by countries and regions.**

| Country | Region | nDALY_1990 | nDALY_2021 | n incidence_  1990 | n incidence_  2021 | 1990-2021  time rend_  DALY | 1990-2021  time trend_  incidence |
| --- | --- | --- | --- | --- | --- | --- | --- |
| Afghanistan | North Africa and Middle East | 2617.75 | 25968.19 | 186.52 | 575.54 | 9.92 | 3.09 |
| Albania | Central Europe | 198.25 | 205.06 | 54.42 | 95.69 | 1.03 | 1.76 |
| Algeria | North Africa and Middle East | 4000.53 | 21582.49 | 114.05 | 312.00 | 5.39 | 2.74 |
| American Samoa | Oceania | 37.78 | 82.61 | 371.77 | 975.64 | 2.19 | 2.62 |
| Andorra | Western Europe | 7.35 | 17.39 | 70.54 | 172.80 | 2.37 | 2.45 |
| Angola | Central sub-Saharan Africa | 3011.40 | 14543.48 | 87.78 | 181.62 | 4.83 | 2.07 |
| Antigua and Barbuda | Caribbean | 33.90 | 56.22 | 179.26 | 307.62 | 1.66 | 1.72 |
| Argentina | Southern Latin America | 3905.18 | 8468.80 | 64.33 | 141.94 | 2.17 | 2.21 |
| Armenia | Central Asia | 590.98 | 739.87 | 86.49 | 155.07 | 1.25 | 1.79 |
| Australia | Australasia | 1057.76 | 2243.07 | 43.30 | 79.82 | 2.12 | 1.84 |
| Austria | Western Europe | 547.82 | 1151.39 | 40.72 | 106.55 | 2.10 | 2.62 |
| Azerbaijan | Central Asia | 1044.89 | 3450.56 | 78.93 | 204.97 | 3.30 | 2.60 |
| Bahamas | North Africa and Middle East | 147.61 | 303.76 | 172.64 | 339.44 | 2.06 | 1.97 |
| Bahrain | South Asia | 159.43 | 760.73 | 123.62 | 296.53 | 4.77 | 2.40 |
| Bangladesh | Caribbean | 33956.76 | 104372.77 | 132.09 | 320.13 | 3.07 | 2.42 |
| Barbados | Eastern Europe | 142.25 | 180.86 | 157.45 | 297.38 | 1.27 | 1.89 |
| Belarus | Western Europe | 717.44 | 1075.05 | 54.18 | 98.63 | 1.50 | 1.82 |
| Belgium | Caribbean | 1324.27 | 2715.23 | 77.20 | 184.85 | 2.05 | 2.39 |
| Belize | Western sub-Saharan Africa | 84.79 | 337.51 | 149.21 | 299.68 | 3.98 | 2.01 |
| Benin | Caribbean | 1205.71 | 7548.49 | 82.79 | 221.11 | 6.26 | 2.67 |
| Bermuda | South Asia | 17.17 | 20.11 | 97.02 | 198.01 | 1.17 | 2.04 |
| Bhutan | Andean Latin America | 123.10 | 276.81 | 111.70 | 201.95 | 2.25 | 1.81 |
| Bolivia (Plurinational State of) | Central Europe | 1727.64 | 3803.70 | 76.97 | 143.04 | 2.20 | 1.86 |
| Bosnia and Herzegovina | Southern sub-Saharan Africa | 422.23 | 430.44 | 61.01 | 123.76 | 1.02 | 2.03 |
| Botswana | Tropical Latin America | 208.98 | 799.28 | 56.64 | 119.72 | 3.82 | 2.11 |
| Brazil | High-income Asia Pacific | 51181.00 | 65481.79 | 98.73 | 141.20 | 1.28 | 1.43 |
| Brunei Darussalam | Central Europe | 132.18 | 325.93 | 130.84 | 383.61 | 2.47 | 2.93 |
| Bulgaria | Western sub-Saharan Africa | 876.34 | 856.59 | 72.79 | 119.49 | 0.98 | 1.64 |
| Burkina Faso | Eastern Sub-Saharan Africa | 2119.57 | 9138.85 | 53.91 | 133.14 | 4.31 | 2.47 |
| Burundi | Southeast Asia | 1825.19 | 4732.21 | 55.82 | 90.73 | 2.59 | 1.63 |
| Cabo Verde | Western sub-Saharan Africa | 78.34 | 300.41 | 85.60 | 185.47 | 3.83 | 2.17 |
| Cambodia | High-income North America | 2345.90 | 5494.80 | 60.70 | 130.06 | 2.34 | 2.14 |
| Cameroon | Western sub-Saharan Africa | 2857.57 | 16783.47 | 64.33 | 151.81 | 5.87 | 2.36 |
| Canada | Central sub-Saharan Africa | 836.89 | 2589.42 | 25.41 | 106.78 | 3.09 | 4.20 |
| Central African Republic | Western sub-Saharan Africa | 1022.84 | 3774.92 | 118.52 | 272.53 | 3.69 | 2.30 |
| Chad | Southern Latin America | 1244.96 | 7510.20 | 68.17 | 153.28 | 6.03 | 2.25 |
| Chile | East Asia | 1406.22 | 2941.52 | 64.01 | 133.76 | 2.09 | 2.09 |
| China | Central Latin America | 261843.75 | 561900.11 | 121.27 | 266.01 | 2.15 | 2.19 |
| Colombia | Eastern Sub-Saharan Africa | 12264.53 | 21164.81 | 138.29 | 207.67 | 1.73 | 1.50 |
| Comoros | Central sub-Saharan Africa | 145.74 | 363.42 | 67.00 | 145.04 | 2.49 | 2.16 |
| Congo | Central Latin America | 808.34 | 3343.33 | 85.83 | 180.68 | 4.14 | 2.10 |
| Cook Islands | Western sub-Saharan Africa | 19.65 | 25.01 | 433.27 | 841.13 | 1.27 | 1.94 |
| Costa Rica | Central Europe | 898.45 | 2694.75 | 136.05 | 272.12 | 3.00 | 2.00 |
| Croatia | Caribbean | 378.88 | 378.00 | 59.27 | 101.66 | 1.00 | 1.72 |
| Cuba | Western Europe | 3770.08 | 4424.23 | 135.81 | 237.53 | 1.17 | 1.75 |
| Cyprus | Central Europe | 116.49 | 330.49 | 82.37 | 158.88 | 2.84 | 1.93 |
| Czechia | Central sub-Saharan Africa | 774.80 | 1034.24 | 62.09 | 109.24 | 1.33 | 1.76 |
| Cote d'Ivoire | Western Europe | 2769.14 | 14197.45 | 75.24 | 167.19 | 5.13 | 2.22 |
| Democratic People's Republic of Korea | Eastern Sub-Saharan Africa | 4294.99 | 9275.28 | 98.20 | 201.47 | 2.16 | 2.05 |
| Democratic Republic of the Congo | Caribbean | 10610.22 | 38529.20 | 73.28 | 156.97 | 3.63 | 2.14 |
| Denmark | Caribbean | 384.68 | 768.03 | 55.55 | 133.33 | 2.00 | 2.40 |
| Djibouti | Andean Latin America | 72.66 | 407.00 | 43.11 | 86.51 | 5.60 | 2.01 |
| Dominica | North Africa and Middle East | 35.36 | 58.92 | 205.75 | 393.68 | 1.67 | 1.91 |
| Dominican Republic | Central Latin America | 2824.85 | 8017.39 | 134.89 | 309.19 | 2.84 | 2.29 |
| Ecuador | Central sub-Saharan Africa | 2167.51 | 6432.50 | 85.25 | 191.42 | 2.97 | 2.25 |
| Egypt | Eastern Sub-Saharan Africa | 11168.20 | 44446.00 | 60.55 | 223.93 | 3.98 | 3.70 |
| El Salvador | Eastern Europe | 1506.02 | 3629.21 | 112.56 | 236.45 | 2.41 | 2.10 |
| Equatorial Guinea | Eastern Sub-Saharan Africa | 135.24 | 787.53 | 86.57 | 193.36 | 5.82 | 2.23 |
| Eritrea | Oceania | 912.50 | 2914.00 | 57.23 | 121.56 | 3.19 | 2.12 |
| Estonia | Oceania | 103.50 | 183.37 | 64.07 | 149.01 | 1.77 | 2.33 |
| Eswatini | Western Europe | 163.67 | 514.55 | 71.05 | 133.98 | 3.14 | 1.89 |
| Ethiopia | Western Europe | 25411.77 | 40698.53 | 82.73 | 110.67 | 1.60 | 1.34 |
| Fiji | Central sub-Saharan Africa | 873.20 | 1794.87 | 283.11 | 613.42 | 2.06 | 2.17 |
| Finland | Central Asia | 615.67 | 1331.16 | 103.09 | 251.56 | 2.16 | 2.44 |
| France | Western Europe | 3799.02 | 7641.12 | 46.89 | 115.32 | 2.01 | 2.46 |
| Gabon | Western sub-Saharan Africa | 290.78 | 970.50 | 89.12 | 189.46 | 3.34 | 2.13 |
| Gambia | Western Europe | 221.24 | 1228.16 | 65.32 | 150.43 | 5.55 | 2.30 |
| Georgia | High-income North America | 636.81 | 885.31 | 71.37 | 198.46 | 1.39 | 2.78 |
| Germany | Caribbean | 6796.47 | 13899.37 | 54.75 | 161.16 | 2.05 | 2.94 |
| Ghana | Oceania | 5284.99 | 17536.11 | 81.91 | 157.23 | 3.32 | 1.92 |
| Greece | Central Latin America | 1549.37 | 2583.85 | 92.36 | 204.53 | 1.67 | 2.21 |
| Greenland | Western sub-Saharan Africa | 4.14 | 5.87 | 15.49 | 76.85 | 1.42 | 4.96 |
| Grenada | Western sub-Saharan Africa | 79.02 | 92.07 | 212.04 | 370.48 | 1.17 | 1.75 |
| Guam | Caribbean | 61.81 | 94.09 | 188.48 | 370.62 | 1.52 | 1.97 |
| Guatemala | Caribbean | 2721.29 | 14422.05 | 137.64 | 352.01 | 5.30 | 2.56 |
| Guinea | Central Latin America | 1497.52 | 6876.35 | 64.35 | 135.96 | 4.59 | 2.11 |
| Guinea-Bissau | Central Europe | 413.28 | 1505.54 | 95.47 | 197.82 | 3.64 | 2.07 |
| Guyana | Western Europe | 734.69 | 1111.04 | 296.39 | 637.95 | 1.51 | 2.15 |
| Haiti | South Asia | 6470.82 | 17728.46 | 249.93 | 457.13 | 2.74 | 1.83 |
| Honduras | Southeast Asia | 1462.28 | 5888.87 | 145.28 | 283.21 | 4.03 | 1.95 |
| Hungary | North Africa and Middle East | 1120.58 | 1009.88 | 68.24 | 110.15 | 0.90 | 1.61 |
| Iceland | North Africa and Middle East | 29.88 | 80.80 | 72.53 | 184.56 | 2.70 | 2.54 |
| India | Western Europe | 180948.55 | 557546.32 | 115.46 | 227.31 | 3.08 | 1.97 |
| Indonesia | Western Europe | 44162.14 | 78588.99 | 78.01 | 137.33 | 1.78 | 1.76 |
| Iran (Islamic Republic of) | Western Europe | 6057.38 | 29285.11 | 74.36 | 189.48 | 4.83 | 2.55 |
| Iraq | Caribbean | 7227.34 | 33459.56 | 219.01 | 504.23 | 4.63 | 2.30 |
| Ireland | High-income Asia Pacific | 386.97 | 769.14 | 75.62 | 147.62 | 1.99 | 1.95 |
| Israel | North Africa and Middle East | 822.79 | 1593.53 | 79.71 | 141.00 | 1.94 | 1.77 |
| Italy | Central Asia | 7005.18 | 7502.10 | 86.00 | 138.65 | 1.07 | 1.61 |
| Jamaica | Eastern Sub-Saharan Africa | 983.85 | 2001.76 | 114.21 | 247.80 | 2.03 | 2.17 |
| Japan | Oceania | 14188.74 | 20630.04 | 82.38 | 152.93 | 1.45 | 1.86 |
| Jordan | North Africa and Middle East | 1095.19 | 5519.85 | 152.73 | 311.93 | 5.04 | 2.04 |
| Kazakhstan | Central Asia | 2625.58 | 7412.31 | 99.58 | 257.55 | 2.82 | 2.59 |
| Kenya | Southeast Asia | 3838.78 | 12119.89 | 43.55 | 63.83 | 3.16 | 1.47 |
| Kiribati | Eastern Europe | 94.58 | 254.90 | 368.43 | 689.96 | 2.70 | 1.87 |
| Kuwait | North Africa and Middle East | 483.74 | 3782.59 | 169.40 | 399.04 | 7.82 | 2.36 |
| Kyrgyzstan | Southern sub-Saharan Africa | 466.56 | 1564.19 | 64.94 | 158.39 | 3.35 | 2.44 |
| Lao People's Democratic Republic | Western sub-Saharan Africa | 1288.42 | 3542.98 | 86.84 | 189.90 | 2.75 | 2.19 |
| Latvia | North Africa and Middle East | 212.28 | 344.95 | 64.01 | 148.57 | 1.63 | 2.32 |
| Lebanon | Eastern Europe | 866.05 | 2918.38 | 129.01 | 275.89 | 3.37 | 2.14 |
| Lesotho | Western Europe | 163.07 | 642.25 | 44.96 | 112.95 | 3.94 | 2.51 |
| Liberia | Central Europe | 662.10 | 3293.08 | 72.86 | 167.16 | 4.97 | 2.29 |
| Libya | Eastern Sub-Saharan Africa | 546.16 | 3399.95 | 103.61 | 285.52 | 6.23 | 2.76 |
| Lithuania | Eastern Sub-Saharan Africa | 215.17 | 316.64 | 56.12 | 128.31 | 1.47 | 2.29 |
| Luxembourg | Southeast Asia | 49.46 | 154.55 | 76.53 | 176.78 | 3.12 | 2.31 |
| Madagascar | Southeast Asia | 3663.82 | 11295.63 | 51.53 | 92.05 | 3.08 | 1.79 |
| Malawi | Western sub-Saharan Africa | 2441.71 | 5523.28 | 39.99 | 60.33 | 2.26 | 1.51 |
| Malaysia | Western Europe | 4051.30 | 11230.64 | 106.40 | 181.37 | 2.77 | 1.70 |
| Maldives | Oceania | 49.25 | 119.32 | 80.08 | 136.05 | 2.42 | 1.70 |
| Mali | Western sub-Saharan Africa | 3570.99 | 17144.55 | 125.36 | 257.30 | 4.80 | 2.05 |
| Malta | Southeast Asia | 43.99 | 109.28 | 70.30 | 219.38 | 2.48 | 3.12 |
| Marshall Islands | Central Latin America | 46.27 | 184.35 | 437.78 | 1117.56 | 3.98 | 2.55 |
| Mauritania | Eastern Europe | 452.80 | 1399.30 | 60.27 | 112.68 | 3.09 | 1.87 |
| Mauritius | Central Asia | 351.79 | 912.17 | 124.71 | 307.10 | 2.59 | 2.46 |
| Mexico | Central Europe | 59140.04 | 110794.68 | 281.02 | 401.41 | 1.87 | 1.43 |
| Micronesia (Federated States of) | North Africa and Middle East | 71.50 | 147.10 | 281.53 | 611.46 | 2.06 | 2.17 |
| Monaco | Eastern Sub-Saharan Africa | 2.79 | 6.20 | 67.12 | 171.08 | 2.22 | 2.55 |
| Mongolia | Southeast Asia | 203.68 | 791.57 | 60.53 | 151.50 | 3.89 | 2.50 |
| Montenegro | Southern sub-Saharan Africa | 53.03 | 71.78 | 76.92 | 130.91 | 1.35 | 1.70 |
| Morocco | South Asia | 5059.29 | 24894.75 | 140.23 | 468.21 | 4.92 | 3.34 |
| Mozambique | Western Europe | 3067.90 | 10168.90 | 39.79 | 90.82 | 3.31 | 2.28 |
| Myanmar | Australasia | 31273.83 | 35937.19 | 134.36 | 239.96 | 1.15 | 1.79 |
| Namibia | Central Latin America | 229.23 | 665.92 | 60.49 | 102.76 | 2.90 | 1.70 |
| Nauru | Western sub-Saharan Africa | 10.10 | 21.00 | 334.83 | 703.09 | 2.08 | 2.10 |
| Nepal | Western sub-Saharan Africa | 4864.27 | 18652.33 | 128.31 | 287.01 | 3.83 | 2.24 |
| Netherlands | East Asia | 1140.22 | 2331.20 | 50.44 | 124.85 | 2.04 | 2.48 |
| New Zealand | Oceania | 330.64 | 950.26 | 69.09 | 130.16 | 2.87 | 1.88 |
| Nicaragua | Western Europe | 1375.09 | 3875.38 | 151.83 | 268.06 | 2.82 | 1.77 |
| Niger | North Africa and Middle East | 1591.91 | 8463.16 | 73.99 | 150.42 | 5.32 | 2.03 |
| Nigeria | South Asia | 17545.16 | 62698.81 | 62.53 | 100.53 | 3.57 | 1.61 |
| Niue | North Africa and Middle East | 1.43 | 2.80 | 330.80 | 790.55 | 1.96 | 2.39 |
| North Macedonia | Central Latin America | 210.51 | 295.38 | 66.77 | 121.93 | 1.40 | 1.83 |
| Northern Mariana Islands | Oceania | 33.68 | 33.92 | 202.41 | 403.68 | 1.01 | 1.99 |
| Norway | Tropical Latin America | 533.04 | 865.29 | 96.96 | 141.62 | 1.62 | 1.46 |
| Oman | Andean Latin America | 230.83 | 1584.83 | 115.12 | 229.02 | 6.87 | 1.99 |
| Pakistan | Southeast Asia | 22727.89 | 133724.34 | 125.83 | 278.26 | 5.88 | 2.21 |
| Palau | Central Europe | 13.09 | 21.05 | 351.69 | 763.80 | 1.61 | 2.17 |
| Palestine | Western Europe | 419.17 | 1941.34 | 98.68 | 211.39 | 4.63 | 2.14 |
| Panama | Caribbean | 815.49 | 2076.93 | 123.93 | 235.13 | 2.55 | 1.90 |
| Papua New Guinea | North Africa and Middle East | 2936.81 | 13013.25 | 207.92 | 501.82 | 4.43 | 2.41 |
| Paraguay | Central Europe | 1242.91 | 3198.44 | 91.79 | 156.73 | 2.57 | 1.71 |
| Peru | Eastern Europe | 3206.57 | 7840.67 | 54.25 | 88.88 | 2.45 | 1.64 |
| Philippines | Eastern Sub-Saharan Africa | 19385.02 | 40586.19 | 71.53 | 110.37 | 2.09 | 1.54 |
| Poland | Caribbean | 3901.11 | 4179.41 | 79.32 | 104.27 | 1.07 | 1.31 |
| Portugal | Caribbean | 2155.20 | 3279.22 | 104.87 | 263.53 | 1.52 | 2.51 |
| Puerto Rico | Oceania | 1500.42 | 1728.86 | 168.45 | 339.27 | 1.15 | 2.01 |
| Qatar | Central sub-Saharan Africa | 99.69 | 1318.04 | 141.34 | 321.72 | 13.22 | 2.28 |
| Republic of Korea | North Africa and Middle East | 11920.66 | 22301.38 | 109.52 | 330.80 | 1.87 | 3.02 |
| Republic of Moldova | Western sub-Saharan Africa | 519.24 | 916.98 | 81.72 | 190.14 | 1.77 | 2.33 |
| Romania | Central Europe | 1855.56 | 1445.62 | 57.21 | 91.01 | 0.78 | 1.59 |
| Russian Federation | Southeast Asia | 12090.63 | 20513.63 | 58.71 | 117.94 | 1.70 | 2.01 |
| Rwanda | Western sub-Saharan Africa | 2801.59 | 3588.03 | 48.38 | 59.01 | 1.28 | 1.22 |
| Saint Kitts and Nevis | High-income Asia Pacific | 30.69 | 34.36 | 203.53 | 298.08 | 1.12 | 1.46 |
| Saint Lucia | Central Europe | 112.94 | 161.24 | 263.85 | 456.41 | 1.43 | 1.73 |
| Saint Vincent and the Grenadines | Central Europe | 96.08 | 110.22 | 234.73 | 434.41 | 1.15 | 1.85 |
| Samoa | Oceania | 89.55 | 281.30 | 295.37 | 734.87 | 3.14 | 2.49 |
| San Marino | Eastern Sub-Saharan Africa | 2.80 | 5.92 | 71.39 | 174.19 | 2.11 | 2.44 |
| Sao Tome and Principe | Southern sub-Saharan Africa | 18.99 | 98.63 | 81.05 | 191.31 | 5.19 | 2.36 |
| Saudi Arabia | High-income Asia Pacific | 2845.99 | 22896.56 | 126.71 | 318.29 | 8.05 | 2.51 |
| Senegal | Eastern Sub-Saharan Africa | 2584.34 | 9115.54 | 98.90 | 182.89 | 3.53 | 1.85 |
| Serbia | Western Europe | 1079.99 | 1011.53 | 81.82 | 120.60 | 0.94 | 1.47 |
| Seychelles | Southeast Asia | 13.32 | 47.67 | 80.86 | 280.60 | 3.58 | 3.47 |
| Sierra Leone | North Africa and Middle East | 889.15 | 4760.71 | 69.19 | 166.48 | 5.35 | 2.41 |
| Singapore | Caribbean | 833.70 | 2191.13 | 142.12 | 247.91 | 2.63 | 1.74 |
| Slovakia | Southern sub-Saharan Africa | 396.17 | 502.78 | 56.99 | 94.35 | 1.27 | 1.66 |
| Slovenia | Western Europe | 147.21 | 173.21 | 57.04 | 98.85 | 1.18 | 1.73 |
| Solomon Islands | Western Europe | 182.54 | 915.40 | 200.16 | 428.43 | 5.01 | 2.14 |
| Somalia | North Africa and Middle East | 2194.15 | 7626.06 | 54.75 | 101.76 | 3.48 | 1.86 |
| South Africa | East Asia | 15932.11 | 27247.87 | 103.41 | 148.00 | 1.71 | 1.43 |
| South Sudan | Central Asia | 1255.52 | 3642.80 | 43.38 | 83.99 | 2.90 | 1.94 |
| Spain | Eastern Sub-Saharan Africa | 5711.71 | 11953.87 | 83.01 | 237.24 | 2.09 | 2.86 |
| Sri Lanka | Southeast Asia | 5097.97 | 11267.02 | 103.67 | 268.83 | 2.21 | 2.59 |
| Sudan | Caribbean | 2750.70 | 16421.22 | 97.48 | 253.49 | 5.97 | 2.60 |
| Suriname | Western sub-Saharan Africa | 194.91 | 520.65 | 179.64 | 415.64 | 2.67 | 2.31 |
| Sweden | Southeast Asia | 1036.16 | 1782.39 | 89.98 | 160.16 | 1.72 | 1.78 |
| Switzerland | Western sub-Saharan Africa | 1199.82 | 2660.69 | 101.43 | 238.24 | 2.22 | 2.35 |
| Syrian Arab Republic | Oceania | 2653.39 | 5039.42 | 123.33 | 241.10 | 1.90 | 1.95 |
| Taiwan (Province of China) | Caribbean | 4832.36 | 5463.62 | 98.41 | 138.85 | 1.13 | 1.41 |
| Tajikistan | North Africa and Middle East | 736.35 | 2527.72 | 66.48 | 153.47 | 3.43 | 2.31 |
| Thailand | North Africa and Middle East | 12161.45 | 19364.58 | 69.67 | 134.42 | 1.59 | 1.93 |
| Timor-Leste | Central Asia | 115.68 | 357.27 | 54.26 | 151.14 | 3.09 | 2.79 |
| Togo | Eastern Sub-Saharan Africa | 820.17 | 3217.12 | 53.72 | 100.03 | 3.92 | 1.86 |
| Tokelau | Eastern Europe | 1.26 | 2.08 | 370.96 | 716.76 | 1.65 | 1.93 |
| Tonga | North Africa and Middle East | 64.71 | 125.17 | 301.04 | 616.28 | 1.93 | 2.05 |
| Trinidad and Tobago | Western Europe | 1001.39 | 1423.25 | 233.20 | 421.02 | 1.42 | 1.81 |
| Tunisia | High-income North America | 1114.42 | 4744.31 | 98.62 | 264.08 | 4.26 | 2.68 |
| Türkiye | Southern Latin America | 11990.80 | 25880.02 | 62.56 | 189.83 | 2.16 | 3.03 |
| Turkmenistan | Central Asia | 364.40 | 1679.65 | 53.99 | 146.75 | 4.61 | 2.72 |
| Tuvalu | Oceania | 7.86 | 13.92 | 236.70 | 516.73 | 1.77 | 2.18 |
| Uganda | Central Latin America | 2616.55 | 11721.08 | 37.11 | 69.58 | 4.48 | 1.87 |
| Ukraine | Southeast Asia | 5632.42 | 7376.01 | 69.74 | 138.09 | 1.31 | 1.98 |
| United Arab Emirates | Caribbean | 244.41 | 2268.39 | 99.46 | 216.52 | 9.28 | 2.18 |
| United Kingdom | North Africa and Middle East | 8296.39 | 31356.23 | 97.16 | 317.31 | 3.78 | 3.27 |
| United Republic of Tanzania | Eastern Sub-Saharan Africa | 5083.14 | 16859.86 | 36.14 | 78.27 | 3.32 | 2.17 |
| United States of America | Southern sub-Saharan Africa | 36018.71 | 82424.97 | 72.00 | 209.82 | 2.29 | 2.91 |
| United States Virgin Islands | Western Europe | 52.23 | 48.33 | 213.99 | 412.48 | 0.93 | 1.93 |
| Uruguay | Western Europe | 257.52 | 511.39 | 46.31 | 124.05 | 1.99 | 2.68 |
| Uzbekistan | Caribbean | 2557.51 | 11470.82 | 69.25 | 195.44 | 4.49 | 2.82 |
| Vanuatu | Oceania | 73.65 | 344.43 | 188.59 | 499.95 | 4.68 | 2.65 |
| Venezuela (Bolivarian Republic of) | Oceania | 6834.16 | 15408.73 | 130.11 | 239.99 | 2.25 | 1.84 |
| Viet Nam | Oceania | 13357.93 | 25503.44 | 62.40 | 104.84 | 1.91 | 1.68 |
| Yemen | Oceania | 1273.77 | 8306.04 | 82.04 | 181.25 | 6.52 | 2.21 |
| Zambia | Oceania | 2865.45 | 9486.77 | 76.56 | 148.64 | 3.31 | 1.94 |
| Zimbabwe | Oceania | 1414.81 | 5593.37 | 56.14 | 110.18 | 3.95 | 1.96 |
