# Supplementary material for: Assessing the global burden of Type 2 diabetes in women of reproductive age
Source: PLoS One. 2025 Jul 14;20(7):e0322787. doi: 10.1371/journal.pone.0322787 (PMC12258576; doi:10.1371/journal.pone.0322787)
Supplement: S5 Table — (DOCX) [file pone.0322787.s008.docx]

**Table S5. The Comparative Analysis of Age-Standardized type 2 diabetes mellitus Incidence and DALY Rates by Country and Region, 1990 and 2021.**

| Country | Region | DALYR_1990 | DALYR_2021 | ASIR_1990 | ASIR_2021 |
| --- | --- | --- | --- | --- | --- |
| Afghanistan | North Africa and Middle East | 187.85 | 520.92 | 186.52 | 575.54 |
| Albania | Central Europe | 31.27 | 44.28 | 54.42 | 95.69 |
| Algeria | North Africa and Middle East | 94.25 | 234.89 | 114.05 | 312.00 |
| American Samoa | Oceania | 413.07 | 1019.77 | 371.77 | 975.64 |
| Andorra | Western Europe | 59.43 | 123.00 | 70.54 | 172.80 |
| Angola | Central sub-Saharan Africa | 170.14 | 251.95 | 87.78 | 181.62 |
| Antigua and Barbuda | Caribbean | 262.63 | 304.98 | 179.26 | 307.62 |
| Argentina | Southern Latin America | 64.49 | 92.37 | 64.33 | 141.94 |
| Armenia | Central Asia | 79.91 | 116.24 | 86.49 | 155.07 |
| Australia | Australasia | 29.73 | 44.72 | 43.30 | 79.82 |
| Austria | Western Europe | 36.24 | 74.63 | 40.72 | 106.55 |
| Azerbaijan | Central Asia | 68.12 | 148.11 | 78.93 | 204.97 |
| Bahamas | North Africa and Middle East | 260.70 | 377.61 | 172.64 | 339.44 |
| Bahrain | South Asia | 164.17 | 302.46 | 123.62 | 296.53 |
| Bangladesh | Caribbean | 181.01 | 296.71 | 132.09 | 320.13 |
| Barbados | Eastern Europe | 254.57 | 340.17 | 157.45 | 297.38 |
| Belarus | Western Europe | 33.62 | 57.59 | 54.18 | 98.63 |
| Belgium | Caribbean | 68.55 | 143.61 | 77.20 | 184.85 |
| Belize | Western sub-Saharan Africa | 266.60 | 370.97 | 149.21 | 299.68 |
| Benin | Caribbean | 136.69 | 300.09 | 82.79 | 221.11 |
| Bermuda | South Asia | 120.91 | 199.07 | 97.02 | 198.01 |
| Bhutan | Andean Latin America | 116.31 | 166.70 | 111.70 | 201.95 |
| Bolivia (Plurinational State of) | Central Europe | 149.24 | 158.32 | 76.97 | 143.04 |
| Bosnia and Herzegovina | Southern sub-Saharan Africa | 45.06 | 76.57 | 61.01 | 123.76 |
| Botswana | Tropical Latin America | 84.85 | 144.98 | 56.64 | 119.72 |
| Brazil | High-income Asia Pacific | 169.30 | 141.14 | 98.73 | 141.20 |
| Brunei Darussalam | Central Europe | 236.56 | 320.92 | 130.84 | 383.61 |
| Bulgaria | Western sub-Saharan Africa | 55.58 | 75.80 | 72.79 | 119.49 |
| Burkina Faso | Eastern Sub-Saharan Africa | 131.88 | 211.44 | 53.91 | 133.14 |
| Burundi | Southeast Asia | 185.05 | 191.25 | 55.82 | 90.73 |
| Cabo Verde | Western sub-Saharan Africa | 123.37 | 246.27 | 85.60 | 185.47 |
| Cambodia | High-income North America | 121.46 | 150.79 | 60.70 | 130.06 |
| Cameroon | Western sub-Saharan Africa | 154.16 | 270.23 | 64.33 | 151.81 |
| Canada | Central sub-Saharan Africa | 13.62 | 37.62 | 25.41 | 106.78 |
| Central African Republic | Western sub-Saharan Africa | 214.31 | 364.16 | 118.52 | 272.53 |
| Chad | Southern Latin America | 119.08 | 248.77 | 68.17 | 153.28 |
| Chile | East Asia | 50.69 | 78.84 | 64.01 | 133.76 |
| China | Central Latin America | 103.13 | 228.74 | 121.27 | 266.01 |
| Colombia | Eastern Sub-Saharan Africa | 181.21 | 208.85 | 138.29 | 207.67 |
| Comoros | Central sub-Saharan Africa | 186.06 | 245.57 | 67.00 | 145.04 |
| Congo | Central Latin America | 195.64 | 305.03 | 85.83 | 180.68 |
| Cook Islands | Western sub-Saharan Africa | 584.12 | 815.41 | 433.27 | 841.13 |
| Costa Rica | Central Europe | 147.07 | 259.18 | 136.05 | 272.12 |
| Croatia | Caribbean | 38.63 | 52.35 | 59.27 | 101.66 |
| Cuba | Western Europe | 167.71 | 236.37 | 135.81 | 237.53 |
| Cyprus | Central Europe | 75.89 | 108.92 | 82.37 | 158.88 |
| Czechia | Central sub-Saharan Africa | 38.99 | 58.16 | 62.09 | 109.24 |
| Cote d'Ivoire | Western Europe | 128.05 | 264.97 | 75.24 | 167.19 |
| Democratic People's Republic of Korea | Eastern Sub-Saharan Africa | 102.61 | 186.21 | 98.20 | 201.47 |
| Democratic Republic of the Congo | Caribbean | 163.73 | 238.98 | 73.28 | 156.97 |
| Denmark | Caribbean | 39.84 | 82.97 | 55.55 | 133.33 |
| Djibouti | Andean Latin America | 102.14 | 156.70 | 43.11 | 86.51 |
| Dominica | North Africa and Middle East | 281.32 | 462.19 | 205.75 | 393.68 |
| Dominican Republic | Central Latin America | 194.75 | 359.47 | 134.89 | 309.19 |
| Ecuador | Central sub-Saharan Africa | 114.29 | 178.16 | 85.25 | 191.42 |
| Egypt | Eastern Sub-Saharan Africa | 113.47 | 218.96 | 60.55 | 223.93 |
| El Salvador | Eastern Europe | 156.89 | 274.47 | 112.56 | 236.45 |
| Equatorial Guinea | Eastern Sub-Saharan Africa | 180.28 | 278.45 | 86.57 | 193.36 |
| Eritrea | Oceania | 156.26 | 230.88 | 57.23 | 121.56 |
| Estonia | Oceania | 32.99 | 78.08 | 64.07 | 149.01 |
| Eswatini | Western Europe | 111.22 | 204.16 | 71.05 | 133.98 |
| Ethiopia | Western Europe | 288.98 | 191.85 | 82.73 | 110.67 |
| Fiji | Central sub-Saharan Africa | 589.54 | 1014.85 | 283.11 | 613.42 |
| Finland | Central Asia | 61.87 | 149.33 | 103.09 | 251.56 |
| France | Western Europe | 33.11 | 72.78 | 46.89 | 115.32 |
| Gabon | Western sub-Saharan Africa | 174.96 | 263.68 | 89.12 | 189.46 |
| Gambia | Western Europe | 121.51 | 250.99 | 65.32 | 150.43 |
| Georgia | High-income North America | 57.11 | 138.77 | 71.37 | 198.46 |
| Germany | Caribbean | 43.71 | 102.65 | 54.75 | 161.16 |
| Ghana | Oceania | 191.73 | 244.15 | 81.91 | 157.23 |
| Greece | Central Latin America | 81.32 | 168.04 | 92.36 | 204.53 |
| Greenland | Western sub-Saharan Africa | 34.26 | 55.34 | 15.49 | 76.85 |
| Grenada | Western sub-Saharan Africa | 515.48 | 490.89 | 212.04 | 370.48 |
| Guam | Caribbean | 217.93 | 357.12 | 188.48 | 370.62 |
| Guatemala | Caribbean | 197.40 | 446.47 | 137.64 | 352.01 |
| Guinea | Central Latin America | 139.42 | 258.78 | 64.35 | 135.96 |
| Guinea-Bissau | Central Europe | 224.02 | 357.36 | 95.47 | 197.82 |
| Guyana | Western Europe | 468.87 | 743.70 | 296.39 | 637.95 |
| Haiti | South Asia | 532.23 | 630.54 | 249.93 | 457.13 |
| Honduras | Southeast Asia | 186.81 | 274.56 | 145.28 | 283.21 |
| Hungary | North Africa and Middle East | 54.49 | 64.70 | 68.24 | 110.15 |
| Iceland | North Africa and Middle East | 57.89 | 132.46 | 72.53 | 184.56 |
| India | Western Europe | 116.67 | 190.58 | 115.46 | 227.31 |
| Indonesia | Western Europe | 121.47 | 136.47 | 78.01 | 137.33 |
| Iran (Islamic Republic of) | Western Europe | 64.78 | 145.42 | 74.36 | 189.48 |
| Iraq | Caribbean | 240.08 | 424.71 | 219.01 | 504.23 |
| Ireland | High-income Asia Pacific | 58.17 | 88.99 | 75.62 | 147.62 |
| Israel | North Africa and Middle East | 86.94 | 96.46 | 79.71 | 141.00 |
| Italy | Central Asia | 65.74 | 87.01 | 86.00 | 138.65 |
| Jamaica | Eastern Sub-Saharan Africa | 217.73 | 328.34 | 114.21 | 247.80 |
| Japan | Oceania | 62.79 | 118.57 | 82.38 | 152.93 |
| Jordan | North Africa and Middle East | 184.91 | 239.91 | 152.73 | 311.93 |
| Kazakhstan | Central Asia | 77.27 | 191.31 | 99.58 | 257.55 |
| Kenya | Southeast Asia | 100.12 | 120.50 | 43.55 | 63.83 |
| Kiribati | Eastern Europe | 661.07 | 1027.83 | 368.43 | 689.96 |
| Kuwait | North Africa and Middle East | 138.86 | 288.24 | 169.40 | 399.04 |
| Kyrgyzstan | Southern sub-Saharan Africa | 54.72 | 113.44 | 64.94 | 158.39 |
| Lao People's Democratic Republic | Western sub-Saharan Africa | 178.47 | 227.38 | 86.84 | 189.90 |
| Latvia | North Africa and Middle East | 41.65 | 109.81 | 64.01 | 148.57 |
| Lebanon | Eastern Europe | 152.03 | 233.52 | 129.01 | 275.89 |
| Lesotho | Western Europe | 57.08 | 163.67 | 44.96 | 112.95 |
| Liberia | Central Europe | 144.50 | 301.11 | 72.86 | 167.16 |
| Libya | Eastern Sub-Saharan Africa | 86.27 | 220.59 | 103.61 | 285.52 |
| Lithuania | Eastern Sub-Saharan Africa | 29.94 | 70.40 | 56.12 | 128.31 |
| Luxembourg | Southeast Asia | 62.41 | 125.13 | 76.53 | 176.78 |
| Madagascar | Southeast Asia | 177.89 | 206.06 | 51.53 | 92.05 |
| Malawi | Western sub-Saharan Africa | 143.93 | 144.94 | 39.99 | 60.33 |
| Malaysia | Western Europe | 115.89 | 166.68 | 106.40 | 181.37 |
| Maldives | Oceania | 148.68 | 118.73 | 80.08 | 136.05 |
| Mali | Western sub-Saharan Africa | 237.20 | 401.97 | 125.36 | 257.30 |
| Malta | Southeast Asia | 59.95 | 145.29 | 70.30 | 219.38 |
| Marshall Islands | Central Latin America | 628.07 | 1646.05 | 437.78 | 1117.56 |
| Mauritania | Eastern Europe | 119.12 | 168.20 | 60.27 | 112.68 |
| Mauritius | Central Asia | 146.14 | 394.03 | 124.71 | 307.10 |
| Mexico | Central Europe | 365.88 | 415.58 | 281.02 | 401.41 |
| Micronesia (Federated States of) | North Africa and Middle East | 410.90 | 780.35 | 281.53 | 611.46 |
| Monaco | Eastern Sub-Saharan Africa | 55.03 | 123.00 | 67.12 | 171.08 |
| Mongolia | Southeast Asia | 52.87 | 115.20 | 60.53 | 151.50 |
| Montenegro | Southern sub-Saharan Africa | 42.88 | 62.86 | 76.92 | 130.91 |
| Morocco | South Asia | 106.74 | 333.23 | 140.23 | 468.21 |
| Mozambique | Western Europe | 127.45 | 180.82 | 39.79 | 90.82 |
| Myanmar | Australasia | 396.10 | 319.00 | 134.36 | 239.96 |
| Namibia | Central Latin America | 88.86 | 129.63 | 60.49 | 102.76 |
| Nauru | Western sub-Saharan Africa | 537.77 | 967.73 | 334.83 | 703.09 |
| Nepal | Western sub-Saharan Africa | 139.90 | 266.59 | 128.31 | 287.01 |
| Netherlands | East Asia | 36.93 | 84.83 | 50.44 | 124.85 |
| New Zealand | Oceania | 47.04 | 101.88 | 69.09 | 130.16 |
| Nicaragua | Western Europe | 205.77 | 274.04 | 151.83 | 268.06 |
| Niger | North Africa and Middle East | 118.22 | 209.05 | 73.99 | 150.42 |
| Nigeria | South Asia | 113.62 | 145.03 | 62.53 | 100.53 |
| Niue | North Africa and Middle East | 391.53 | 1014.39 | 330.80 | 790.55 |
| North Macedonia | Central Latin America | 53.73 | 67.39 | 66.77 | 121.93 |
| Northern Mariana Islands | Oceania | 290.42 | 420.26 | 202.41 | 403.68 |
| Norway | Tropical Latin America | 66.25 | 93.10 | 96.96 | 141.62 |
| Oman | Andean Latin America | 88.24 | 177.41 | 115.12 | 229.02 |
| Pakistan | Southeast Asia | 134.37 | 285.87 | 125.83 | 278.26 |
| Palau | Central Europe | 407.37 | 848.28 | 351.69 | 763.80 |
| Palestine | Western Europe | 130.03 | 198.17 | 98.68 | 211.39 |
| Panama | Caribbean | 175.24 | 257.96 | 123.93 | 235.13 |
| Papua New Guinea | North Africa and Middle East | 412.67 | 642.52 | 207.92 | 501.82 |
| Paraguay | Central Europe | 170.07 | 214.79 | 91.79 | 156.73 |
| Peru | Eastern Europe | 78.41 | 104.28 | 54.25 | 88.88 |
| Philippines | Eastern Sub-Saharan Africa | 164.20 | 183.22 | 71.53 | 110.37 |
| Poland | Caribbean | 47.29 | 54.57 | 79.32 | 104.27 |
| Portugal | Caribbean | 113.82 | 203.68 | 104.87 | 263.53 |
| Puerto Rico | Oceania | 207.54 | 314.45 | 168.45 | 339.27 |
| Qatar | Central sub-Saharan Africa | 141.09 | 253.41 | 141.34 | 321.72 |
| Republic of Korea | North Africa and Middle East | 117.95 | 266.03 | 109.52 | 330.80 |
| Republic of Moldova | Western sub-Saharan Africa | 53.55 | 118.36 | 81.72 | 190.14 |
| Romania | Central Europe | 41.92 | 46.85 | 57.21 | 91.01 |
| Russian Federation | Southeast Asia | 37.58 | 68.75 | 58.71 | 117.94 |
| Rwanda | Western sub-Saharan Africa | 221.88 | 131.56 | 48.38 | 59.01 |
| Saint Kitts and Nevis | High-income Asia Pacific | 376.54 | 285.52 | 203.53 | 298.08 |
| Saint Lucia | Central Europe | 442.01 | 466.81 | 263.85 | 456.41 |
| Saint Vincent and the Grenadines | Central Europe | 474.42 | 531.79 | 234.73 | 434.41 |
| Samoa | Oceania | 341.54 | 782.06 | 295.37 | 734.87 |
| San Marino | Eastern Sub-Saharan Africa | 59.04 | 123.94 | 71.39 | 174.19 |
| Sao Tome and Principe | Southern sub-Saharan Africa | 97.30 | 229.14 | 81.05 | 191.31 |
| Saudi Arabia | High-income Asia Pacific | 117.87 | 262.74 | 126.71 | 318.29 |
| Senegal | Eastern Sub-Saharan Africa | 190.48 | 297.86 | 98.90 | 182.89 |
| Serbia | Western Europe | 58.37 | 62.54 | 81.82 | 120.60 |
| Seychelles | Southeast Asia | 97.71 | 263.23 | 80.86 | 280.60 |
| Sierra Leone | North Africa and Middle East | 109.84 | 264.40 | 69.19 | 166.48 |
| Singapore | Caribbean | 108.75 | 188.70 | 142.12 | 247.91 |
| Slovakia | Southern sub-Saharan Africa | 36.05 | 47.67 | 56.99 | 94.35 |
| Slovenia | Western Europe | 35.98 | 50.33 | 57.04 | 98.85 |
| Solomon Islands | Western Europe | 360.42 | 722.83 | 200.16 | 428.43 |
| Somalia | North Africa and Middle East | 165.94 | 214.24 | 54.75 | 101.76 |
| South Africa | East Asia | 217.74 | 214.76 | 103.41 | 148.00 |
| South Sudan | Central Asia | 130.97 | 209.65 | 43.38 | 83.99 |
| Spain | Eastern Sub-Saharan Africa | 79.45 | 173.46 | 83.01 | 237.24 |
| Sri Lanka | Southeast Asia | 142.22 | 266.07 | 103.67 | 268.83 |
| Sudan | Caribbean | 78.74 | 188.78 | 97.48 | 253.49 |
| Suriname | Western sub-Saharan Africa | 271.28 | 470.38 | 179.64 | 415.64 |
| Sweden | Southeast Asia | 69.03 | 103.45 | 89.98 | 160.16 |
| Switzerland | Western sub-Saharan Africa | 88.29 | 172.92 | 101.43 | 238.24 |
| Syrian Arab Republic | Oceania | 131.82 | 193.41 | 123.33 | 241.10 |
| Taiwan (Province of China) | Caribbean | 104.73 | 131.46 | 98.41 | 138.85 |
| Tajikistan | North Africa and Middle East | 77.33 | 123.42 | 66.48 | 153.47 |
| Thailand | North Africa and Middle East | 97.65 | 163.56 | 69.67 | 134.42 |
| Timor-Leste | Central Asia | 79.85 | 144.20 | 54.26 | 151.14 |
| Togo | Eastern Sub-Saharan Africa | 120.85 | 188.80 | 53.72 | 100.03 |
| Tokelau | Eastern Europe | 460.64 | 883.45 | 370.96 | 716.76 |
| Tonga | North Africa and Middle East | 428.70 | 677.77 | 301.04 | 616.28 |
| Trinidad and Tobago | Western Europe | 416.80 | 513.23 | 233.20 | 421.02 |
| Tunisia | High-income North America | 73.19 | 193.74 | 98.62 | 264.08 |
| Türkiye | Southern Latin America | 110.11 | 157.56 | 62.56 | 189.83 |
| Turkmenistan | Central Asia | 51.09 | 173.82 | 53.99 | 146.75 |
| Tuvalu | Oceania | 409.79 | 645.55 | 236.70 | 516.73 |
| Uganda | Central Latin America | 93.00 | 149.82 | 37.11 | 69.58 |
| Ukraine | Southeast Asia | 54.43 | 84.75 | 69.74 | 138.09 |
| United Arab Emirates | Caribbean | 81.43 | 156.92 | 99.46 | 216.52 |
| United Kingdom | North Africa and Middle East | 77.54 | 265.36 | 97.16 | 317.31 |
| United Republic of Tanzania | Eastern Sub-Saharan Africa | 112.77 | 150.96 | 36.14 | 78.27 |
| United States of America | Southern sub-Saharan Africa | 64.83 | 140.88 | 72.00 | 209.82 |
| United States Virgin Islands | Western Europe | 245.84 | 379.54 | 213.99 | 412.48 |
| Uruguay | Western Europe | 45.13 | 81.36 | 46.31 | 124.05 |
| Uzbekistan | Caribbean | 64.46 | 158.07 | 69.25 | 195.44 |
| Vanuatu | Oceania | 278.09 | 589.45 | 188.59 | 499.95 |
| Venezuela (Bolivarian Republic of) | Oceania | 180.01 | 292.57 | 130.11 | 239.99 |
| Viet Nam | Oceania | 100.82 | 121.44 | 62.40 | 104.84 |
| Yemen | Oceania | 60.57 | 128.71 | 82.04 | 181.25 |
| Zambia | Oceania | 214.81 | 251.59 | 76.56 | 148.64 |
| Zimbabwe | Oceania | 76.47 | 176.11 | 56.14 | 110.18 |
