# Supplementary material for: Assessing the global burden of Type 2 diabetes in women of reproductive age
Source: PLoS One. 2025 Jul 14;20(7):e0322787. doi: 10.1371/journal.pone.0322787 (PMC12258576; doi:10.1371/journal.pone.0322787)
Supplement: S6 Table — (DOCX) [file pone.0322787.s009.docx]

**Table S6. The top three and the bottom three regions of type 2 diabetes mellitus burden in women of childbearing age.**

| Measure | Top three countries |  |  | Bottom three countries |  |  |
| --- | --- | --- | --- | --- | --- | --- |
| 2021 ASR (per 100,000 people) |  |  |  |  |  |  |
| Age-standardized DALY rate | Central Latin America(345.43) | Caribbean(428.48) | Oceania(681.20) | Australasia(54.15) | Central Europe(56.47) | Eastern Europe(72.94) |
| ASIR | Central Latin America(326.13) | Caribbean(350.50) | Oceania(516.42) | Australasia(88.37) | Eastern Sub-Saharan Africa(90.03) | Central Europe(105.44) |
| 1990-2021 increase times |  |  |  |  |  |  |
| DALY (cases) | Central sub-Saharan Africa(3.90) | Western sub-Saharan Africa(4.21) | North Africa and Middle East(4.55) | Central Europe(1.01) | Tropical Latin America(1.31) | Eastern Europe(1.58) |
| Incidence(case) | Western sub-Saharan Africa(5.32) | Central sub-Saharan Africa(5.66) | North Africa and Middle East(5.96) | Central Europe(1.24) | East Asia(1.59) | Eastern Europe(1.70) |
| EAPC |  |  |  |  |  |  |
| DALY | High-income Asia Pacific(2.60) | Western Europe(2.71) | East Asia(2.71) | Tropical Latin America(-0.72) | Eastern Sub-Saharan Africa(-0.43) | Southeast Asia(-0.22) |
| Incidence | Central Asia(3.12) | North Africa and Middle East(3.44) | High-income North America(3.64) | Southeast Asia(0.95) | Tropical Latin America(1.16) | Southern sub-Saharan Africa(1.19) |
