# Supplementary material for: Assessing the global burden of Type 2 diabetes in women of reproductive age
Source: PLoS One. 2025 Jul 14;20(7):e0322787. doi: 10.1371/journal.pone.0322787 (PMC12258576; doi:10.1371/journal.pone.0322787)
Supplement: S7 Table — (DOCX) [file pone.0322787.s010.docx]

**Table S7. The top three and the bottom three countries of type 2 diabetes mellitus burden in women of childbearing age.**

| Measure | Top three countries |  |  | Bottom three countries |  |  |
| --- | --- | --- | --- | --- | --- | --- |
| 2021 ASR (per 100,000 people) |  |  |  |  |  |  |
| Age-standardized DALY rate | American Samoa(1019.77) | Kiribati(1027.83) | Marshall Islands(1646.05) | Cameroon(37.62) | Albania(44.28) | Australia(44.72) |
| ASIR | Congo(841.13) | American Samoa(975.64) | Marshall Islands(1117.56) | Rwanda(59.01) | Malawi(60.33) | Kenya(63.83) |
| 1990-2021 increase times |  |  |  |  |  |  |
| DALY (cases) | United Arab Emirates(9.28) | Afghanistan(9.92) | Qatar(13.22) | Romania(0.78) | Hungary(0.90) | United States Virgin Islands(0.93) |
| Incidence(case) | United Arab Emirates(9.91) | Afghanistan(11.06) | Qatar(15.47) | Romania(1.08) | United States Virgin Islands(1.13) | Bulgaria(1.17) |
| EAPC |  |  |  |  |  |  |
| DALY | Turkmenistan(4.21) | Mauritius(4.24) | Lesotho(4.40) | Rwanda(-2.67) | Ethiopia(-2.01) | Myanmar(-1.24) |
| Incidence | Cameroon(4.30) | Egypt(4.41) | Greenland(5.37) | Indonesia(-0.53) | Rwanda(0.15) | Ethiopia(0.67) |
